# CLOK: a chemigenetic multicolor labeling system to visualize neuronal birthdate and circuit integration

**DOI:** 10.1038/s41593-026-02423-9

**Published:** 2026-09-30

**Authors:** Giulia Faini, Matthieu Tuffery, Amna Saleem, Lixia Zhang, Felix Du, Guillaume Le Bourdelles, Karine Duroure, Eric R. Schreiter, Dimitrii Tanese, Valentina Emiliani, Filippo Del Bene, Minoru Koyama

**Affiliations:** 1 Institut de la Vision, Sorbonne Université, Inserm S968, CNRS UMR7210, Paris, France; 2 Department of Biological Sciences, University of Toronto Scarborough, Toronto, Ontario Canada; 3 Janelia Research Campus, Howard Hughes Medical Institute, Ashburn, VA USA; 4 Department of Cell and Systems Biology, University of Toronto, Toronto, Ontario Canada; 5 Present Address: Advanced Imaging and Microscopy Resource, National Institutes of Health, Bethesda, MD USA

**Keywords:** Developmental neurogenesis, Neural circuits, Ca2+ imaging, Fluorescence imaging, Development of the nervous system

## Abstract

Understanding how neurons integrate into developing circuits and contribute to functional activity is essential for decoding brain development and plasticity. However, current methods to study neuronal integration often suffer from low throughput, limited spatiotemporal resolution or invasive procedures. To overcome these challenges, here we present CLOK, an in vivo birthdate-labeling strategy based on HaloTag technology and its broad palette of fluorescent ligands. We show that CLOK enables precise color-coding of neurons and their subcellular structures by birthdate and allows tracking of functional maturation of newborn neurons in the developing zebrafish visual and motor systems, revealing distinct maturation trajectories of early- and late-born neurons. We further demonstrate the versatility of this approach by coupling it to optical tools enabling age-specific multicolor calcium and voltage imaging, as well as optogenetic manipulation. Altogether, CLOK provides a powerful and flexible tool for optical interrogation of neuronal circuit maturation in a growing and behaving vertebrate.

## Abstract (web summary)

Faini et al. develop CLOK, a method that labels neurons across a spectrum of colors according to when they were born, allowing successive generations of brain cells to be tracked as they take shape in living zebrafish.

## Main

Understanding the developmental trajectory of individual neurons and the maturation of their roles within neural circuits is essential to elucidate brain development and plasticity during both embryonic and postembryonic growth, when progressively more refined functions emerge. The integration of neurons into developing circuits has been extensively investigated across vertebrate models^1–3^. Newly generated neurons are gradually incorporated into existing circuits by extending axons and dendrites and forming synaptic connections^4^. In several regions of the central nervous system, neurogenesis is spatially organized, with newborn neurons being progressively added in topologically distinct areas. Examples include the spinal cord^5^, hippocampus^6^, cortex and cerebellum^7^. In this context, the zebrafish larva offers a particularly advantageous model for studying neurogenesis and functional integration owing to its transparency and rapid development^3,8^. Importantly, a similar topographical organization is observed in the zebrafish nervous system, where some regions exhibit a highly ordered structure characterized by a developmental gradient^9–11^. A striking example is the optic tectum (OT), the main visual center in the fish brain: neurons generated early in development (early-born) occupy central regions, while newly generated neurons (late-born) are progressively added to peripheral regions throughout the animal’s life^12,13^.

Previous studies were limited by low throughput and methodological constraints, underscoring the need for more advanced tools to examine neurogenesis and functional integration at the population level, ideally in vivo. Indeed, a comprehensive functional and causal study of neurogenesis and neuronal integration requires a method capable of identifying the maturation stage of individual neurons and distinguishing early-born from late-born neurons within the intact, developing brain. Furthermore, such an approach should also enable the monitoring of neuronal activity patterns, particularly in relation to specific behaviors or sensory inputs, and permit noninvasive manipulation of neuronal activity. This capability would facilitate precise correlations between neuronal activation, downstream circuit dynamics and behavioral outcomes.

Ex vivo approaches to investigate neurogenesis typically involve the use of bromo-deoxyuridine (BrdU), ethynyl-deoxyuridine (EdU) or similar thymidine-analogs, which, when delivered to a tissue, are incorporated into the DNA of cells undergoing replication^14,15^. This allows the identification and analysis of the fate of cells born during the labeling period and their subsequent offspring. However, because these approaches require tissue fixation for post hoc staining, they are not applicable to functional studies in living animals.

Multiple in vivo approaches have been developed so far, each with substantial caveats. One relies on sparse labeling of progenitors by DNA electroporation or heat shock, allowing researchers to monitor the growth, integration and migration of their progeny within developing circuits^16,17^; this approach is inherently low throughput and labor intensive and often involves invasive procedures. More recently, CRISPR-based strategies have been developed to activate predetermined sequences of genetic reporters in dividing progenitors, enabling birthdating of multiple age cohorts^18–20^; these require substantial optimization owing to their complexity, and even under optimal conditions, reporter switching is inefficient. A third approach, used in optically accessible models such as zebrafish, expresses a photoconvertible fluorescent protein in differentiating neurons, distinguishing neurons born after photoconversion^21,22^; although applicable broadly across a genetically defined population, it cannot resolve neurons born within a defined period. A challenge common to such fluorescence-based methods is that combining them with functional tools such as optogenetics^23^ or activity imaging^24^ is hampered by overlapping excitation and emission spectra^25^, reducing the accuracy and reliability of both birthdating and functional readouts.

To overcome these limitations, we developed CLOK (Chromatic halotag Labeling for cell Ontogeny tracKing), a chemigenetic method for reliable age-dependent neuronal labeling in vivo. CLOK is based on an original framework that exploits the self-labeling HaloTag protein^26^ together with Janelia Fluor (JF) dyes, fluorescent ligands known for their excellent photostability and cell permeability^27,28^. HaloTag is expressed in differentiating neurons, and distinct JF dyes are applied sequentially over time, so that neurons born within a given interval are labeled by a distinct dye, enabling precise birthdate tagging. After validating this approach against thymidine-based birthdating, we introduce genetic strategies that enable age-dependent labeling of neuronal processes, simultaneous birthdating of up to five neuronal cohorts, long-term tracking over 2 months and functional characterization of visual and motor responses in early- and late-born neurons. We further combined CLOK with chemigenetic optical tools—calcium and voltage indicators and opsins—to directly record and manipulate the activity of birthdate-defined cohorts. Overall, CLOK addresses key limitations in in vivo neuronal birthdating, expands the anatomical and functional readouts of emerging neuronal populations and enables their functional manipulation, providing a powerful tool to probe neural development, circuit integration and plasticity.

## Results

### CLOK provides a clear view of brain-wide topographical organization based on neuronal age

On exposure to a cell-permeable, fast-binding JF dye ligand, neurons genetically expressing HaloTag become permanently fluorescently labeled, enabling precise visualization of targeted cells^27,29^ or, as recently shown, identification of newly inserted receptors or synaptic proteins^30,31^. We first performed a series of control experiments to determine key features and parameters of the CLOK labeling. In a first set of experiments, we examined if all genetically targeted neurons could be labeled with a JF dye in larval zebrafish using a transgenic (Tg) line co-expressing the HaloTag protein and a membrane-localized GFP. After incubation with a JF dye (here, JF_525_) at 5 days post fertilization (d.p.f.) (Methods), we evaluated the degree of colocalization of the dye and GFP fluorescence, finding 97.43 ± 1.8% of co-expressing neurons (Supplementary Fig. 1a; mean ± s.d., *N* = 3 fish, 16 planes), confirming robust, near-complete JF dye labeling. Delaying imaging after dye incubation in the same Tg line (incubation at 3 d.p.f. followed by imaging from 3 to 6 d.p.f.) increased the number of JF dye-negative, GFP-positive cells located at the periphery of the tectum (Supplementary Fig. 1b). This observation confirms that the HaloTag-JF dye complex remains stable, preventing JF dye from binding to newly expressed HaloTag protein and thereby enabling temporally targeted HaloTag labeling. We then characterized how well JF dye binds HaloTag across developmental stages and remains fluorescent thereafter, using larvae expressing nuclear-localized HaloTag under a pan-neuronal promoter (Methods). Dye incubations were performed at different time points ranging between 0 and 5 d.p.f. and the OT was imaged from 1 to 6 d.p.f. At all time points, JF dye was successfully incorporated in neurons across the brain, and the labeled neurons remained fluorescent throughout, confirming effective JF dye loading across the developmental stages (Supplementary Fig. 1c). In addition, we assessed, following dye incubation, the clearance of unincorporated dye and the resulting reduction of nonspecific fluorescence, after periods ranging from 0 to 24 h. We observed, for several JF dyes, a gradual decrease in the background signal over time (below 20% after 30 min and below 10% after 2 h), resulting in an almost total washout after 4 h (Supplementary Fig. 2). Finally, we examined potential developmental effects of loading single or multiple JF dyes early in development and found no clear effect on the survival rate, the swim bladder development and the body length, indicating negligible developmental effects of JF dye loading (Supplementary Fig. 3; Methods).

**Fig. 1:**
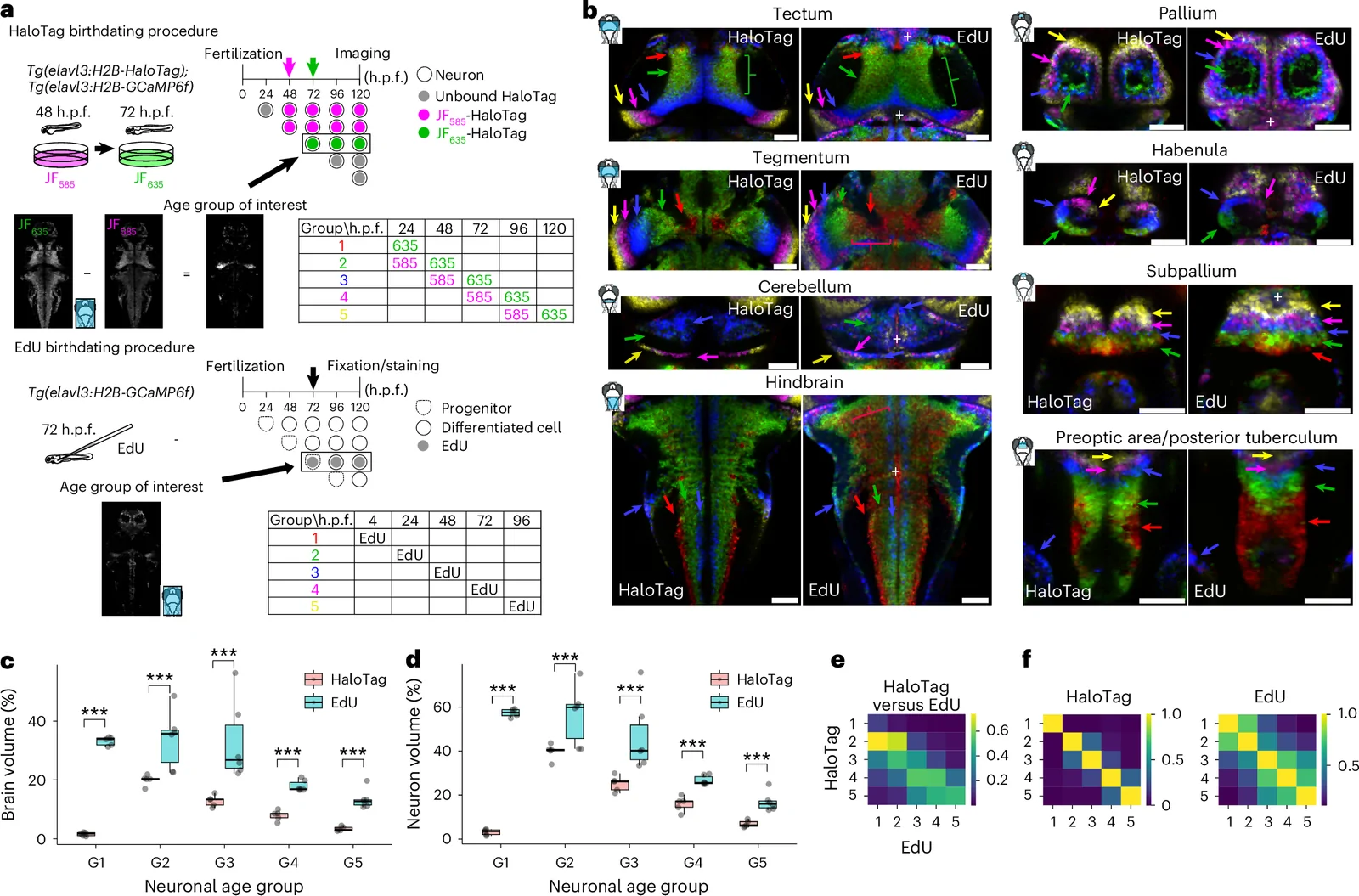
CLOK and its comparison with EdU birthdating in zebrafish. **a**, CLOK validation strategy. HaloTag labeling procedure: *Tg(elavl3:H2B-HaloTag)* was used to express HaloTag protein in the nuclei of developing neurons. Two JF dyes were loaded in sequence to visualize neurons born in specific time windows. Differential volumes were created from normalized dye signals to visualize the age group of interest (Methods). The table describes the dye loading schedules and indicates the color used for each group. EdU birthdating procedures: EdU was injected into *Tg(elavl3:H2B-GCaMP6f)* individuals from 4 to 96 h.p.f. The table describes the color used for each group. Both datasets were registered to the ZBB atlas using the nuclear GCaMP signal to create age group maps in both procedures (*N* = 5 or 6 fish per group). **b**, Side-by-side comparisons of CLOK birthdating and EdU staining across the zebrafish brain. The location of the brain region is indicated by a cyan patch overlaid on the fish. Single optical slices in the same atlas space are shown for comparison. Colored arrows highlight neuronal age groups. White plus signs indicate the ventricular zone. Scale bars, 50 µm. **c**, Percentage brain volume occupied by each age group (five or six fish per target age group). Box plots show the median (center line), the 25th and 75th percentiles (lower and upper hinges of the box) and whiskers extending to the most extreme data point within 1.5× the interquartile range of the hinge. ***P < 0.001, Tukey’s post hoc test after per-voxel binomial regression (G1, *z* = 5,933.6; G2, *z* = 3,235.1; G3, *z* = 4,832.6; G4, *z* = 3,039.8; G5, *z* = 3,426.2). **d**, Percentage of neuron volume occupied by each age group (five or six fish per target age group). Box plots are prepared as in **c**. Ventricular and neuropil zones are excluded from quantification. ***P < 0.001; Tukey’s post hoc test after per-voxel binomial regression (G1, *z* = 6,115.4, *P* < 0.001; G2, *z* = 2,445.3; G3, *z* = 3,243.5; G4, *z* = 1,889.6; G5, *z* = 2,121.6). **e**, Cross-correlation of the neuron volume occupied by each age group across CLOK and EdU data**. f**, Cross-correlation of the neuron volume occupied by each age group within each dataset. G, group. Source data

**Extended Data Fig. 1:**
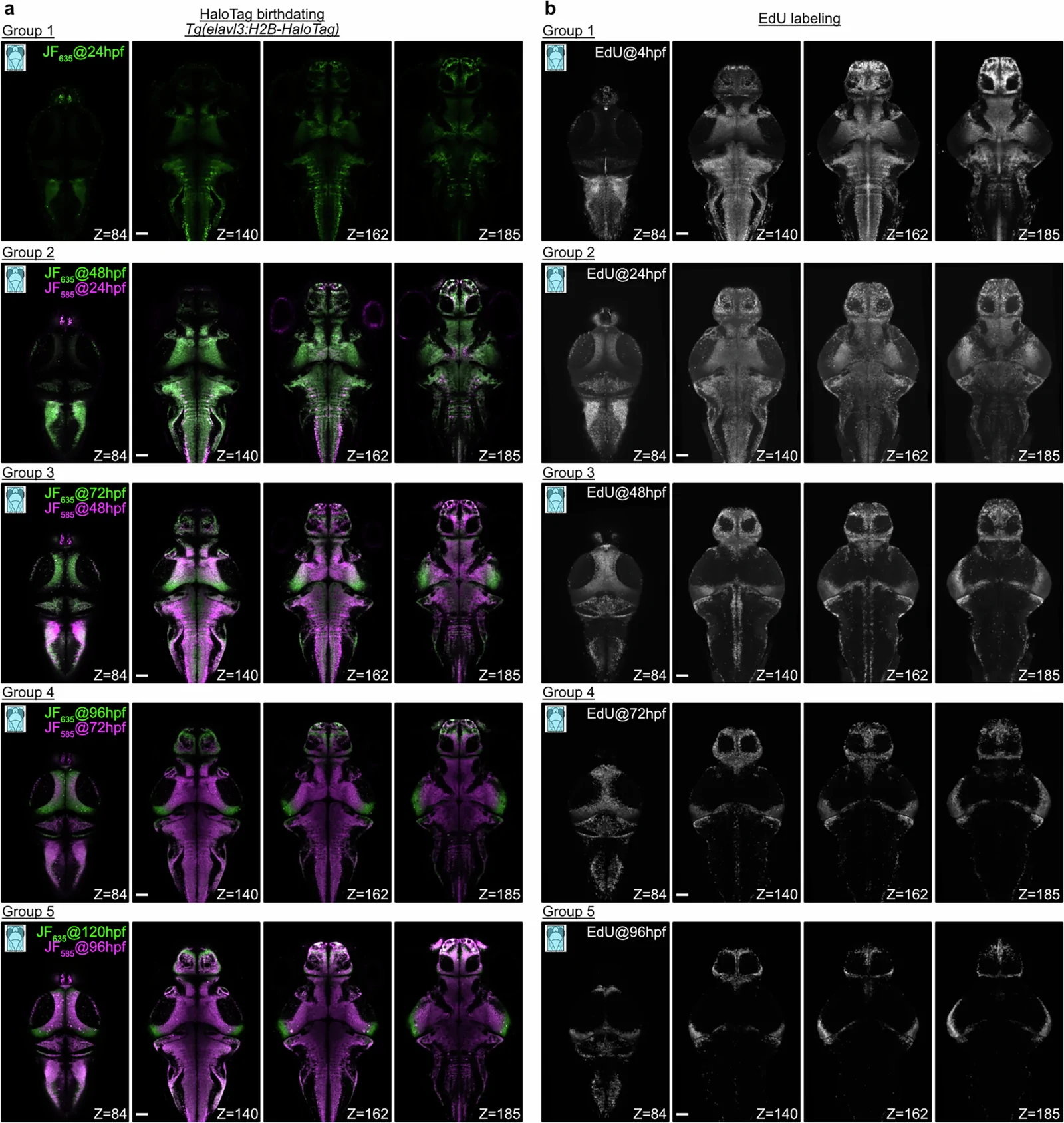
Individual age groups labeled by single-pulse CLOK and EdU labeling. **a**, Horizontal slices showing neurons birthdated with single-pulse CLOK labeling for five-time windows in *Tg(elavl3:H2B-HaloTag)*. Group 1: 0-24 hpf; Group 2: 24-48 hpf; Group 3: 48-72 hpf; Group 4: 72-96 hpf; Group 5: 96-120 hpf. JF_585_ labels the neurons before the time window of interest. JF_635_ labels the neurons born during the time window of interest. The Z value in each horizontal slice indicates the dorsoventral position in the ZBB space. Each volume is a median of 5 or 6 subjects. **b**, Horizontal slices showing the cells labeled by EdU introduced at 4 hpf (Group 1), 24 hpf (Group 2), 48 hpf (Group 3), 72 hpf (Group 4), and 96 hpf (Group 5). Horizontal slices are organized as in a. Each volume is a median of more than 5 or 6 subjects. Scale bars, 50 µm.

**Extended Data Fig. 2:**
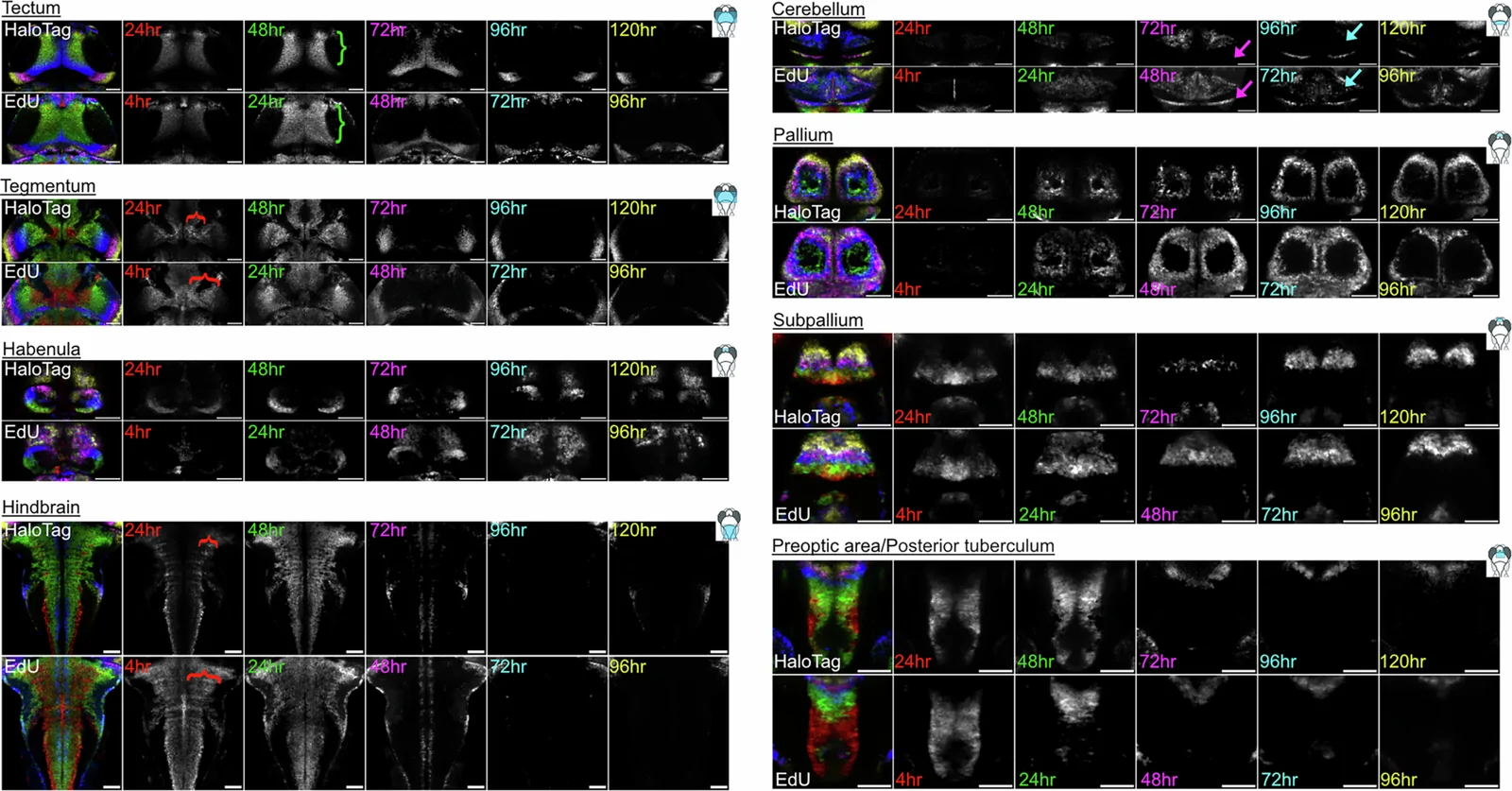
Grayscale images of each neuronal age group in CLOK and EdU dataset. Grayscale images of each neuronal age group are shown in the brain areas highlighted in Fig. 1. Each image is a median of more than 5 or 6 subjects. Curly braces indicate the extent of the labeled population. Arrows indicate the differential labeling patterns. Scale bars: 50 µm.

**Extended Data Fig. 3:**
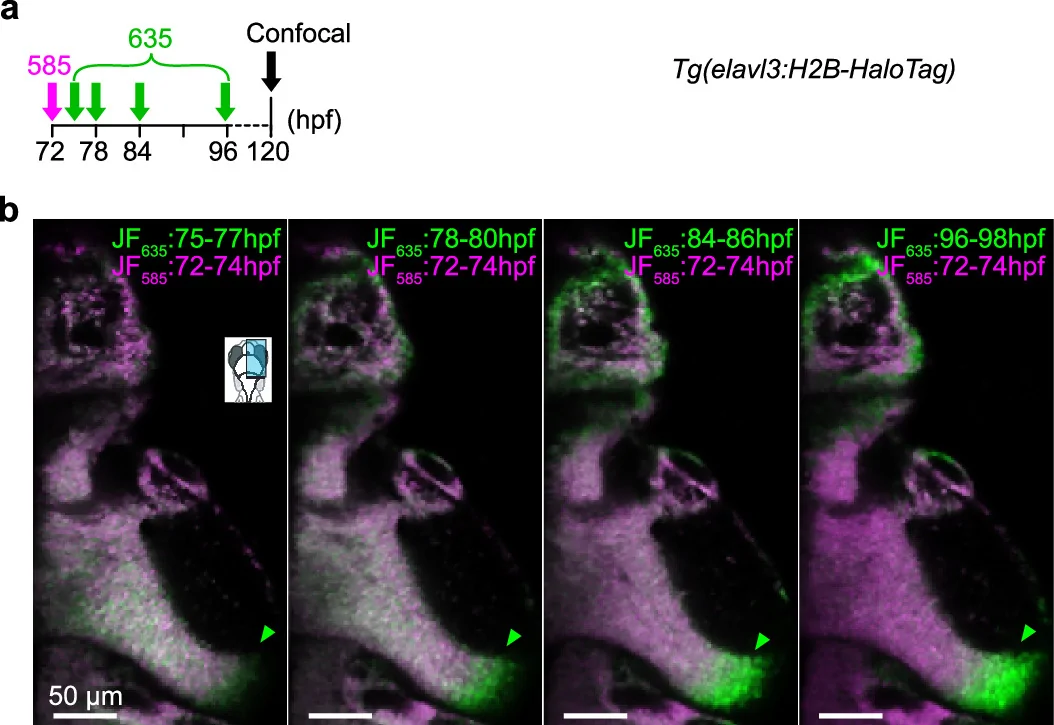
Short-pulse CLOK labeling. **a**, Dye loading schedule for short interval CLOK labeling. 585: JF_585_, 635: JF_635_**. b**, Single-optical slices showing newborn neurons labeled in each dye loading schedule. Each volume is a median of registered and normalized volumes from 4 subjects. Magenta: normalized JF_585_ signal, Green: normalized JF_635_ signal. Green arrowheads indicate newborn neurons labeled by JF_635_. Scale bars: 50 µm.

Neurogenesis studies often rely on the use of thymidine analogs such as BrdU or EdU, which are incorporated into the DNA of dividing cells during the S phase^32^. These markers require tissue fixation for postlabeling staining. Here, we set out to compare CLOK against EdU staining by birthdating cells every 24 h and comparing their distribution at 5 d.p.f. To do so, we generated Tg lines that express the HaloTag protein using the *elavl3* promoter, a well-established marker of early neuronal differentiation^33,34^. To enable direct comparison of the two approaches, we implemented the following strategy (Fig. 1a). First, we added a nuclear localization sequence to the HaloTag to mimic EdU’s nuclear signal. Second, we targeted a single age cohort per animal using two distinct JF dyes (cohort neurons were defined as those showing a stronger signal from the second dye; Methods). Third, using the same Tg background *Tg(elavl3:H2B-GCaMP6f)*, we registered both datasets to the Zebrafish Brain Browser (ZBB) atlas on the basis of live microscopy volumes and generated a three-dimensional (3D) volume for each treatment group by averaging registered volumes across larvae (Extended Data Fig. 1; *N* = 5 or 6 fish per target). To visualize targeted neuronal age groups in CLOK data, we normalized each dye signal per fish, subtracted the first dye signal from the second and averaged the resulting volumes across fish to generate a volume for each age group (Methods; Fig. 1a). The resulting subtracted volume looks similar across a range of normalization parameters (Supplementary Fig. 4), supporting the robustness of this visualization procedure. Both birthdating procedures revealed spatially clustered age-defined neuronal populations, uncovering topographical organization based on birthdate in more brain regions than previously reported^10,13^ (Fig. 1b). The labeling patterns were largely consistent across the brain, although with a few notable exceptions. First, EdU staining also targeted non-neuronal cell types, including progenitor cells in the ventricular zone (Fig. 1b, plus signs). Second, the spatial distribution of the EdU-labeled cells appeared broader (Fig. 1b, green and red curly braces in the tectum and the hindbrain, respectively; see Extended Data Fig. 2 for a grayscale image of each channel). These differences are expected, as EdU gets incorporated into all dividing cells and their offspring, while CLOK targets selectively differentiating neurons. To quantitatively evaluate each birthdating approach’s resolution, we implemented a protocol using the overall distribution of the color ratio rather than simple subtraction. The color ratio is defined as the angle of a two-dimensional (2D) vector represented by normalized dye signals, and each voxel is classified into two populations on the basis of this ratio using a Gaussian Mixture Model (GMM; Supplementary Fig. 5a; Methods). We then evaluated the area occupied by each age group within the brain volume and within the volume excluding the ventricular zone (Supplementary Fig. 5b). In both cases, birthdating method had a significant main effect on the volume labeled (Fig. 1c,d, binomial regression, *P* < 2.2 × 10^−16^). Post hoc tests also show that CLOK labels more specific populations across age groups (Fig. 1c,d, *P* < 0.001). Cross-correlation analyses within the neuronal nuclei volume also confirmed this observation (Fig. 1e,f). Although CLOK and EdU showed similar topographic organization (Fig. 1e, see higher correlation values in near diagonal elements), CLOK showed much reduced correlation across age groups compared with EdU, supporting the higher birthdating resolution of CLOK (Fig. 1f). We further examined whether the broad EdU labeling stemmed from the warping required to register fixed EdU samples to the ZBB atlas, which is based on live microscopy volumes. We repeated the same quantifications after registering both datasets to the MapZebrain atlas^35^, which is based on fixed microscopy volumes (Supplementary Fig. 6). All statistical effects described above remained significant, confirming that warping contributes negligibly to the observed difference in birthdating resolution. Motivated by the superior birthdating resolution of CLOK, we further examined its resolution limit by loading the first and the second dyes in shorter intervals (Extended Data Fig. 3). Neurons captured by CLOK begin to appear with a 3-h pulse labeling and are clearly detectable with a 6-h pulse labeling, suggesting that, for the *elavl3* promoter, CLOK has temporal resolution of 6 h or less. Having established that the CLOK strategy offers clearly superior birthdating resolution compared with the standard EdU method, we further examined its unique applications.

### CLOK reveals brain-wide chronological organization at subcellular level and enables simultaneous labeling of multiple neuronal age groups

One advantage of CLOK is its ability to visualize specific subcellular compartments of a target neuronal age group by fusing a localization sequence to the HaloTag protein. This is in contrast to the thymidine-based birthdating approach where the signal is restricted to the nucleus. Here, we targeted axon terminals using the synaptophysin tag^36^ and somatodendritic processes using the Voltron1 construct containing the HaloTag protein fused with the Kv2.1 localization sequence^37^. We targeted the same neuronal age groups as in Fig. 1 and generated the population average for each age group (*N* = 5 or 6 fish per group). The area occupied by each age group was visualized on the basis of the subtraction of normalized signals as in Fig. 1. This procedure enables visualization of the newly innervated area.

**Fig. 2:**
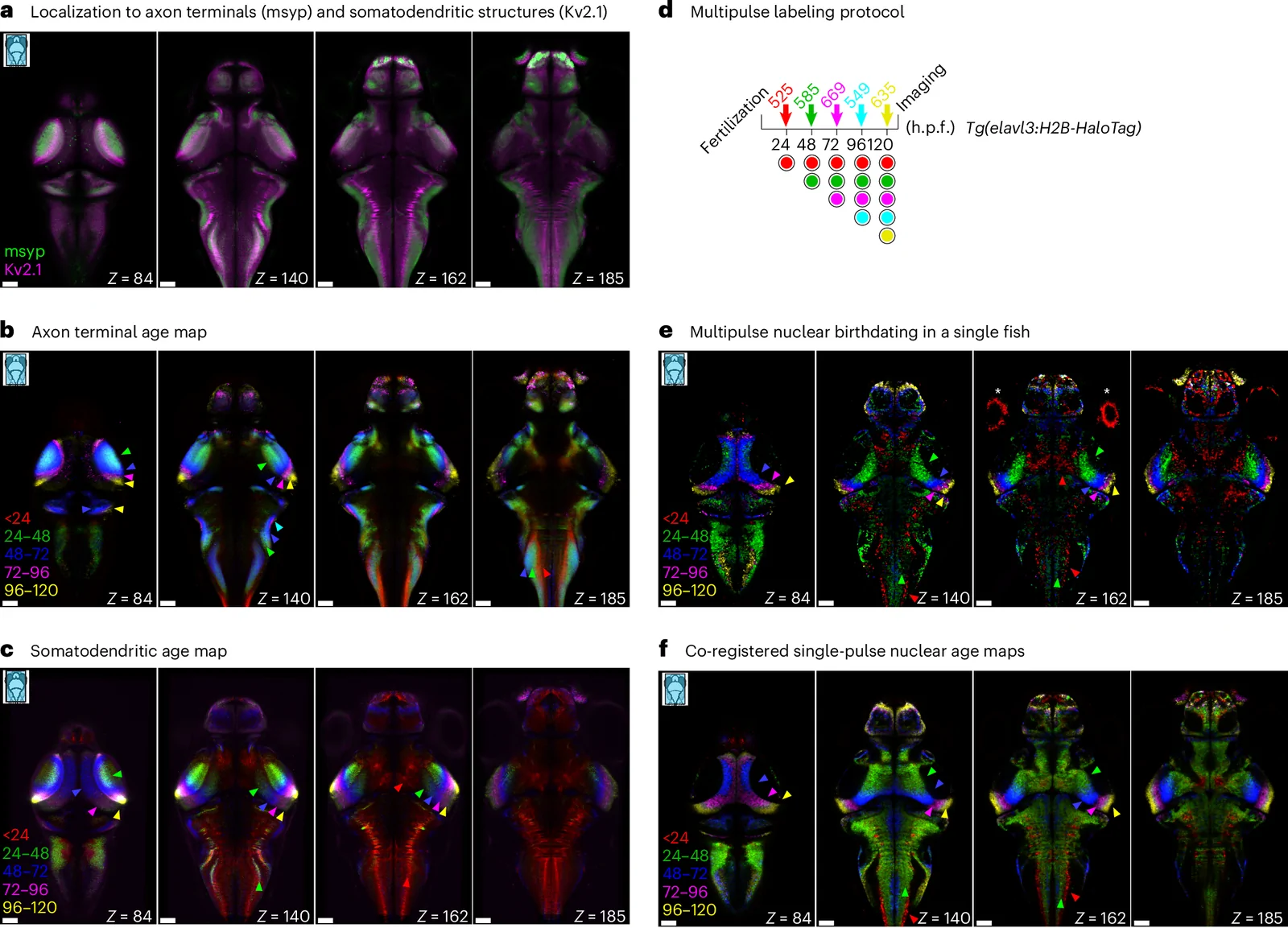
Subcellular visualization of neuronal age groups and simultaneous visualization of multiple neuronal age groups by CLOK. **a**, Distribution of HaloTag-JF_585_ localized to axon terminals (green, *Tg(elavl3:msyp-HaloTag*)) and somatodendritic processes (magenta, *Tg(elavl3:Voltron1-SOM2)*) shown in the ZBB atlas space. Fish were labeled with JF_585_ at 4 d.p.f. and imaged at 5 d.p.f. (*N* = 5 or 6 fish per group). Single-optical slices of registered volumes are shown at specified *z* coordinates (84, 140, 162 and 185) in the ZBB atlas. **b**, Visualization of new axon terminal areas occupied by each age group at the same *z* coordinates (*N* = 5 or 6 fish per group). Each age group is color coded as in Fig. 1. Red: <24 h.p.f., green: 24–48 h.p.f., magenta: 48–72 h.p.f., cyan: 72–96 h.p.f., yellow: 96–120 h.p.f. Colored arrowhead indicates the area occupied by each age group. Scale bars, 50 µm. The following panels are organized similarly. **c**, Visualization of new dendritic areas occupied by each age group (*N* = 5 or 6 fish per age group). **d**, Experimental timeline for multipulse birthdating in a single fish. 525, JF_525_; 585, JF_585_; 669, JF_669_; 549, JF_549_; 635, JF_635_. **e**, Visualization of five age groups in a single fish. The volume is registered to the ZBB atlas for the ease of comparison. Each age group is color coded as in Fig. 1. Red: <24 h.p.f., green: 24–48 h.p.f., magenta: 48–72 h.p.f., cyan: 72–96 h.p.f., yellow: 96–120 h.p.f. White asterisks indicate the autofluorescence from the eyes. **f**, Co-registered single-pulse nuclear age maps from Fig. 1 shown as a reference.

**Extended Data Fig. 4:**
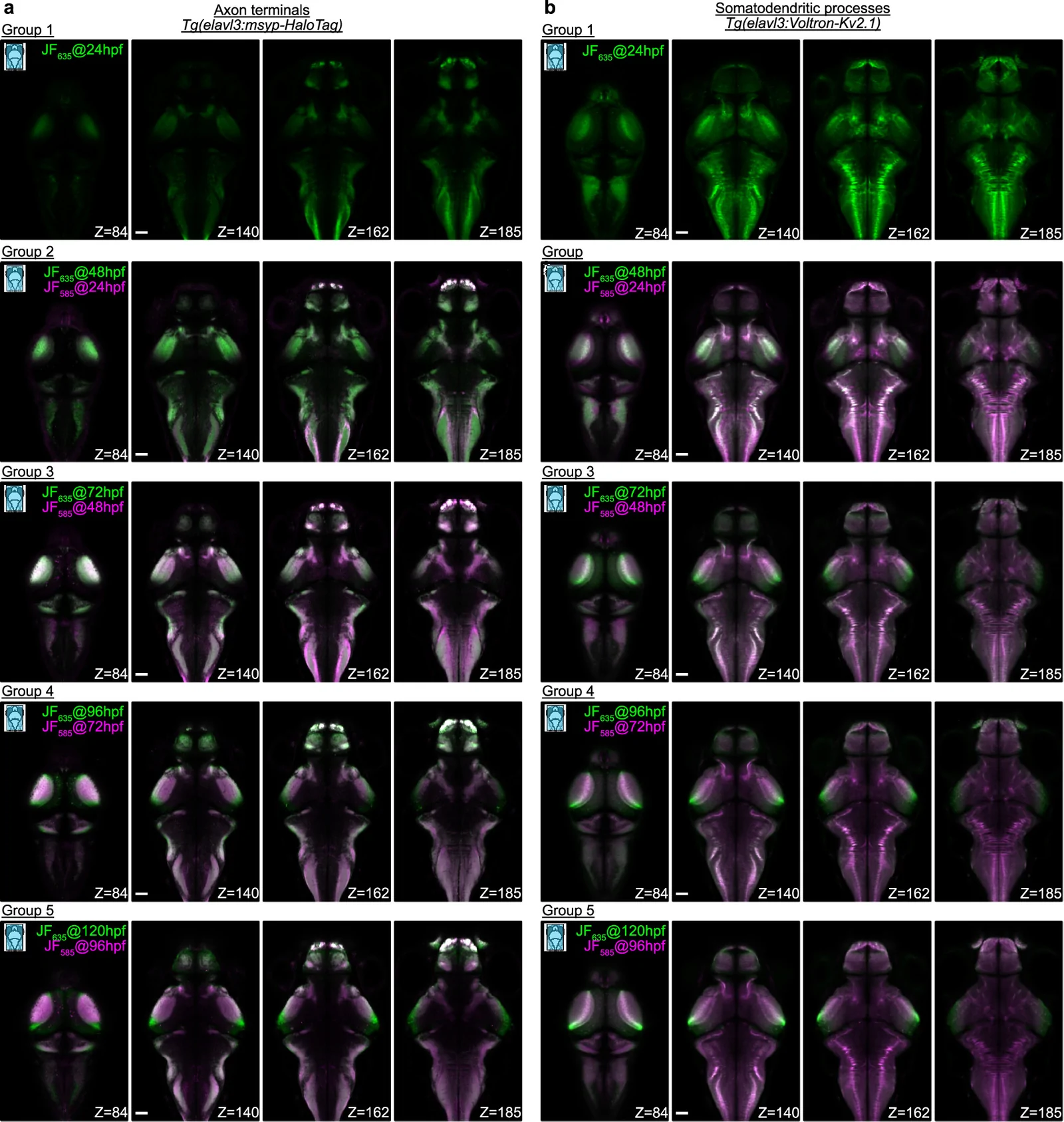
Subcellular processes visualized by single-pulse HaloTag birthdating. **a**, Axon terminals visualized by single-pulse HaloTag birthdating. Group 1: 0- < 24 hpf; Group 2: 24-48 hpf; Group 3: 48-72 hpf; Group 4: 72-96 hpf; Group 5: 96-120 hpf. JF_585_ was loaded before the time window of interest. JF_635_ was loaded at the end of the time window of interest. The Z value in each horizontal slice indicates the dorsoventral position in the ZBB space. Each volume is a median of 5 or 6 subjects. **b**, Somatodendritic processes visualized by single-pulse HaloTag birthdating. Images are organized as in a. Each volume is a median of more than 5 subjects. Scale bars: 50 µm.

We first confirmed the distinct labeling patterns of the two subcellular localization tags (axon terminals and somatodendritic processes) by comparing the JF_585_ signal in group 5, which captures the majority of neurons except those born between 4 and 5 d.p.f. (Fig. 2a). Interestingly, this comparison revealed not only the expected differential labeling in the cell body areas but also distinct domains within the neuropil that are dominated by either axonal terminals or dendrites. Furthermore, CLOK-based labeling of these subcellular tags revealed distinct age-dependent organization within these domains (Fig. 2b,c; see JF_585_ and JF_635_ signals before subtraction in Extended Data Fig. 4). We observed that axon terminals from the later-born populations occupy progressively the outer layer of the neuropil area across multiple brain regions such as the tectum, cerebellum and hindbrain (Fig. 2b, see the ordering of colored arrowheads). By contrast, the majority of dendritic domains were colored by the earliest-born group (<24; Fig. 2c) except the tectum. Closer inspection of JF_585_ and JF_635_ signals across age groups suggests that this pattern reflects the mostly overlapping or smaller dendritic processes of the late-born populations (Extended Data Fig. 4b). Taken together, CLOK labeling of subcellular processes revealed a brain-wide chronological organization of axonal and dendritic structures, highlighting a unique advantage of CLOK to visualize the subcellular architecture of birthdated neurons.

Another advantage of CLOK is the broad spectrum of JF dyes biocompatible in zebrafish. This characteristic provides an original opportunity to simultaneously visualize multiple neuronal age cohorts in individual fish. As a proof of principle, we applied five distinct JF dyes, one every 24 h, and imaged the larvae at 5 d.p.f. with a white laser confocal microscope to excite each dye at its optimal wavelength (Fig. 2d; Methods). We then visualized each neuronal age group by subtracting normalized signals as in Fig. 1 (Fig. 2e; Methods). Despite the differences in lactone–zwitterion equilibrium that influence biocompatibility of the dyes^38^, this approach successfully captured the overall chronological organization of nuclei positions observed in the population-level dataset (Fig. 2f). In summary, we demonstrated a unique capability of CLOK to simultaneously visualize five age-defined neuronal populations in a live animal.

Together, these results show that CLOK resolves the topographic organization of neurons by age—across subcellular compartments and across multiple cohorts—within the same developing animal.

### CLOK enables persistent, stable and bright labeling across juvenile stages

**Fig. 3:**
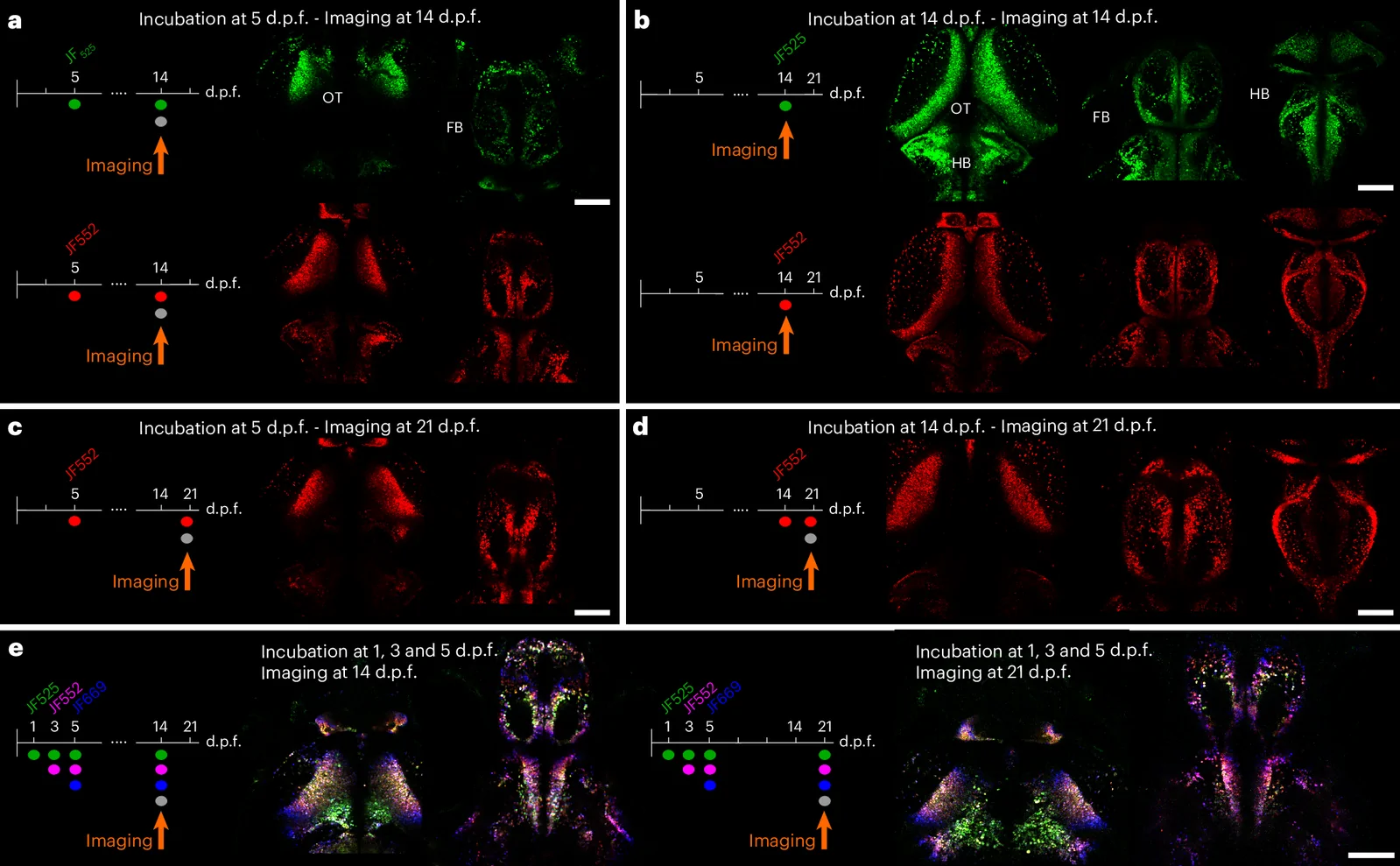
Persistent JF dye labeling in juvenile brains with CLOK. Confocal images showing the distribution of HaloTag-expressing neurons in *Tg(Xla.Tubb2-hsp70-ubc:H2B-HaloTag)* zebrafish larvae, with dye incubation and imaging performed at different developmental time points (see corresponding schematics). **a**, Image showing 14 d.p.f. larva labeled with JF_525_ (green) or JF_552_ (red) at 5 d.p.f. **b**, Same as in **a** but in larvae incubated at 14 d.p.f. **c**, Image showing 21 d.p.f. larva labeled with JF_552_ at 5 d.p.f. **d**, Image showing 21 d.p.f. larva labeled with JF_552_ at 14 d.p.f. **e**, Multilabeled larvae (at 1, 3 and 5 d.p.f.) imaged at 14 d.p.f. (left) or 21 d.p.f. (right). Scale bar, 100 µm. HB, hindbrain; FB, forebrain. *N* = 3 fish per experiment.

**Extended Data Fig. 5:**
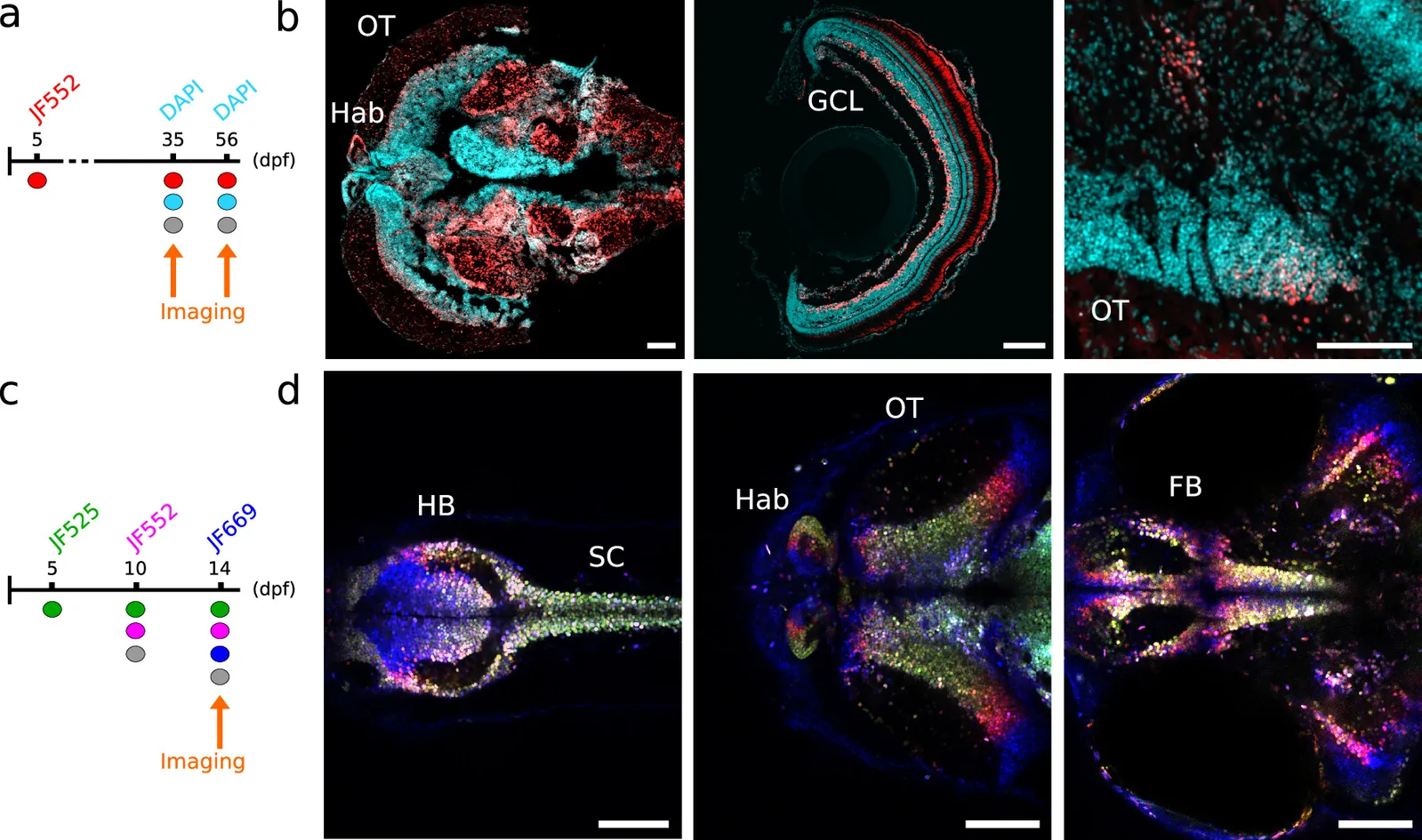
Single and multiple JF dyes labeling in juvenile stages revealed in fixed tissues and in vivo. **a**, Protocol of dye incubation and confocal imaging. The dye has been added at 5 dpf and tissue fixation was performed at 35 dpf or 56 dpf. **b**, Left, middle: Cryosections (16μm thickness) from a 5 weeks-old larva (fixation at 35 dpf). N = 3 fish. Right: Cryosections (16μm thickness) from an 8 weeks-old larva (fixation at 56 dpf). N = 2 fish. Different regions are shown: brain and retina. Red: JF_552_ labeling, showing expression of H2B-HaloTag. DAPI, in cyan stains neuronal soma (and non neuronal cells) born between 5dpf and the time of tissue fixation (35 or 56 dpf). **c**, Protocol of multilabelling and confocal imaging. Dyes have been added at 5 dpf (JF_525_, green), 10 dpf (JF_552_, magenta) and 14 dpf (JF_669_, blue). **d**, Multilabeled larvae imaged at 14 dpf. Different regions are shown: brain and spinal cord. N = 3 fish. Tg line used: Xla.Tubb2-hsp70-ubc:H2B-HaloTag. Scale bars: 100μm. OT: optic tectum. Hab: habenula. GCL: ganglion cell layer. HB: hindbrain. SC: spinal cord. FB: forebrain.

To assess the stability and persistence of the HaloTag labeling, we extended our analysis to juvenile stages and tracked neurons with single or multiple dyes in *Tg(Xla.Tubb2-hsp70-ubc:H2B-HaloTag)*. HaloTag-based labeling remained clearly detectable for up to 8 weeks after fertilization, enabling long-term visualization of neurons well beyond their initial integration into circuits (Extended Data Fig. 5). Larvae incubated with JF_525_ (green) or JF_552_ (red) at 5 d.p.f. retained clear labeling when imaged at 14 or 21 d.p.f. (Fig. 3a,c), and the dyes were also successfully incorporated at juvenile stages (incubation at 14 d.p.f.; Fig. 3b,d). Importantly, triple incubations at larval stages (1, 3 and 5 d.p.f.) still produced distinct birthdate labeling when imaged at 14 or 21 d.p.f. (Fig. 3e), confirming the temporal resolution and persistence of the method. This stable labeling also enabled identification of neurons in fixed and cryosectioned brains and retinas (Extended Data Fig. 5a,b), demonstrating compatibility of CLOK with histological analysis. In addition, HaloTag stability over weeks was confirmed by comparing transient expression of HaloTag alone or fused with the stable histone protein H2B^39^; both conditions retained detectable JF signal 2 weeks after dye loading (Supplementary Fig. 7). Finally, sequential labeling at 5, 10 and 14 d.p.f. (larvae imaged at 14 d.p.f.; Extended Data Fig. 5c,d) revealed topographic neuronal organization in juvenile fish similar to that observed during larval stages (Fig. 1b).

In sum, these results highlight the versatility of CLOK for longitudinal studies of neuronal maturation and integration, from early development into juvenile stages.

### CLOK allows tracking of the maturation of visual responses in early- and late-born tectal neurons

**Fig. 4:**
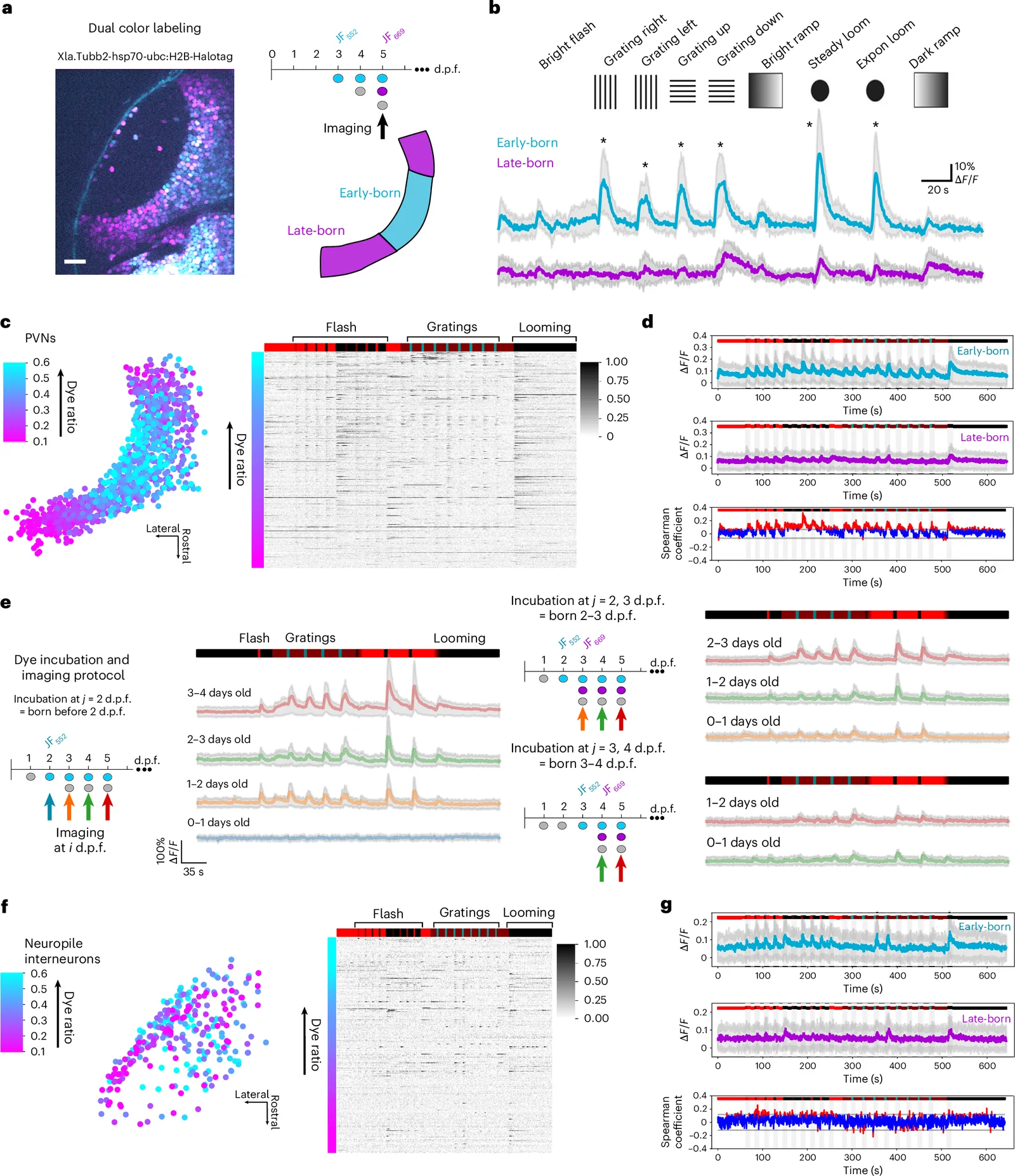
Visual activity of early- and late-born tectal neurons with CLOK. **a**, Dual-color labeling in *Tg(Xla.Tubb2-hsp70-ubc:H2B-HaloTag)*; *Tg(elavl3:H2B-GCaMP6s)* at two different time points during development (3 and 5 d.p.f.) and fluorescence dual color images of the larval OT. Early-born neurons are in cyan and late-born neurons in magenta. The schematic on the right represents the corresponding ROI used to integrate the calcium activity of the two populations. Scale bar, 25 µm. *N* = 5 larvae. **b**, Averaged GCaMP6s calcium traces of early-born (cyan) and late-born (magenta) neurons in response to a series of visual stimuli (top row), including flashes, gratings and looming stimuli. GCaMP fluorescence signals were integrated from the regions in **a**. Colored lines represent group mean, and gray areas represent s.d. (*N* = 8 larvae). Asterisks represent the visual responses that are significantly different between early and late-born populations (*P < 0.05, Welch’s *t*-test). **c**, Left: spatial distribution of PVNs color coded according to the ratio of fluorescence intensity of the two dyes JF_552_/(JF_552_ + JF_669_). Right: raster plot of Δ*F*/*F* traces from individual cells (*N* = 5 larvae). Larvae were presented with visual stimuli, schematically represented by the upper color bar (red meaning high visual intensity, black indicating dark and blue indicating moving stimuli; see the Methods for details). **d**, Top and middle: Δ*F*/*F* average traces from the early-born (cyan) and late-born (magenta) subpopulation of cells from **c**. Semitransparent rectangles indicate timing of visual stimuli. Traces are presented as the mean (colored lines) and the range between the 16th and 84th percentiles (gray areas). Bottom: Spearman coefficient between dye ratio and Δ*F*/*F*, plotted as a function of time. Sections in which the activity of the two populations show statistically significant differences are shown in red (threshold for significance: 0.0662, gray line; see the Methods for more details). **e**, For each of the three subpanels, left: schematic of the labeling protocol for either single-dye labeling (JF_552_ at *j* = 2 d.p.f.; **i**) or dual-dye labeling (JF_552_ and JF_669_ incubated at days *j* and *j* + 1 d.p.f., with *j* = 3 and 4). Functional imaging was performed at *i* ≥ *j* d.p.f. during the delivery of visual stimuli sequences (same as in **b**). Cells labeled only with JF_552_ define populations born either before 2 d.p.f. (**i**) or between days *j* and *j* + 1 and are thus (*i* − *j*) days old at the time of imaging. Right: average calcium responses of these age-defined populations to the visual stimuli sequence, shown for different incubation and imaging time points, corresponding to neuronal populations of varying ages (*N* = 4 larvae). Traces are presented as the mean (colored lines) and the range between the 16th and 84th percentiles (gray areas). **f**, Spatial distribution of neurons in the neuropile, color coded according to the ratio of fluorescence intensity of the two dyes (*N* = 9 larvae). Light-blue color represents early-born neurons. Right: raster plot of Δ*F*/*F* traces from individual cells (*N* = 5 fish). Larvae were presented with visual stimuli, schematically represented by the upper color bar (red meaning high visual intensity, black indicating dark; see the Methods for details). **g**, Top and middle: Δ*F*/*F* average traces from the early-born (cyan) and late-born (magenta) subpopulations of cells from **f**. Semitransparent rectangles indicate the timing of visual stimuli. Traces are presented as the mean (colored lines) and the range between the 16th and 84th percentiles (gray areas). Bottom: Spearman coefficient between dye ratio and Δ*F*/*F*, plotted as a function of time. Sections in which the activities of the two birthdated subpopulations show statistically significant differences are shown in red (threshold for significance: 0.118, gray line). Source data

**Extended Data Fig. 6:**
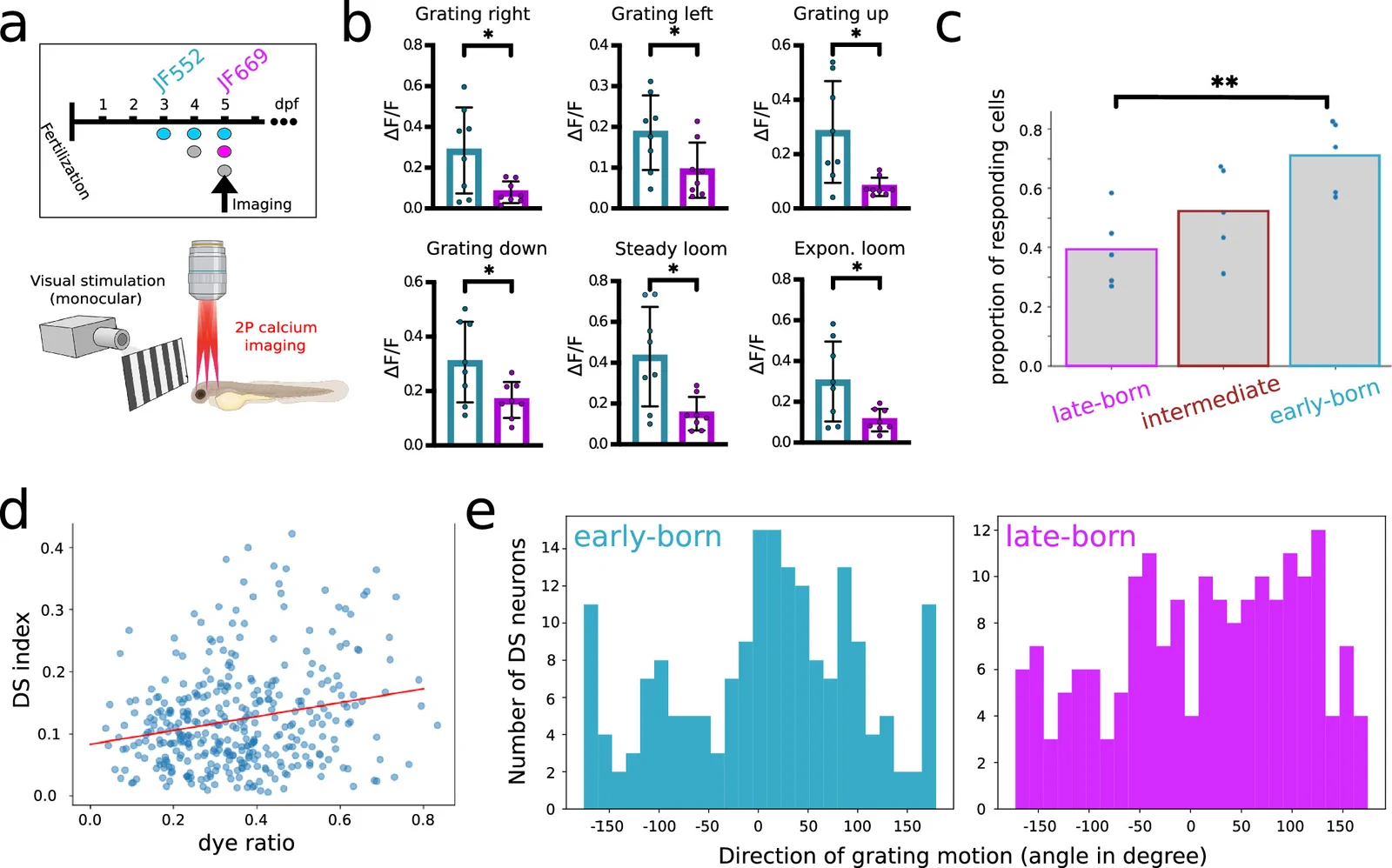
Visual activity of early- and late-born neurons. **a**, Experimental pipeline. Top: dual-color labeling in tg(Xla.Tubb2-hsp70-ubc:H2B-HaloTag; elavl3:H2B-GCaMP6s) at 2 different time points during development (2 and 5 dpf). Bottom: Schematic of the experimental setup, consisting in recording calcium activity under 2-photon illumination, while presenting a set of visual stimuli to the larvae, embedded in agar. **b**, Quantification of ΔF/F peak responses between early and late-born neurons, showing significant differences in response amplitudes for select visual stimuli, particularly moving gratings and looming disks (N = 8 larvae). **c**, Proportion of responsive neurons (defined as neurons with ΔF/F > 0.4) in three equal-sized groups based on dye ratio (late-born, intermediate and early-born neurons). **p < 0.01, 1-way ANOVA (N = 5 larvae). **d**, Direction selectivity (DS) index as a function of the dye ratio of individual neurons (p < 0.001, Spearman coefficient; R^2^ = 0.05, N = 5 larvae). **e**, Distribution of preferred direction of moving for early-born (cyan) and late-born (magenta) neurons (N = 5 larvae). Source data

To explore the functional integration of early- and late-born neurons, we combined CLOK birthdate labeling with visually evoked in vivo calcium imaging in the OT. We co-expressed the HaloTag protein and the calcium indicator GCaMP6s (*Tg(Xla.Tubb2-hsp70-ubc:H2B-HaloTag)*; *Tg(elavl3:H2B-GCaMP6s)*) and performed sequential incubation with JF_552_ and JF_669_ to label neurons on the basis of their birthdate (before 2 d.p.f. or between 2 and 5 d.p.f.; Fig. 4a and Extended Data Fig. 6a). These time points were selected because they correspond to known waves of tectal neurogenesis^12,13^. Head-fixed larvae were subsequently presented with a battery of visual stimuli while two-photon (2P) calcium imaging was performed in the contralateral OT (Methods).

We first examined visual responses of periventricular neurons (PVNs), the main neuronal population of the larval OT, whose somata reside in its deepest layer. Using dual-color CLOK labeling, we assessed birthdate-dependent responses through two complementary strategies. In a first macro-level approach (Fig. 4a,b), regions labeled exclusively with JF_669_ but lacking the first dye JF_552_ were classified as late-born (neurons generated between the first and second dye loading), whereas centrally located regions labeled with both dyes (neurons born before the first dye loading) were classified as early-born (Methods). This spatial distribution—peripheral for late-born neurons and central for early-born—matches the known progression of tectal neurogenesis^12,13^. Integrating GCaMP signals across these macro-regions revealed distinct differences in visually evoked activity (Fig. 4b and Extended Data Fig. 6b). Early-born neurons (more mature) generally displayed stronger responses than late-born neurons. Specifically, bright flash elicited similar responses in both populations, suggesting comparable baseline visual sensitivity. By contrast, moving gratings and looming stimuli evoked significantly greater responses in the early-born population (*P < 0.05, Welch’s *t*-test; Extended Data Fig. 6b). This macro-region approach is simple, fast and convenient when birthdate-defined populations form spatially distinct clusters or when cellular-resolution imaging is unavailable.

We next performed single-cell analysis using a broader stimulus set, including dark and bright flashes, moving gratings and looming stimuli (Methods; Fig. 4c,d). Neurons were ordered according to the dual-dye intensity ratio JF_552_/(JF_552_ + JF_669_), which serves as a proxy for neuronal birthdate. As the promoter is constitutively active in postmitotic cells, this ratio reflects the relative time elapsed since neuronal differentiation. Neurons born between the two dye-loading sessions incorporate both dyes and display high ratio values, whereas neurons born after the second dye loading incorporate mainly the second dye and exhibit low ratio values. Mapping these ratios across the OT closely matches the topographic organization shown in Fig. 1b: with higher ratios concentrated centrally and lower ratios in peripheral regions (Fig. 4c, left, and Supplementary Fig. 8). Calcium traces were then ordered according to dye ratio (Fig. 4c, right; *N* = 5 fish), and the correlation between neuronal activity and birthdate proxy was quantified using Spearman correlation analysis (Fig. 4d; Methods). Consistent with the macro-level analysis, early-born neurons exhibited higher visually evoked activity (Fig. 4d, red sections). Overall, early-born neurons showed a higher likelihood of activation, as indicated by a greater fraction of responsive cells across all the stimuli (*P < 0.05, one-way analysis of variance (ANOVA); Extended Data Fig. 6c). Restricting analyses to cells within fixed tectal subregions confirmed that these differences were independent of retinotopic location (Supplementary Fig. 9).

We then examined the relationship between neuronal maturation and direction selectivity (DS), by analyzing PVNs’ responses to gratings moving in eight orientations (Methods). We found a correlation between the age of individual neurons, their topographic organization and their directional tuning properties (*P* < 0.001, *R*^2^ = 0.05). Specifically, early-born neurons exhibited stronger and more consistent DS to gratings (Extended Data Fig. 6d,e), in accordance with previous studies showing that DS emerges progressively during development^12,40,41^. We confirmed these observations by tracking the visual responses of neuronal subpopulations born at defined developmental stages (Fig. 4e), using single-dye labeling to isolate neurons born before a time point, or single-pulse (two-dyes) labeling to isolate those born between two time points. Calcium imaging in the following days revealed the progressive maturation of visually evoked activity within these birthdate-defined populations (Fig. 4e).

While PVNs exhibit a clear relationship between birthdate and topographical organization, this is not generally the case throughout the nervous system. CLOK can establish this birthdate–function relationship even in spatially intermixed populations. To demonstrate this, we analyzed tectal neuropil interneurons, including superficial interneurons (SINs) and deep interneurons (DINs), whose positions do not reliably predict developmental age^42,43^. Using the same dual-dye ratio approach applied to PVNs, we were able to sort neuropil interneurons according to their birthdate and investigate the birthdate dependence of their visual responses (Fig. 4f,g). Although no functional differences were observed—even when SIN and DIN subpopulations were analyzed separately (Fig. 4g and Supplementary Fig. 10)—this experiment illustrates the ability of CLOK to separate and analyze neurons of different birthdates even when they are spatially intermingled.

Together, these experiments establish CLOK as a powerful tool for investigating the functional integration of neurons in the developing OT, enabling birthdate-based analysis of neuronal activity and the tracking of the maturation of visual responses over time.

### CLOK enables longitudinal tracking of neuronal age groups during functional maturation of the hindbrain

**Fig. 5:**
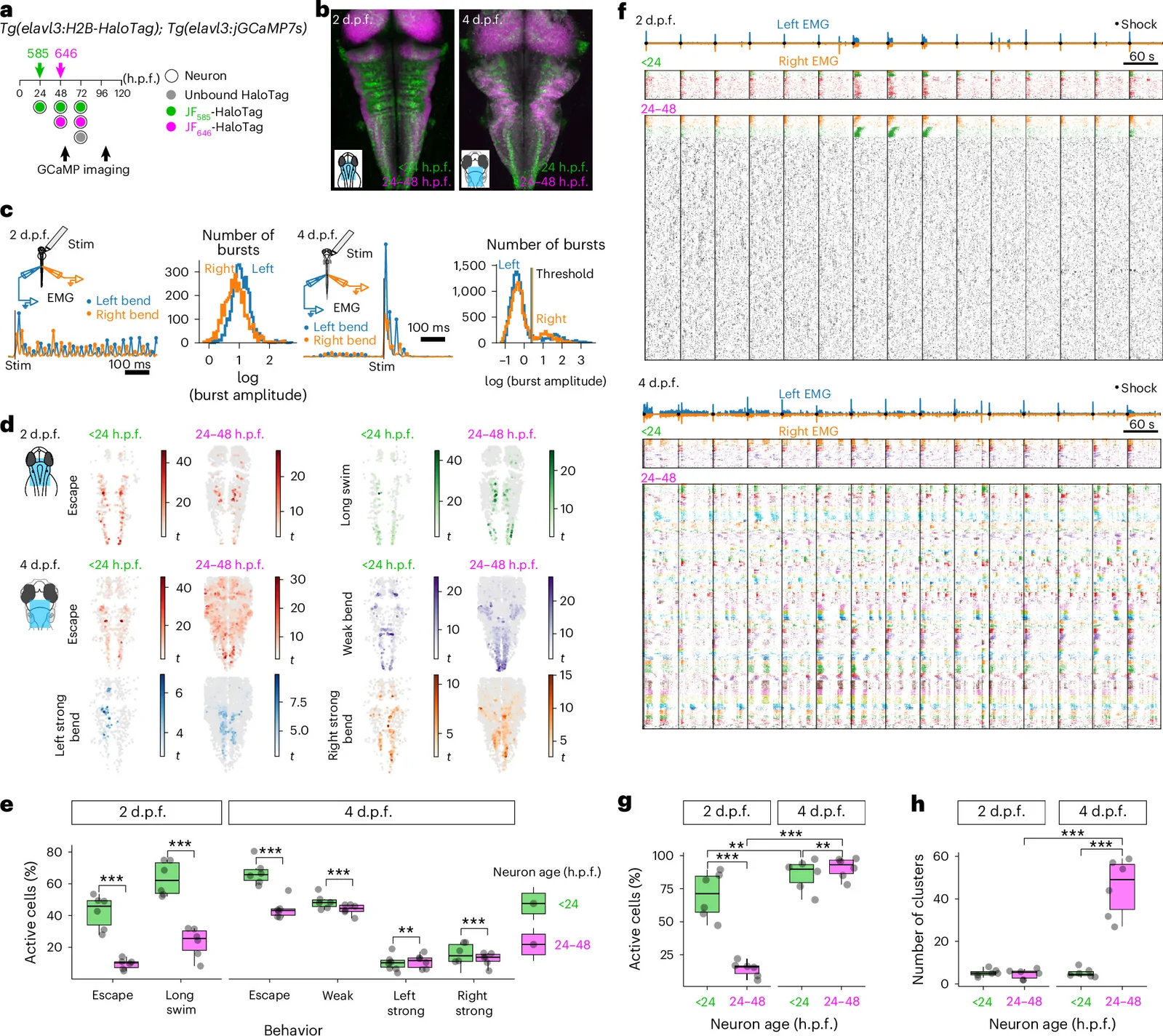
Visualization of functional maturation of neuronal age groups in the hindbrain by CLOK. **a**, Nuclei of neurons born before 24 h.p.f. and those born from 24 to 48 h.p.f. were visualized by JF_585_ and JF_646_, respectively. jGCaMP7s was expressed pan-neuronally. **b**, Visualization of the neuron group born before 24 h.p.f. (<24 h.p.f., green) and that born from 24 to 48 h.p.f. (24–48 h.p.f., magenta) in 2 d.p.f. fish (left) and 4 d.p.f. fish (right). **c**, Experimental setup for monitoring fictive locomotion during GCaMP imaging in 2-d.p.f. and 4-d.p.f. zebrafish. An electrical stimulus was delivered to the right-hand side of the head while monitoring muscle activity from both sides with EMG recording pipettes. Convolved EMG signals from left (blue) and right (orange) axial muscles during a stimulus-evoked escape episode at 2 d.p.f. and those during a spontaneous swim episode and a stimulus-evoked escape episode at 4 d.p.f. EMG peaks corresponding to left and right bends were annotated with circles. Histogram showing the distribution of the EMG amplitude per bend for left (blue) and right (orange) bends. Vertical lines in the histogram for 4-d.p.f. zebrafish indicate the thresholds to be considered ‘strong bend’ in **d**. **d**, Locomotor-related neuronal activity in each neuronal age group at 2 and 4 d.p.f. Neurons active during distinct locomotor patterns are identified on the basis of regression analysis for each neuronal age group (left, <24 h.p.f. neurons; right, 24–48 h.p.f. neurons). Neurons meeting the significance threshold (*P*_FDR_ < 0.05) are color coded based on their *t*-values. Neurons below statistical significance are visualized in gray circles. Escape, stimulus-evoked escape; left/right strong bend, left/right strong EMG bursts; long swim, sustained swim after the escape; spontaneous swim, spontaneous swims with weak EMG bursts. **e**, Percentage of neurons recruited during locomotor behaviors within each age group. Dots represent individual fish (six fish per developmental stage). Box plots show the median (center line), the 25th and 75th percentiles (lower and upper hinges of the box) and whiskers extending to the most extreme data point within 1.5× the interquartile range of the hinge and are sorted on the basis of developmental time points (2-d.p.f. and 4-d.p.f. fish) and neuronal age groups (<24-h.p.f. neurons and 24–48-h.p.f. neurons). ***P < 0.001, **P < 0.01, Bonferroni post hoc test (2 d.p.f., escape, *z* = 112.5; 2 d.p.f., long swim, *z* = 108.3; 4 d.p.f., escape, *z* = 65.0; 4 d.p.f., weak, *z* = 15.2; 4 d.p.f., left strong, *z* = 2.7; right strong, *z* = 15.6). **f**, Neuronal activity of early-born and late-born neurons during the locomotion assay at 2 and 4 d.p.f. zebrafish. Locomotor activity in response to repeated escape-inducing electrical shocks is shown at the top. Blue trace, left EMG signal; orange trace, inverted right EMG signal; black circle, electrical stimulus. Electrical artifacts from the stimulus are removed (Methods). Neuronal activity of early-born neurons and late-born populations are shown separately. Each row represents *z*-scored activity of a superneuron defined by Rastermap. Active superneurons are color coded on the basis of hierarchical clustering (Methods). **g**, Percentage of active neurons within each neuronal age group in 2- and 4-d.p.f. fish. Box plots are prepared as in **e**. Dots represent individual fish (six fish per developmental stage). ***P < 0.001, **P < 0.01, Holm post hoc test (2 d.p.f., <24 versus 24–48 neurons, *z* = 2.2; <24 neurons, 2 versus 4 d.p.f., *z* = 3.1; 24–48 neurons, 2 versus 4 d.p.f., *z* = 10.9; 4 d.p.f., <24 versus 24–48 neurons, *z* = 2.6). **h**, Number of functional clusters in each neuronal age group in 2- and 4-d.p.f. fish. Box plots are prepared as in **e**. Dots represent individual fish (six larvae per developmental stage). ***P < 0.001, **P < 0.01, Holm post hoc test (24–48 neurons, 2 versus 4 d.p.f., *t* = 10.1; 4 d.p.f., <24 versus 24–48 neurons, *t* = 10.9). Source data

To link the maturation of newborn neurons to behavioral development, we next focused on the hindbrain, which plays a critical role in the emergence of locomotor behavior^10^. We birthdated neurons born before 24 h.p.f. and those born between 24 and 48 h.p.f., and assessed their activity using 2P Ca^2+^ imaging at 2 and 4 d.p.f. (Fig. 5a, *N* = 6 fish per timepoint; Methods). The hindbrain structure changes markedly from 2 to 4 d.p.f., not just in size but also in the relative positions of the two neuronal age cohorts (Fig. 5b), highlighting the importance of visualizing both age groups in individual fish.

Consistent with previous reports^10,44^, we observed significant changes in the repertoire of locomotor behaviors measured by electromyogram (EMG). Specifically, zebrafish at 2 d.p.f. escaped in response to electrical stimuli but rarely swam spontaneously (Fig. 5c, 2 d.p.f., and Fig. 5f, 2 d.p.f., EMG traces). Muscle activity also showed a unimodal distribution (Fig. 5c, 2 d.p.f.), suggesting that zebrafish at this stage produce a limited range of muscle activity. By contrast, 4 d.p.f. larvae showed robust spontaneous swims in addition to escape responses evoked by electrical stimuli (Fig. 5c, 4 d.p.f., and Fig. 5f, 4 d.p.f., EMG traces). Muscle activity during spontaneous swims was also distinctively weaker (Fig. 5c, 4 d.p.f.), resulting in a broader range of muscle activity (Fig. 5c, 4 d.p.f.).

To assess how the activity of each neuronal age group changes during this behavioral development, we performed regression analysis to identify correlated activity with distinct locomotor events. In 2-d.p.f. larvae, we looked for neurons whose activity correlated with escapes or long swims, on the basis of the variability of the length of escape episodes at this stage (Fig. 5f, 2 d.p.f., EMG traces), and found that both functional types are present in each neuronal age group (Fig. 5d, 2 d.p.f.). However, a much smaller proportion of the late-born group showed activity related to these locomotor events (Fig. 5e, 2 d.p.f., *P* < 0.0001, corrected for multiple comparisons, using a generalized linear mixed model for binomial distribution with subjects and locomotor types as random effects), suggesting that the late-born group is still functionally immature. In 4-d.p.f. larvae, we looked for neurons whose activity correlated not only with escape but also with locomotor events not present at 2 d.p.f., including spontaneous swim and strong lateralized responses during spontaneous swim. As in 2-d.p.f. larvae, each functional class was present in both neuronal age groups. However, the proportion of active neurons in each age group showed a significant interaction between subject age and neuronal age (*P* < 2 × 10^−16^) (Fig. 5e), suggesting ongoing functional maturation of the late-born group.

**Extended Data Fig. 7:**
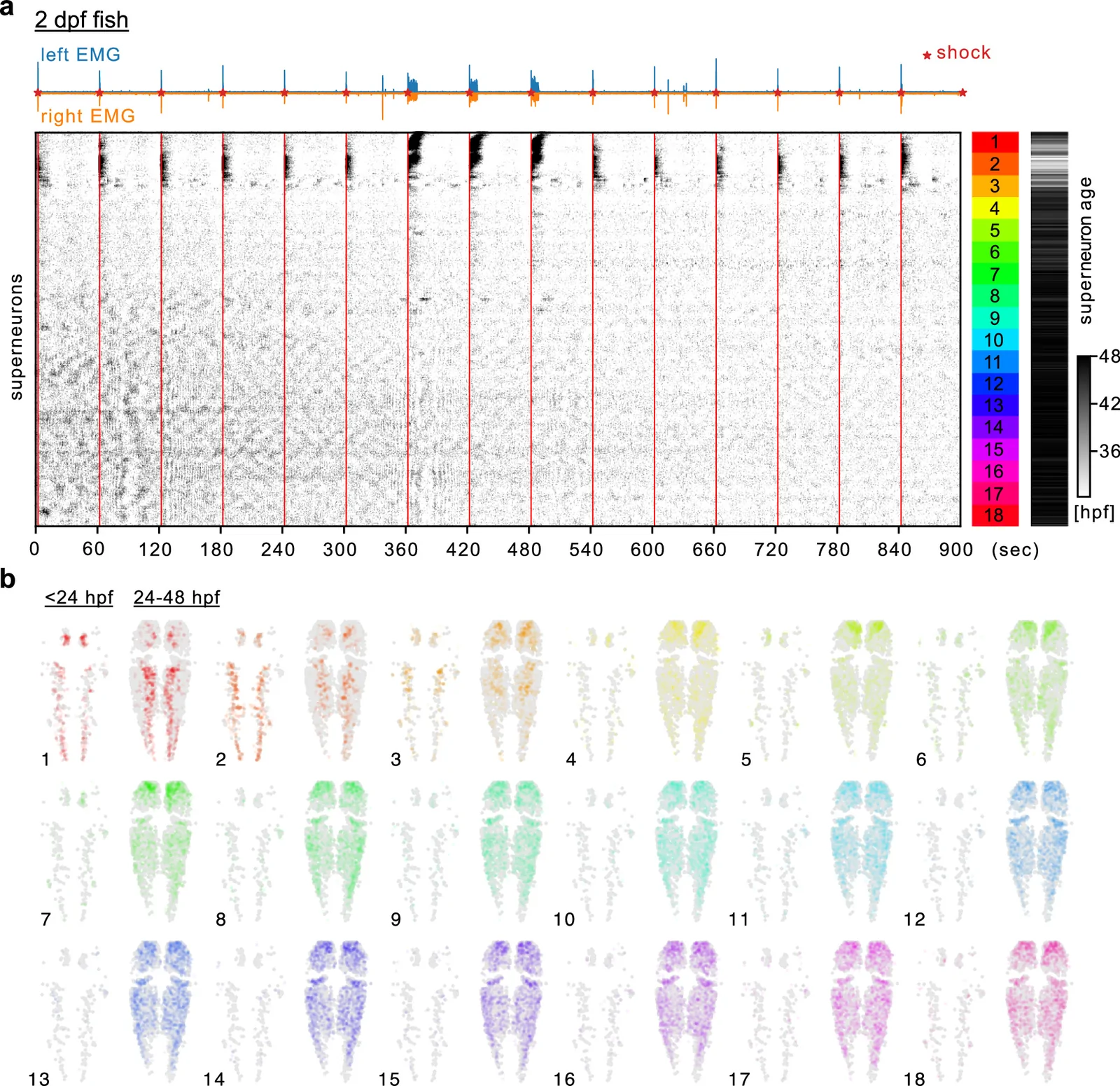
Representative sorting of neuronal activity during escape assay in a 2 dpf zebrafish. **a**, Top: Locomotor activity in response to repeated escape-inducing electrical shocks. Blue trace, Left EMG signal; Orange trace, inverted right EMG signal; Red circle, electrical stimulus. Electrical artifacts from the stimulus are removed (see Material and Methods). Bottom: Neuronal activity sorted by Rastermap. Each row represents z-scored activity of superneurons. The average neuron age in each superneuron is represented in gray scale. **b**, Maps of functional groups colored by position in Rastermap sorting. Each functional group is further divided into neurons born by 24 hpf and those born between 24 and 48 hpf.

**Extended Data Fig. 8:**
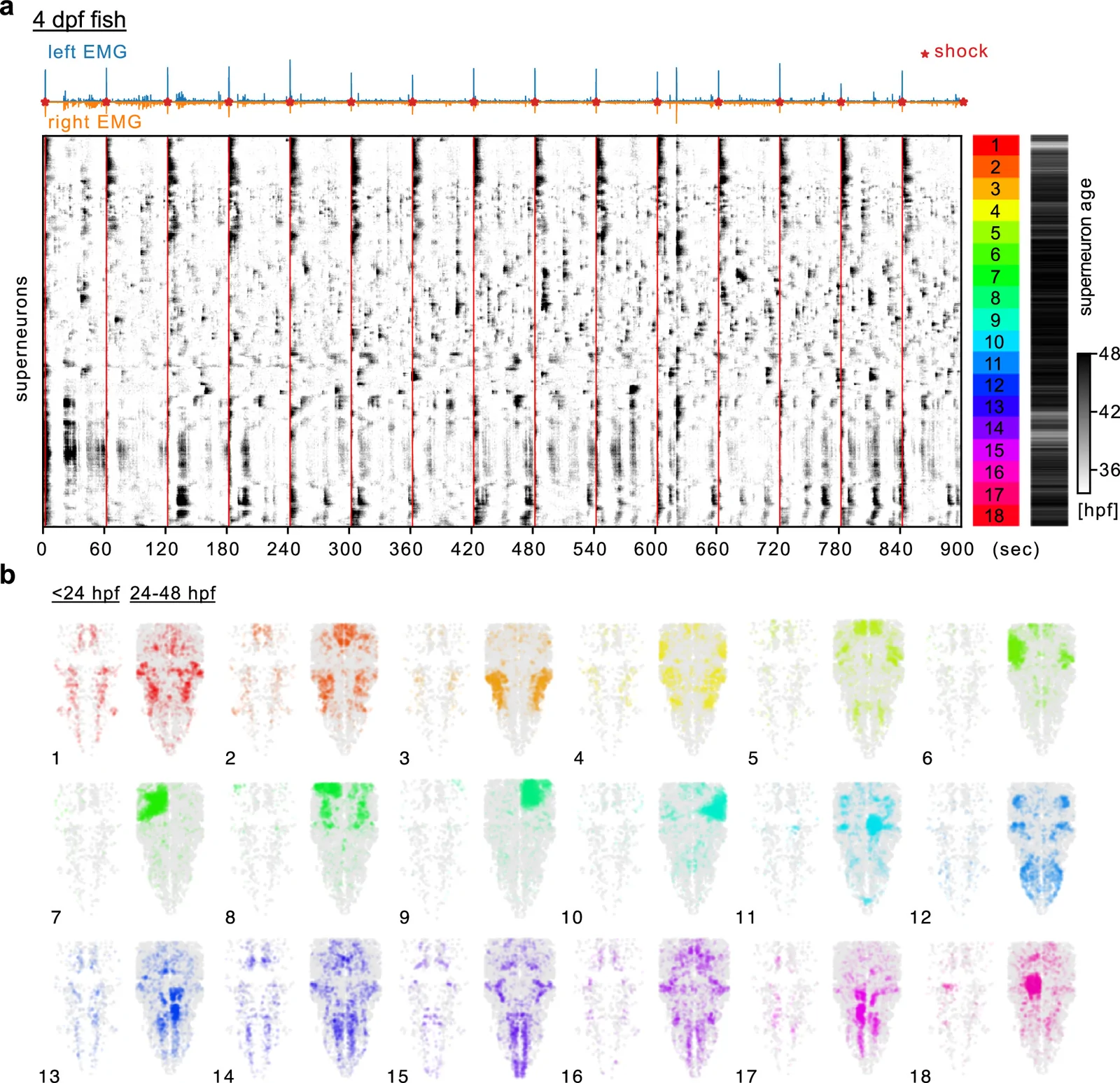
Representative sorting of neuronal activity during escape assay in a 4 dpf zebrafish. **a**, Top: Locomotor activity in response to repeated escape-inducing electrical shocks. Blue trace, Left EMG signal; Orange trace, inverted right EMG signal; Red circle, electrical stimulus. Electrical artifacts from the stimulus are removed (see Material and Methods). Bottom: Neuronal activity sorted by Rastermap. Each row represents z-scored activity of superneurons. The average neuron age in each superneuron is represented in gray scale. **b**, Maps of functional groups colored by position in Rastermap sorting. Each functional group is further divided into neurons born by 24 hpf and those born between 24 and 48 hpf.

To examine whether this gradual maturation of the late-born group also applies to functional classes besides those involved in locomotion, we examined the spontaneous activity of both neuronal age groups at 2 and 4 d.p.f. and determined the proportion of active neurons within each group and the diversity of their activity (Fig. 5f–h and Extended Data Figs. 7 and 8; Methods). At 2 d.p.f., the majority of the early-born group was active (Fig. 5f,g, 2 d.p.f., <24-h.p.f. neurons) while only a minority of the late-born group was active (Fig. 5f,g, 2 d.p.f., 24–48-h.p.f. neurons). At 4 d.p.f., the proportion of active neurons within the late-born population increased (Fig. 5f,g; *P* < 0.001). Furthermore, the functional diversity of the late-born population increased from 2 d.p.f. (Fig. 5f,h; *P* < 0.001) and became greater than that of the early-born population (Fig. 5h; *P* < 0.001). Activity maps of sorted neuronal activity indicate that the neurons not directly related to locomotion, including those in the tectum, contribute to this diversity (Extended Data Fig. 8).

These observations support the idea that the gradual maturation of the late-born group applies generally across the examined brain regions. Interestingly, the analysis of locomotor-related activity revealed that early-born neurons were recruited into behaviors absent at 2 d.p.f. (weak swim: 48.2% (median), left strong bend: 10.3% (median), right strong bend: 14.7% (median); Fig. 5d,e, 4 d.p.f.). This raises the possibility that coordination between neuronal age groups emerges during this period and may play a critical role in the development of motor behavior.

Taken together, we showed that CLOK enables longitudinal activity tracking of multiple neuronal age cohorts in a developing vertebrate and opens the avenue to investigating how their coordination contributes to behavioral maturation.

### Integrating CLOK with sensors and actuators for birthdate-selective functional imaging and photoactivation

**Fig. 6:**
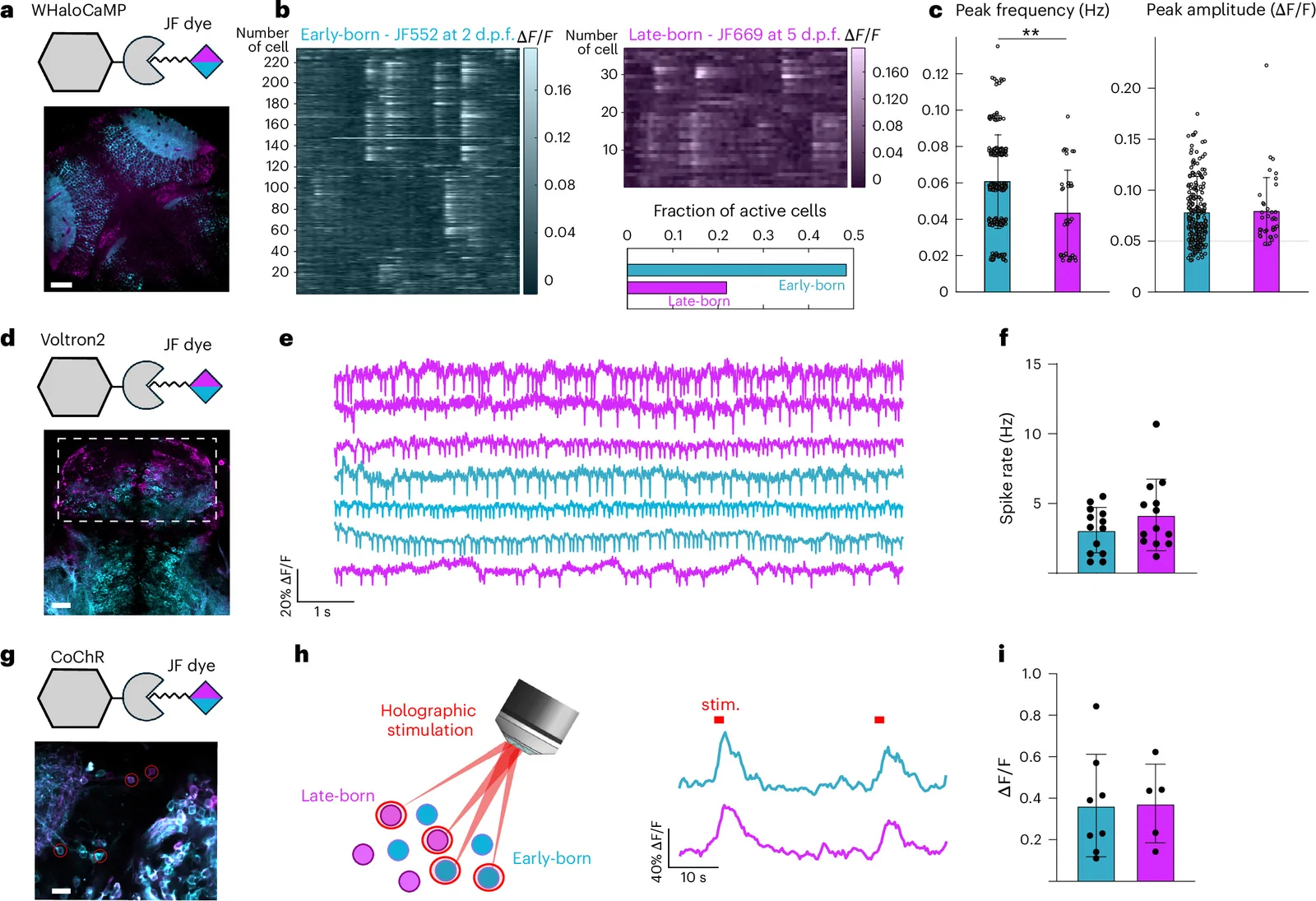
CLOK-based birthdate-selective tagging of optogenetic constructs. **a**, CLOK applied to WHaloCaMP for birthdate-resolved calcium imaging. Schematic of WHaloCaMP tagged by JF dyes and corresponding dual dye image of the OT from a *Tg(elavl3:WHaloCaMP-eGFP)* larva (JF_552_ incubated at 3 d.p.f., JF_669_ at 5 d.p.f.). Scale bar, 50 μm. **b**, Raster plot of Δ*F*/*F* traces from the active cells from the two birth-selected populations, early- and late-born (cyan and magenta, respectively). Recordings from the two populations were performed sequentially with two different excitation wavelengths (561 nm and 642 nm, respectively). Bottom right: a visualization of the fraction of active cells for each subpopulation (233 out of 480 active cells for early-born population, 37 out of 164 for the late-born one). **c**, Bar and scatter plots of the peak frequency and peak amplitude extracted from traces of individual active cells of early- and late-born neurons (cyan and magenta, respectively). A small random offset is applied in both *x* and *y* directions to avoid overlapping dots. Bars represent mean ± s.d. (*n* = 233 and 37 cells for early- and late-born population respectively; *N* = 1 fish). **P < 0.01, Kolmogorov–Smirnov test. **d**, CLOK applied to Voltron2 for birthdate-resolved voltage imaging. Schematic of Voltron2 tagged by JF dyes and corresponding dual dye image of the forebrain from a larva transiently expressing QUAS:voltron2 under pan-neuronal promoter (region delimited by white dotted rectangle; JF_525_ incubated at 3 d.p.f., JF_585_ at 5 d.p.f.). Scale bar, 25 µm. **e**, Examples of Voltron2 fluorescence transients from the two birth-selected populations (early- and late-born) acquired in sequential trials under dual laser light-sheet imaging. **f**, Bar plots of the spiking rate from early and late-born neurons, cyan and magenta, respectively (mean ± s.d., *n* = 13 cells per population, *N* = 3 fish; *P* > 0.05, two-sided Student *t*-test). **g**, CLOK applied to CoChR opsin for birthdate-resolved optogenetic stimulation. Schematic of CoChR opsin tagged by JF dyes and corresponding dual dye image of a CoChR-expressing larva (JF_552_ incubated at 3 d.p.f, JF_669_ at 5 d.p.f.). Scale bar, 20 µm. **h**, In vivo functional validation of birth-selective optogenetic stimulation of tectal neurons co-expressing CoChR-HaloTag (transient expression of Xla.Tubb2-hsp70-ubc:CoChR-HaloTag) and GCaMP6s (*Tg(elavl3:H2B-GCaMP6s*). Left: after dual color birthdate-based labeling, early-born (cyan) and late-born (magenta) neurons were selected for selective holographic photostimulation. Right: exemplary response traces from an early-born (cyan) and a late-born neuron (magenta) showing calcium increases corresponding to photostimulation pulses (red ticks). **i**, Bar plot of the photostimulation-induced Δ*F*/*F* peak amplitude from early-born and late-born populations (mean ± s.d., n_552_ = 8 cells, n_669_ = 5 cells; *N* = 3 fish; *P* > 0.05, two-sided Student’s *t*-test). Source data

We next designed proof-of-principle experiments to demonstrate that CLOK can be coupled with multiple chemigenetic optical tools for versatile age-related optical investigations (Fig. 6). We first validated this strategy using WHaloCaMP, a chemigenetic calcium indicator in which the calcium-sensitive domain is fused to HaloTag^45^. Two-dye CLOK labeling in WHaloCaMP-expressing larvae (JF_552_ at 3 d.p.f. and JF_669_ at 5 d.p.f.), followed by sequential functional imaging of each dye, enabled selective monitoring of spontaneous activity in neurons of distinct birthdates (Fig. 6a–c and Supplementary Fig. 11). Clustering early- and late-born neurons according to fluorescent labeling (Methods), we found a higher frequency of spontaneous events in the early-born population, consistent with previous studies^13^. A similar strategy was applied to the rhodopsin-based voltage indicator Voltron2, which combines a microbial rhodopsin voltage sensor with a HaloTag-JF dye fluorophore^46^. Given the absorption spectrum of its rhodopsin domain, we loaded JF_525_ and JF_585_ at 3 and 5 d.p.f. to record voltage signals from two distinct neuronal age groups. Fast fluorescent transients were recorded in the forebrain and the olfactory epithelium, a region with reliable spontaneous spiking^46^. These results demonstrate the detection of action potentials and extraction of firing rates in birth-resolved neuronal populations (Fig. 6d–f).

To further demonstrate CLOK’s versatility, we extended it to all-optical experiments, where opsins and activity indicators are used together to enable simultaneous optical manipulation and recording of neuronal activity^47^. To this end, we fused HaloTag to our opsin of choice, CoChR^48,49^. All-optical approaches typically benefit from spectral separation between the opsin, the activity indicator and the opsin’s fluorescent tag. First, to demonstrate an increased flexibility provided by JF dye labeling for tagging opsin-expressing cells, we characterized imaging-induced opsin activation and evaluated the cross-talk between dye imaging and CoChR: in Chinese hamster ovary (CHO) cells expressing CoChR-HaloTag, we recorded photocurrents during 2P photostimulation and simultaneous imaging at different wavelengths (Supplementary Fig. 12). The orthogonality between CoChR excitation spectrum^50^ and the distinctive 2P absorption profile of JF dyes (here, JF_585_; peaks at 800 nm and >1,000 nm) allowed bright imaging and identification of CoChR-expressing cells with minimal artifactual photocurrents from the imaging laser. By contrast, GFP-tagged CoChR, which requires imaging at ~950 nm, induced substantially larger artifactual photocurrents (>200 pA), potentially causing unwanted depolarization during cell screening. We next tested this construct in vivo by transiently expressing CoChR in *Tg(elavl3:H2B-GCaMP6s*) and performing simultaneous 2P imaging and 2P holographic photoactivation of user-defined cell groups. Opsin-expressing neurons were identified by two-dye CLOK labeling (Fig. 6g; JF_552_ at 3 d.p.f. and JF_669_ at 5 d.p.f.), and because of the spectral selectivity in both excitation and emission, HaloTag labeling could be readily separated. Using 2P holographic stimulation, we selectively targeted either early- or late-born neurons (Fig. 6h, left) while recording GCaMP responses across the full field of view (FOV) at reduced laser power to limit imaging-induced activation (Methods). Photostimulation-evoked calcium responses were clearly detected in stimulated neurons (Fig. 6h, right), confirming successful activation. Mean fluorescence changes (Δ*F*/*F*) were similar between populations (early-born: 0.20 ± 0.02; late-born: 0.22 ± 0.02, *P* = 0.94, Student’s *t*-test; Fig. 6i), demonstrating the capability to stimulate either early- or late-born neurons at will and with equal efficiency.

Altogether, this proof-of-principle set of experiments demonstrates the potential of CLOK for all-optical investigation of birthdate-resolved subpopulations, opening avenues for dissecting their incorporation into developing neural circuits.

## Discussion

We developed CLOK, a neuronal birthdating strategy designed to overcome longstanding challenges in tracking age-defined populations and studying the maturation of their functional roles within neural circuits. The approach combines HaloTag technology with JF dyes to label, in a living animal, the entire population of neurons differentiating within a defined time window. Unlike traditional birthdating approaches, CLOK is compatible with a broad range of genetic and optical tools, including subcellular tagging, calcium and voltage imaging, and optogenetic manipulations, providing a powerful and versatile platform to investigate how newborn neurons wire into functional circuits and shape behavior.

Current birthdating methods each carry some drawbacks. BrdU and EdU, the most widely used markers, incorporate into newly synthesized DNA and thus label all dividing cells indiscriminately; worse, visualization requires tissue fixation, ruling out in vivo use altogether. Electroporation^51^ and heat-shock-based genetic approaches^52^ avoid tissue fixation but still mark all sibling cells across multiple rounds of division, thus requiring sparse labeling for interpretability. More sophisticated strategies that synchronize reporter switching with the cell cycle exist^18,19^ but typically yield limited numbers of labeled late-born cells. By contrast, CLOK labels entire neuronal populations at single-cell resolution in vivo, without the cell-to-cell labeling stochasticity that plagues other strategies.

The simplicity of HaloTag engineering further expands the scope of possible applications. At the anatomical level, subcellular labeling revealed where axonal and dendritic processes of each age-defined population reside across the vertebrate brain—a picture that becomes even more informative when combined with cell-type-specific targeting. On the functional side, pairing CLOK with HaloTag-based calcium and voltage indicators allowed us to read out neural activity sorted by birthdate. We also tagged opsins directly with CLOK, enabling 2P photostimulation of neurons selected by birthdate rather than by cell type or anatomy alone. Combined with recently developed HaloTag-based photoablation, similar protocols may also enable ablation of age-specific cellular populations^53^.

Using the spectral range of biocompatible JF dyes from 525 to 669 nm, we simultaneously labeled up to five cohorts in a single animal. These JF dyes are clearly separable from GFP-based indicators under both 1-photon and 2P excitation, which is especially convenient for pairing with the most commonly used GFP-based calcium and voltage indicators^54,55^. A related advantage is that HaloTag itself produces no fluorescence, so CLOK Tg lines are spectrally neutral—the experimenter is free to choose whichever dye best suits the fluorescent proteins or opsins already in the experiment, rather than being locked into the spectral identity of a genetically encoded reporter. Fully exploiting multiplexed labeling will require further refinement of dye combinations for spectral separation and tissue compatibility, but the approach already opens up questions about individual variation in neurogenesis that were previously difficult to address.

Newborn neurons in the OT, irrespective of their birthdate, exhibited more robust visual responses as they matured and became functionally similar to the early-born, pre-existing neurons. In the hindbrain, newborn neurons matured over a comparable time frame but settled into activity patterns that seem distinct from those of early-born neurons. These pre-existing neurons also change their activity as the newborns mature, suggesting potential interactions between neurons across age groups. Therefore, while the pace of maturation appears similar across regions, the endpoint differs—a distinction that CLOK is well suited to investigate further at the circuit and molecular levels.

How well CLOK resolves distinct temporal cohorts over an extended period depends on a few factors. Promoters that switch on during differentiation and then go quiet—such as *elavl3*—give sharper temporal boundaries. Those that stay active into maturity, such as *Xla.Tubb2*^56^, trade some precision for longer tracking windows. Progenitor-targeted expression is also possible but behaves more like thymidine-based methods, labeling progenitors and progeny alike; it is better suited to lineage tracing than to birthdating per se. Dye selection adds another consideration: differences in lactone–zwitterion equilibrium affect bioavailability and can shift the apparent age-group boundaries between experiments, so the same dye set should be used consistently across conditions. Protein tags are also known to affect protein turnover, influencing how long birthdated neurons can be observed. These factors can also influence dye signal ratios, which we use here to determine the age of labeled neurons by splitting them into distinct age groups or revealing the age gradient within the population labeled during a longer pulse. Treating the dye ratio as a continuous variable needs careful examination and validation, that is, by comparing the result to the discrete labeling of newborn neurons with a shorter pulse or to the standard thymidine-based birthdating methods. However, once validated, the dye-ratio approach can reveal age information without requiring a separate dye for each age group. Although precautions and systematic characterization are needed to account for how these factors influence dye signal ratios and age-related clustering, our successful demonstrations nonetheless speak to the robustness of CLOK.

We focused on zebrafish owing to the high bioavailability (that is, skin penetration) of the dye, but the core mechanism of CLOK can also be applied to other in vivo animal models such as *Caenorhabditis* *elegans*^57^, *Drosophila*^58^, mouse^46^ or in organoid systems, where it could help map neurogenic trajectories through complex 3D tissues^59^. The versatility of CLOK in terms of promoter-driven expression makes it readily adaptable to other biological contexts, such as the study of glial development and function, or hematopoietic lineage formation.

In conclusion, CLOK offers a practical, noninvasive way to combine precise birthdate labeling with the growing toolkit of HaloTag-based reagents. By bringing together single-cell resolution birthdating, functional imaging and all-optical circuit manipulation within a single framework, it provides a platform for studying neuronal integration—anatomically, physiologically and at the circuit level—that improves on existing methods in both reliability and flexibility.

## Methods

### Animals

All procedures involving zebrafish were conducted in accordance with national and European (2010/63/EU) guidelines, the animal research guidelines from the Canadian Council on Animal Care and the University of Toronto Local Animal Care Committee. They were approved by the authors’ institutional review boards and national authorities (French Ministry of Research, protocol ID: APAFIS no. 21323 and no. 49266; University of Toronto, protocol ID: 20012777). Zebrafish were obtained from an in-house breeding colony of wild-type, Tg and mutant adults maintained at 28.5 °C on a 14-h light–10-h dark cycle with sunrise and sunset. Embryos were raised in a separate incubator but at the same temperature and on the same light–dark cycle (in fish water: 0.3 g l^−1^ Instant Ocean). Fertilized embryos were collected every hour and housed in separate Petri dishes to determine the time of fertilization at the temporal resolution of hours. The housing density was maintained lower than 60 embryos per 10-cm Petri dish to ensure optimal growth. Embryos were staged and only the ones with normal development were used in the study. Juvenile fish were kept in the fish facility until the day of the experiment (transferred in beakers with fish water and kept in the incubator before imaging).

### Plasmid and Tg lines generation

We generated different HaloTag-based constructs by subcloning (Gibson Assembly Cloning Kit) HaloTag and other specific sequences and inserting them into various promoters: *p5’ QUAS*^60^; *Xla.Tubb2b-hsp70-ubc*, *elavl3* and *14UAS*. HaloTag was subcloned alone or with H2B sequence, for nuclear expression. In some cases, we used, as reporters, the zebrafish *hatching enzyme 1, tandem duplicate 1 (he1.1)* promoter^60^, driving CFP or mCherry expression in the hatching gland, or the *myosin light chain (myl7)* promoter^61^, driving the expression of GFP in the heart. The following previously published Tg lines were used: *Tg(xla.Tubb2b:QF2)*^60^, *Tg(elavl3:H2B-GCaMP6f)*^62^; *TgBAC(slc17a6:EGFP)*^63^ (hereafter referred to as *Tg(vglut2a:EGFP)*); *Tg(elavl3:Voltron-ST)*^64^, the last two of which were propagated to the nacre background. The Tg lines under the *14UAS* promoter were generated using the Tol1 transposon system, while the ones under the *Xla.Tubb2-hsp70-ubc* promoter were generated using the Tol2 transposon system. The following Tg lines were generated using the Tol2 transposon system: *Tg(elavl3:H2B-HaloTag)*, *Tg(elavl3:msyp-HaloTag)*, with plasmids generated by cloning the corresponding reporters to pTol2-elavl3:Voltron-ST^64^. H2B-HaloTag and msyp-HaloTag were subcloned from piggybac-EF1-Halo-H2B and piggybac-hsyn-msyp-Halo-neo, respectively (both of which are generous gifts from Dr. James Zhe Liu). *Tg(elavl3:H2B-HaloTag)* and *Tg(elavl3:jGCaMP7s)* were propagated to the cacnb1 ts25/+ mutant background^65^ and maintained as heterozygous mutants. The WHaloCaMP plasmid was taken from^45^ and the Voltron2 sequence from^46^ (generous gift from Dr Takashi Kawashima) and subcloned in the QUAS promoter expressing he1.1:CFP.

Experiments were performed on stable Tg lines or in embryos transiently expressing the plasmids, as specified in the main text or figure legends (Figs.1–6, Extended Data Figs. 1–8 and Supplementary Figs. 1–14). The DNA constructs (25 ng µl^−1^) were co-injected with tol1 (for *14UAS* promoter) or tol2 (for *Xla.Tubb2:QF2-hsp70-ubc* and *elavl3* promoters) transposase mRNA (25 ng µl^−1^) at the one-cell stage in Casper mutant zebrafish embryos. Founders were identified by screening progeny for fluorescence in the hatching gland or the heart or for JF dye fluorescence directly. The different generated plasmids and stable lines are presented in Supplementary Table 1.

### JF dye incubation and preparation of zebrafish larvae

To label HaloTag-expressing neurons, the embryos were incubated in a solution containing the fluorescent HaloTag ligand JF dye (3.3 μM JF dye and 0.033 % dimethyl sulfoxide (DMSO), unless otherwise specified; see below) in fish rearing water at room temperature for 2 h. Following incubation, larvae were rinsed three times for 10 min in fish rearing water to remove excess dye. The incubation was performed at different stages of larval maturation (indicated in terms of hours post fertilization (h.p.f.) or days post fertilization (d.p.f.)). After screening for the fluorescence of the JF dye in the brain, the fish were returned to fish rearing water in the incubator until the time of the experiment. JF dyes can be obtained via the HHMI’s Open Chemistry initiative (https://dyes.janelia.org/) or purchased from Promega Corporation.

### Quantification of the dye washout

The 4-d.p.f. larvae were incubated in a solution containing a single HaloTag ligand dye (JF_525_ or JF_635_ or JF_669_) for 2 h at room temperature. After incubation, excess dye was removed with a 3× 10 min rinse (as done for all the experiments), and larvae were immediately transferred to fresh embryo medium. Confocal images were acquired at multiple time points over the following 24 h (30 min, 2 h, 4 h and 24 h) to monitor the clearance of unbound dye. Fluorescence intensity was quantified in HaloTag-expressing neuronal somata within a defined brain region and compared with background fluorescence in adjacent neuropil regions lacking HaloTag expression. Signal-to-background and background-to-signal ratios were computed for each dye and time point to characterize the dynamics of nonspecific fluorescence decay.

### Swim bladder development and body length measurements following dye loading

To assess any developmental delays induced by the loading of the dyes, we performed two sets of control experiments in WT larvae: (1) two dyes (JF_585_ and JF_635_) were loaded in sequence at 1 and 2 d.p.f.; (2) a single dye (JF_552_) was loaded at 2 d.p.f. These groups were compared with DMSO-treated and control siblings. We measured three parameters: (1) the survival rate was calculated from 3 to 5 d.p.f.; (2) the percentage of larvae with an inflated swim bladder was calculated from 3 to 5 d.p.f.; and (3) the length of the larvae (anesthetized with tricaine (160 mg l^−1^) and imaged with a stereomicroscope) was measured at 5 d.p.f.

### Comparison of HaloTag birthdating and EdU labeling

#### Single-pulse HaloTag birthdating

Two JF dyes were loaded with a 24-h separation in the Tg line expressing HaloTag as well as GCaMP6f in the nuclei of differentiating neurons with the *elavl3* promoter to distinguish neurons born before the first dye loading from those born between the first and second dye loading. JF_635_ was chosen as the second dye owing to its fluorogenic property. This allowed us to minimize the signal from unbound JF-dye molecules when we image fish right after the second dye loading. JF_585_ was chosen as the first dye on the basis of its brightness and clear separation from the fluorescence signals from GCaMP6f and JF_635_. The dye loading procedure was based on ref. ^26^ with a few modifications. The 0.3-µM dye solution was prepared by dissolving 1.67 µl of 1-mM DMSO stock solution in 5 ml system water. Fish were incubated in the 0.3-µM solution for 2 h and then rinsed thoroughly with system water before transferring them to a new 10-cm culture dish filled with clean system water. Embryos before hatching were dechorionated to facilitate the washout of the unbound dye molecules from embryos. Five neuronal age groups were targeted in this experiment: group 1, neurons born before 24 h.p.f.; group 2, neurons born from 24 to 48 h.p.f.; group 3, neurons born from 48 to 72 h.p.f.; group 4, neurons born from 72 to 96 h.p.f.; and group 5, neurons born from 96 to 120 h.p.f. All groups were imaged between 122 and 126 h.p.f. using a confocal microscope (Zeiss, 710/800). Fish were anesthetized in tricaine methanesulfonate (MilliporeSigma, E10521) dissolved in system water at 160 mg l^−1^ (hereafter referred to as MS-222) and then embedded in 1.6% low-melting-point (LMP) agar (MilliporeSigma, 2070-OP). Volumetric images of the rostral and caudal halves of the brain were imaged separately with a 20 × 1.0 numerical aperture (NA) objective lens (Zeiss, W Plan-Apochromat 20×/1.0) and then stitched together with the stitching plugin available through Fiji^66^.

#### EdU labeling

EdU labeling was performed using a Click-iT EdU Alexa Fluor 647 Imaging Kit (ThermoFisher, C10340) with modifications. Fertilized eggs from *Tg(elavl3:H2B-GCaMP6f)* were mounted on the basis of developmental stages. Embryos at 4 h.p.f. were placed on the standard egg injection mold used for single-cell DNA injection. Embryos at 24 h.p.f. were dechorionated and embedded in 0.6% LMP agar. Zebrafish at 48 h.p.f. and onward were embedded in 1.2% LMP agar. Then a 0.8-nl bolus of 10 mM EdU solution was pressure injected into the yolk via a glass micropipette connected to a microinjector (Eppendorf, FemtoJet). Assuming all the EdU molecules were retained in the body, this translates to less than 5 ng µl^−1^ of EdU concentration in the body, which is less than the bath concentration of thymidine analogs used in past zebrafish studies^67^. Zebrafish were transferred back to a new culture dish filled with clean system water and kept in an incubator until 120 h.p.f. when they were killed by an overdose of 0.04% MS-222, then immediately fixed overnight in 4% paraformaldehyde in 0.1 M phosphate buffer (pH 7.4) at 4 °C. Following 1× PBS rinses, fixed larvae were pinned down through the notochord to a Sylgard-coated (Dow Corning) platform filled with PBS, and the central nervous system was carefully excised using fine forceps and custom-etched tungsten dissecting pins. The subsequent staining procedure follows the procedure described in the kit, except the fish were kept in the cocktail without the buffer additive at −20 °C for an hour before starting the reaction with the buffer additive at room temperature. This additional step minimized the signal amplification biased toward the brain’s surface. The stained brains were imaged under a custom 2P microscope to obtain homogeneous signal across the entire depth of the brain for both GCaMP and Alexa Fluor 647, which were excited with a femtosecond laser tuned to 830 nm (MKS Instruments, Mai Tai HP, Deep See). The entire brain was imaged at once using a 16× objective lens (Nikon Instruments, CFI75 LWD 16× W).

### Single-pulse HaloTag birthdating of subcellular structures

To visualize presynaptic terminals of neuronal age groups, we used *Tg(elavl3:msyp-HaloTag)*, the elavl3 Tg line expressing HaloTag with synaptophysin localization sequence^36,68^. To emphasize the signal from dendritic processes, we chose the elavl3 Tg line expressing HaloTag-based voltage indicator with Kv2.1 localization sequence because this sequence has been shown to target the tagged protein to somatodendritic processes^37,69,70^. They were subjected to the same single-pulse labeling procedure based on two JF dyes, JF_585_ and JF_635_, as described above, and the data were processed similarly. To ensure precise registration of subcellular structures, these Tg lines were crossed with *TgBAC(vglut2a:GFP)*, which exhibits GFP signal broadly in the neuropil area. The whole-brain volumes were acquired using the same imaging and stitching protocol as described in Single-pulse HaloTag birthdating.

### Multipulse HaloTag birthdating

To visualize all five age groups examined above in the same animal, a series of JF dyes was loaded onto the developing *Tg(elavl3:H2B-HaloTag)* in 24-h intervals: JF 525 (JF_525_) at 24 h.p.f., JF_585_ at 48 h.p.f., JF_669_ at 72 h.p.f., JF_549_ at 96 h.p.f. and JF_635_ at 120 h.p.f. The dye loading protocol is identical to the one described above. However, based on the observation that neuronal age groups at similar age are also close in space, the order of the dyes was chosen to maintain reasonable separation of excitation and emission spectra across neighboring age groups. We also observed in preliminary experiments that the loading of JF_525_ at a later developmental time point resulted in the labeling of neurons near the blood vessels, leading us to use it as the first dye. JF_635_ was chosen as the last dye owing to its fluorogenic property as described above. The imaging procedure is also similar to the one described above except that we used an inverted confocal microscope equipped with white-light laser (Leica, Steralis) to excite each of the five dyes at the optimal wavelength. We compared the sequential multilabeling using both configurations (white laser or standard lasers) and observed similar topographical patterns (Supplementary Fig. 13). Fish were mounted in 1.6% LMP agar ventral-side up and imaged with a 25× 0.95 NA objective lens (Leica, HC FLUOTAR L 25×/0.95 W VISIR).

### Optical systems

#### Confocal microscope

The five-dye multipulse birthdating images in Fig. 2 were acquired with a Leica Steralis system equipped with a white-light laser. JF_525_, JF_549_, JF_585_, JF_635_ and JF_669_ were excited at 525, 549, 585, 635 and 669 nm, respectively. Fluorescent images shown in Fig. 3 and Supplementary Fig. 14 were acquired using a commercial confocal microscope (Olympus FV3000). Multiple laser sources (488, 514, 561, 594 and 640 nm) and customizable wavelength detection windows (based on spatial dispersion of the signal and a multidetector array) were available, enabling the selective excitation and detection of individual JF dyes. The two-dye single-pulse birthdating images shown in Figs. 1 and 2 were acquired using Zeiss 710 or 800. GCaMP/GFP, JF_585_ and JF_635_ were excited with 488, 561 and 640-nm lasers, respectively.

#### 2P imaging

2P imaging was performed using two different 2P scanning microscopes. For Fig. 4, we used a commercial model (LAVision, Miltenyi Biotec) combined with a tunable Ti:Sapphire femtosecond laser (Coherent Chameleon, wavelength range 700–1100 nm) and visual stimulation. JF dye imaging was typically performed at 800–850 nm, using dedicated band-pass filters in front of the photomultipliers (PMT) (typically a 525/50 for the green PMT and 595/40 or 700/40 (exchangeable) for the red PMT) to minimize cross-talk in the presence of multiple dyes and to avoid contamination of the GCaMP recordings. Calcium transients were acquired using galvo–galvo bidirectional 2P scanning at 3 Hz at 920 nm with an FOV of 300 × 300 μm. A slightly different configuration was used for imaging EdU-labeled fixed samples and functional recordings during locomotion behavior (Fig. 5), consisting of a custom microscope equipped with a resonant scanner (Thorlabs, MPM-2PKIT), a piezo objective scanner (PI, P-725K129) and a 16× objective lens (Nikon Instruments, CFI75 LWD 16x W) controlled by ScanImage (Vidrio Technologies). Of note, the unique 2P excitation spectrum of JF dyes, characterized by a double peak with minimal absorption at ~920 nm (ref. ^27^), makes them ideal for pairing with GFP-based calcium indicators. We confirmed this feature by assessing that the presence of the dye did not interfere with the detection of spontaneous calcium transients. Typically, the dye leakage into the PMT used for GCaMP detection was below 5%, indicating a negligible contribution to functional recordings (JF_525_: 27.5%, JF_552_: 0%, JF_585_: 4.7%, JF_669_: 0.21%; Supplementary Fig. 14).

For the imaging of EdU-labeled samples, GCaMP6f and Alexa Fluor 647 were excited with a femtosecond laser tuned to 830 nm (MKS Instruments, Mai Tai HP, Deep See). During the functional imaging, all the fluorophores were excited with a femtosecond laser tuned to 940 nm (MKS Instruments, Mai Tai HP, Deep See) to prioritize the functional GCaMP signal. Multichannel functional volumes (512 × 256, 30 slices, 7-µm *z* step) were acquired for a period of 30 min at a volume rate of 2 Hz. Volumetric Ca^2+^ imaging of the caudal half of the brain was performed at 48–56 h.p.f. (referred to as 48 h.p.f.) and 96–104 h.p.f. (referred to as 96 h.p.f.), similar to in ref. ^10^.

#### One-photon spinning disk imaging

Calcium imaging in WHaloCaMP-expressing larvae (Fig. 6a) was performed using a spinning-disk confocal on an upright microscope (Gataca W1). JF_552_ and JF_669_ were imaged with a 561-nm and a 642-nm laser, respectively. The emission filter sets used were EX BP 482/35, BS FT 506, EM BP 536/40 and EX BP 543/22, BS FT 562, EM BP 593/40, respectively. Spinning disk time series were acquired at 5 Hz with an FOV of 330 × 330 μm and using a 40× objective (NA of 1.15).

#### Light sheet for voltage imaging

Voltage measurements were performed using a custom light-sheet imaging system (Fig. 6b). Single-plane illumination was generated by an elongated, low-NA beam focused by an underfilled EC Plan-Neofluar 5×/0.16 Zeiss objective and horizontally scanned using a galvo mirror. The detection path included an XLUMPLFLN 20× objective (Olympus) and a Kinetix camera (Teledyne). Multiple excitation wavelengths were provided by a fiber-coupled laser combiner (L6Cc-CSB-1311-0-488-522-561-638-0-400, Oxxius). Voltron_525_ was excited at 488 nm and detected through a 562/40 filter, while Voltron_585_ was excited with a 561-nm laser and detected using a 593 long-pass filter. All measurements were acquired for 10 s at 500 Hz, over an FOV of approximately 100 × 300 µm, centered on the olfactory epithelium.

#### All-optical experiments using holographic photostimulation

The experiments of Fig. 6g–i were performed using a custom-built microscope in a configuration similar to that used for all-optical recording in ref. ^71^. In brief, the microscope enabled simultaneous 2P scanning imaging (using a Ti:Sapphire infrared laser, Mai Tai, Spectra Physics) and holographic photostimulation (using a fiber amplifier laser at 1030 nm with a 500-kHz repetition rate, Satsuma, Amplitude Systems). Holographic light shaping, based on the use of a spatial light modulator, enabled the reshaping of the 2P stimulation laser to target specific regions of interest (ROIs). In this setup, the system was capable of generating multiple diaphragmed Gaussian spots with an almost top-hat intensity profile, approximately 10 µm in diameter. Combined with temporal focusing^72^, this configuration maintained good axial confinement of the excitation (~15 µm), enabling near single-soma resolution. The setup supported both conventional static illumination and a more efficient, cyclic illumination approach known as FLiT^71^. Based on an initial fluorescence image, early-born and late-born neurons expressing the CoChR opsin were identified. While performing 2P calcium imaging (920 nm, 3 Hz, laser power <10 mW), pulses of holographic illumination (10 pulses, 30 ms, 10 Hz) were delivered selectively to the chosen neurons. Typically, three to seven neurons per FOV were targeted. Photostimulation powers per cell ranged from 5 to 20 mW (power under static illumination).

### Visual stimulation

Zebrafish larvae were paralyzed by 3–5 min incubation in α-bungarotoxin (1 mg ml^−1^) and mounted in a 35-mm Petri dish using 2% (v/v) low-melting-point agarose and maintained at room temperature throughout the recording. Visual stimuli were delivered using a red-emitting projector, shining on a diffusive screen located at a distance of ~3 cm from the larva’s head. The projected pattern covered a 120° horizontal visual field. Slightly different stimulus parameters were used in experiments shown in Fig. 4. For Fig. 4b, the sequence of visual stimuli was (1) bright flash: a single 1-s bright flash stimulus; (2) bright ramp: a gradual increase in brightness over 5 s; (3) gratings: four gratings corresponding to the cardinal directions (0°, 90°, 180° and 270°) with 10°-wide dark stripes moving at 20° s^−1^, where each grating was presented statically in the first set of stimuli, then in motion for 5 s in the second set of stimuli; (4) looming stimuli: a steady looming, a dark circular dot whose dimension expands at a constant rate of 20° s^−1^, followed by an exponential looming, with lateral dimensions doubling every second; and (5) dark ramp: a gradual decrease in brightness over 5 s. For Fig. 4c, the sequence of visual stimuli was (1) dark flash: four 1-s dark flashes with increasing contrast, spaced 20 s apart, followed by a transition to a dark background lasting 20 s; (2) bright flash: four 1-s bright flashes with increasing contrast, spaced 20 s apart, followed by a transition to a bright background; (3) gratings: eight gratings presented in different orientations (0°, 45°, 90°, 135°, 180°, 225°, 270°, 315°; 0° = caudal to rostral and 90° = ventral to dorsal), each with 10°-wide dark stripes moving at 20° s^−1^. Gratings were static for 20 s before moving for 5 s; and (4) looming stimulus (an exponential growth pattern with a radius doubling time of 1 s). Stimuli were programmed and synchronized with calcium imaging recordings, ensuring precise timing and reproducibility.

### Longitudinal functional imaging of locomotor activity in birthdated hindbrain neurons

*Tg(elavl3:H2B-HaloTag)*^*ts25/+*^ and *Tg(elavl3:jGCaMP7s)*^*ts25/+*^ were crossed to obtain *Tg(elavl3:H2B-HaloTag;elavl3:jGCaMP7s)*^*ts25/ts25*^ to perform EMG recording in a paralytic fish. Fish with the paralytic phenotype were dechorionated at 24 h.p.f. and separated for subsequent HaloTag birthdating and functional imaging. The embryos were loaded with JF_585_ at 24 h.p.f. and JF_646_ at 48 h.p.f. using the procedure described for single-pulse birthdating. We used JF_646_ as the second dye because preliminary experiments found it challenging to get enough fluorescence from JF_635_ with our 2P microscope. Volumetric Ca^2+^ imaging of the caudal half of the brain was performed at 48–56 h.p.f. (referred to as 48 h.p.f.) and 96–104 h.p.f. (referred to as 96 h.p.f.) as in ref. ^10^ with a few modifications. Fish were mounted in 1.6% LMP agar dorsal side up, with the tail and right side of the head freed from agar. A concentric platinum iridium stimulation electrode (Microprobes, PI2CEA10-200, MD) was placed on the right side of the head to elicit escape toward the left with a brief electrical stimulus. A glass micropipette was placed on each side of the axial muscles near the anus to record alternating EMG activity during locomotion. The size of the micropipette tip was around 20 µm to minimize the contamination of the EMG signal from the other side, which was noticeable when fish exhibited a strong EMG signal during escape. The signal was amplified (100×) and band-pass filtered (100–1,000 Hz) through an extracellular amplifier (NPI, EXT-02B) and digitized at 6 kHz (National Instruments, PCIe6363). A brief electrical stimulus (0.1 ms in duration, 2–4 V, 1 min interval) was delivered through a glass electrode placed on the side of the head using a stimulus isolator (Digitimer Ltd., DS-2A). The stimulus amplitude was adjusted to evoke strong motor activity consistently before the start of the functional imaging. After the functional imaging, a high-quality structural volume was acquired using a femtosecond laser tuned to 1,120 nm (MKS Instruments, Insight, Deep See) to excite JF_585_ and JF_646_ more optimally alongside GCaMP excited at 940 nm.

### Preparation of CHO cells and patch clamp recordings

CHO cells were acquired from Sigma (Sigma, 85050302) and cultured in T25 flasks (Falcon, 353107) in a medium consisting of DMEM-F12 + Glutamax (Fisher, Gibco, 10565018), supplemented with 10% fetal bovine serum (Fisher, Gibco, 10500064) and 1% penicillin–streptomycin (5,000 U ml^−1^). Cells were passaged every 2–3 days, until P20 to avoid genetic drift. Before each experiment, cells were seeded on coverslips (Fisher, 10252961) in 24-well plates (25,000 cells ml^−1^). After 24 h, cells were transiently transfected with the plasmids pAAV-hSyn-CoChR-HaloTag or pAAV-hSyn-CoChR-GFP. The medium was then replaced after 4 h. Experiments shown in Supplementary Fig. 12 were performed 48–72 h after transfection. A total of 1 h before electrophysiological recordings, CHO cells expressing the CoChR-HaloTag were stained with the fluorescent dye JF_585_ (200 nM). Whole-cell voltage-clamp recordings of opsin-positive cells (GFP-positive or JF_585_-positive) were performed at room temperature (21−23 °C) on the home-made holographic microscope described above. Cells were visualized using infrared differential interference contrast and camera imaging. Cells were continuously perfused with artificial cerebrospinal fluid comprising 125 mM NaCl, 2.5 mM KCl, 1.5 mM CaCl_2_, 1 mM MgCl_2_, 26 mM NaHCO_3_, 0.3 mM ascorbic acid, 25 mM D-glucose and 1.25 mM NaH_2_PO_4_. Continuous aeration of the recording solution with 95% O_2_ and 5% CO_2_, resulted in a final pH of 7.4. Recording pipettes were made from borosilicate glass capillaries using a horizontal puller, resulting in a resistance of 3–5 MΩ when filled with the internal solution, consisting of 135 mM K-gluconate, 4 mM KCl, 4 mM Mg-ATP, 0.3 mM Na_2_-GTP, 10 mM Na_2_-phosphocreatine and 10 mM HEPES (pH 7.35). Recordings were obtained using a Multiclamp 700B amplifier, filtered at 2 kHz and digitized at 10 kHz with a Digidata 1440A interface (Molecular Devices). Data acquisition and analysis were performed using pClamp software (Molecular Devices). Scanning 2P imaging was performed at full field (FOV ~350 µm) with a bidirectional scanning at 3 Hz.

### Imaging on fixed samples from juveniles’ tissues

Larvae or juvenile fish were incubated in JF dyes (one or multiple dyes) at specific time points as indicated in the paper. At specific time points, juvenile fish were prepared for fixation. To do so, they were euthanized using an overdose of 0.2% tricaine methanesulfonate (MS-222, concentration ≥250 mg l^−1^) and ice-cold fish water, and the head was gently cut, fixed in paraformaldehyde 4% in PBS overnight at 4 °C and then washed three times for 10 min in PBS containing 0.1% Triton X-100 (PBST). Subsequently, the brain was carefully dissected under a stereomicroscope (taking care to remove any tissue adhering to the brain) and transferred into a solution of 30% sucrose overnight at 4 °C, for cryoprotection. The next day, they were transferred to plastic molds and embedded in Optimal Cutting Temperature compound (embedding medium for cryotome; Sakura Finetech) after removal of the sucrose. Blocks were then frozen on dry ice and stored at −80 °C. Samples (brains or retinas) were cut with a cryostat at 12–16 µm thickness in the coronal plane. The sections were mounted on Fisherbrand Superfrost Plus slides (Fisher Scientific) and kept at 4 °C before confocal imaging.

### Data analysis

#### Static fluorescent images

For protocols involving incubation with multiple dyes in the same larvae, efforts were made to minimize cross-talk in both excitation and detection of different dyes. Using a confocal microscope, we fine-tuned excitation and detection settings for each dye, achieving negligible cross-talk among the typical JF_525_, JF_552_ and JF_669_ dye combination. The cell count in Supplementary Fig. 1c was performed manually. To maintain consistency, we analyzed the same imaging plane across larvae, using anatomical landmarks as references (one plane per larva, *N* = 14 larvae).

In 2P imaging, the overlap in excitation spectra (with dyes excited at 800–850 nm) and the use of only two PMTs resulted in some fluorescence cross-talk. To address this, the fluorescence intensities of individual dyes were measured in each PMT (with different emission filters) using larvae expressing single dyes. This allowed us to estimate the detection efficiency of each fluorophore and assess potential cross-talk across detection channels. These intensity values were then used as coefficients in a linear wavelength-demixing algorithm, enabling the isolation of the signal contributions and intensities of each individual dye.

#### HaloTag and EdU birthdating analysis

Both HaloTag birthdating and EdU labeling datasets were registered to the ZBB atlas^73^ or the MapZebrain atlas^35^ using the Greedy registration program (https://github.com/pyushkevich). Each channel from an individual 3D volume was converted to an NIfTI file format. Then, the channel corresponding to *Tg(elavl3:H2B-GCaMP6f)* or *Tg(vglut2a:EGFP)* was used as a source to register all the channels to the ZBB atlas using huc-h2b-rfp-ref-01.nii.gz or vglut-ref-01.nii.gz available from Zenodo as a reference volume. The detailed sequence of Greedy commands is shown below (‘Listing 1’). The -sv option was enabled to obtain better behaved deformation fields (https://github.com/pyushkevich).

Listing 1: Greedy registration commands

greedy -d 3 -a -m WNCC 4x4x4 -i {ref_img} {ref_channel_name}.nii \

-o c{ref_channel}_affine.mat -ia-image-centers -n 500x200x0×0

greedy -d 3 -a -m WNCC 4x4x4 -i {ref_img} {ref_channel_name}.nii \

-ia c{ref_channel}_affine.mat -o c{ref_channel}_affine.mat -n 500x200x0×0

greedy -d 3 -rf {ref_img} -rm {ref_channel_name}.nii {ref_channel_name}_affine.nii \

-r c{ref_channel}_affine.mat -float

greedy -d 3 -m WNCC 4x4x4 -i {ref_img} {ref_channel_name}.nii \

-it c{ref_channel}_affine.mat -o c{ref_channel}_warp.nii.gz -sv \

-n 200x200x200x200x0 -s 5.0vox 2.0vox

When the registration failed before warping, we edited the header of the source NIfTI files to bring them close to the reference volume using SPM12 (https://www.fil.ion.ucl.ac.uk/spm/software/spm12/). The registered source file was compared with the reference file. When the boundaries between inside and outside the brain or between the cell body and neuropil area did not match, lower smoothing kernel (-s) parameters were set to increase the flexibility of the warping. When the cell bodies were excessively elongated, higher smoothing kernel parameters were set. The stacks that were not registered properly were excluded from the subsequent analysis.

For the HaloTag birthdating data, each channel file was rescaled such that the median background value was 0 and the 99.99th-percentile value was 1; values exceeding 1 were retained. After this normalization, these channel files were brought to the ZBB atlas space by applying the affine transformation and warping determined by Greedy. Then, the neuronal age group of interest was visualized by subtracting the normalized channel of the preceding dye from the normalized channel of the dye of the interest. The values below zero were set to zero. The population image for each age group was generated by calculating the median of the subtracted images across all individual fish in the group (*N* = 5 or 6 fish per group). In the case of subcellular HaloTag labeling, these processing steps highlight the neuropil area exclusively occupied by the neuronal age group of interest, due to the across-subject variability of individual neurons and the resolution limit of optical microscopy.

For the multipulse HaloTag birthdating, the acquired volumes were fed to the same registration and processing steps used for single-pulse HaloTag birthdating for consistency. A minor modification was made to generate the virtual first dye signal for each target group: the ‘first’ dye signal was defined as the maximum of the normalized channels corresponding to the previous age groups and subtracted from the channel corresponding to the target group.

For the EdU staining data, the channel file corresponding to EdU was normalized as described above and the median of the normalized EdU signal across individual fish was used as the population data (*N* = 5 or 6 per group).

To quantify the area labeled by each age group in the HaloTag dataset, we classified each voxel in the individual volume into the area dominated by the first dye, the area dominated by the second dye and the area not labeled by either of the dyes with the following procedure. First, we applied a median filter (scipy.ndimage.median_filter with the size parameter set to 3) to register and normalized individual volume to make the color ratio metric more robust against the noise. Then, we defined each voxel as a 2D vector represented by the first dye signal and the second dye signal. The length of the vector (*r*) was used to classify voxels between signal (*r* > 0.08) and background (*r* ≤ 0.08) (Supplementary Fig. 5a, dotted red line). Then, the signal voxels were classified between those dominated by the first dye and those dominated by the second dye by applying a GMM (sklearn.mixture.GaussianMixture) to the vector angle (*θ*). Neuronal age group 1 was defined as those dominated by the first dye in treatment group 2 (Fig. 1a) to use the same color ratio-based classification across neuronal age groups. Spurious pixels in the generated binary masks were removed by binary opening (skimage.morphology.binary_opening with a ball with radius set to 2 voxels as a structuring element).

To quantify the area occupied by each age group in the EdU dataset, we used intensity-based voxel classification. To account for the staining variability present in the EdU dataset, we slightly modified the normalization procedure described above and set the value at the top one percentile to 1. Then, we heuristically set the threshold to classify voxel as the signal to 0.6 to exclude the background staining (Supplementary Fig. 5b, see EdU). The same median filter and binary opening procedures used in the HaloTag dataset were used for the EdU dataset.

To determine the percentage area occupied by each age group, we generated two binary masks: the one that captures the central nervous system including the areas occupied by progenitors and neuropil (Supplementary Fig. 5b, ‘brain mask’) and the other that captures the area occupied by neuronal nuclei (Supplementary Fig. 5b, ‘neuron mask’). The neuron mask was created to exclude the difference coming from the EdU labeling of the progenitors. The brain mask was generated from the ‘huc-brain’ stack for the ZBB atlas by excluding the peripheral nervous system (olfactory epithelium and posterior lateral line ganglion) to remove the area not included in the EdU samples. For the MapZebrain atlas, we generated the brain mask using the V2 regions (https://api.mapzebrain.org/media/downloads/Regions/mapZebrain__regions__v2.0.1.zip) by excluding the areas outside the central nervous system. For both atlases, the neuron mask was generated from the registered stacks for treatment group 5 in the HaloTag dataset that label all neurons in the 5-d.p.f. zebrafish. We categorized each voxel with its vector length (*r*) larger than 0.02 to be the area occupied by neuronal nuclei in individual volumes. Then, we generated the population mask by taking the median of the binary masks from individual volumes.

To statistically examine if birthdating methods and the targeted neuronal age groups explained the variance in the proportion of the volume labeled in each age group, we used a generalized linear model with a logit link function for binomial distribution using the stats package in R as follows:

$$\begin{array}{l}{\rm{Presence}}\;{\rm{of}}\;{\rm{labelled}}\;{\rm{voxel}}\sim{\rm{birthdating}}\_{\rm{method}}\times{\rm{target}}\_{\rm{group}}\\ {\scriptstyle(\sim\;\equiv\,\textquoteleft {\rm{is}}\;{\rm{ modeled}}\;{\rm{as}}\;{\rm{a}}\;{\rm{function}}\;{\rm{of}}\textquoteright)}\end{array}$$

Then, we assessed the main effects of birthdating methods and target group by comparing the above model with reduced models with ANOVA. We also performed the pairwise comparison of birthdating methods within the same target group with Tukey post hoc test using the emmeans package (https://cran.r-project.org/web/packages/emmeans/index.html). Cross-correlaftion analyses were performed on the median volume generated from each target group to evaluate the similarity across the birthdating methods and the overlap between target age groups within each birthdating method.

#### Calcium imaging analysis during visual stimulation

Calcium transients from GCaMP fluorescence signals of Fig. 4 were analyzed either by integrating over macro-regions or by extracting signals from individual cell bodies. (1) Macro-regions were defined as areas containing mainly either early-born or late-born neurons, based on the global distribution of the two dyes, JF_552_ and JF_669_. Specifically, the edges of the contralateral side of the OT (opposite to the visually stimulated eye) were delineated using the JF_669_ staining (second dye, incubated at 5 d.p.f.). In this region, both dye images were smoothed with a Gaussian filter (*σ* = 8 µm) and, within each half of the rostral and caudal portions of the OT, late-born domains were identified as areas lacking JF_552_ signal (defined as pixels with intensities below the 20th–30th percentile of the JF_552_ distribution), which corresponded to the most peripheral tectal zones. Equally sized regions containing both dye signals were then defined within the central tectal area and considered to be populated by early-born neurons (born before the first dye incubation).

GCaMP transients within these macro-regions were integrated and converted to ∆*F*/*F*_0_ signals, where *F*_0_ was defined as the 20th quantile of the full intensity distribution. (2) For single-cell analysis, GCaMP fluorescence was extracted from individual neurons of the contralateral OT identified using CellPose (cyto2 model applied to JF dyes fluorescence) with manual refinement of ROIs in ImageJ. After background subtraction and motion correction, GCaMP transients were converted to ∆*F*/*F*_0_ signals, with *F*_0_ calculated as the time-averaged intensity of each trace. For each neuron, the unmixed ratio of the two incubated dyes was also computed from the same ROI. Peaks of ∆*F*/*F*_0_ signals were defined as the maximum values within a 3-s time window starting at the onset of each visual stimulus.

To compare early- and late-born neuron activity without dividing the dataset into arbitrary clusters, we computed the correlation between the Δ*F*/*F* signal and the dual-dye fluorescence ratio for all neurons using SciPy’s spearmanr function. The resulting Spearman coefficients, which reflect the strength of the correlation between the two variables, were plotted as a function of time. The threshold for significance was determined using Student’s *t*-distribution. More explicitly, the resulting Spearman coefficient plots (Fig. 4d,g and Supplementary Figs. 16 and 17) show the correlation between the Δ*F*/*F* values and the dye ratio of each neuron at each time point (1 = perfectly correlated, 0 = not correlated, −1 = negatively correlated). Time points with significant correlations are shown in red (the gray line indicates the threshold for significance). Note that under the null hypothesis, one still expects 5% of time points to reach significance (±1%, binomial confidence interval, *α* = 0.05). In the main Fig. 4, for instance, 42% of time points are significant, indicating correlations between activity and dye fluorescence. These significant time points are not distributed uniformly; rather, the correlation is stronger—and therefore more frequently significant—during visual stimulation, suggesting that early-born neurons are more likely to be activated by the stimuli than late-born neurons.

Separation of the neuropil interneurons into the two subpopulations, SINs and DINs (Supplementary Fig. 10), was based on their anatomical location. For each cell soma, we extracted its distance from the skin (perpendicular distance from the line connecting the intersection of the skin with the periventricular zone), and we performed a dual-Gaussian fit of this distance distribution. A distant value corresponding to approximately 3*σ* (~15 µm) of the first Gaussian fit (imposed to be centered at 0) was set as the threshold to define SINs (distance <3*σ*), while the remaining, more distal neurons (distance >3*σ*) were defined as DINs.

For the direction selectivity (DSI) analysis, only neurons that responded to at least one of the gratings were selected. The response amplitudes were defined as the maximum ∆*F*/*F* over the ten frames following the stimulus onset. Responsive neurons were defined when response amplitudes were larger than four times the s.d. (evaluated in the 20 frames before stimulus onset). For each of the eight moving-bar stimuli, a vector was defined by its angle corresponding to the bar direction and its response amplitude. The DSI was calculated as the magnitude of the vectorial sum of these eight vectors, normalized by the sum of their individual magnitudes (Extended Data Fig. 6d). We separated neurons into two equal-sized groups (according to the dye ratio) and plotted the distribution of preferred directions (Extended Data Fig. 6e).

#### Spontaneous calcium imaging analysis

WHaloCaMP fluorescence transients from spinning-disk recordings were extracted by manually defining ROIs over individual cell bodies, thereby identifying each tectal neuron (>600) present in the recorded plane. After motion correction and registration of the two color images, fluorescence values of each individual neuron were combined to compute their dual-dye intensity ratio (JF_552_/(JF_552_ + JF_669_)). This ratio was used to cluster neurons into early-born and late-born populations (using *k*-means clustering, *k* = *2* clusters; Supplementary Fig. 11b). After ROI-based background subtraction, raw fluorescence time traces were extracted for each neuron by spatially integrating signals over the corresponding ROI. Following detrending, Δ*F*/*F* was computed using a baseline defined as the tenth percentile of each trace, with baseline values verified by manual inspection. Before quantifying activity, traces were denoised using second-order autoregressive deconvolution implemented through the OASIS package^74^, which provided more meaningful denoising than smoothing or low-pass filtering approaches. Activity quantification was then performed on the denoised traces using automatic peak detection (minimum peak prominence >2 × signal-to-noise ratio (SNR); minimum peak distance: 25 frames). Neurons were initially classified as active if they exhibited at least one Δ*F*/*F* event exceeding 3%. Automatic classifications were subsequently refined through manual verification using a custom MATLAB GUI that enabled rapid browsing of individual traces and user-guided adjustment of detected peaks and activity status. The resulting metrics, mean peak frequency and mean peak amplitude (averaged across all events of each cell), are reported for active cells (Fig. 6c) and were used to compare spontaneous activity between early-born and late-born neuronal populations. Importantly, previous characterization^45^ demonstrated similar sensitivity of WHaloCaMP transients when using either JF_552_ or JF_669_ dyes, ensuring that observed differences in fluorescence reflect variations in neuronal activity between the two populations. In addition, the high selectivity in the excitation and detection of each dye minimizes cross-talk, avoiding contamination of the functional signal due to dual-color labeling.

#### Spontaneous voltage imaging analysis

Voltron2-JF_552_ and Voltron2-JF_585_ fluorescence transients obtained from light-sheet imaging were recorded from labeled neurons in the zebrafish olfactory epithelium. The sparse labeling in the tissue enabled manual identification of neurons predominantly expressing either JF_552_ or JF_585_, corresponding to early-born and late-born cellular populations, respectively. Fluorescence traces were extracted by integrating signal intensities over ROIs placed on individual cell bodies, followed by background subtraction and detrending. Δ*F*/*F* was then computed for each neuron. Cells exhibiting narrow (<3 ms) Δ*F*/*F* transients above the noise level, corresponding to putative spiking activity, were classified as active. Spiking frequency was quantified by manually counting spikes in these active neurons.

#### Analysis of longitudinal functional imaging of the hindbrain

##### Image processing

The GCaMP channel of the functional imaging data was used as a source to determine the mismatch between the odd and even scan lines and the lateral shift of the sample in each plane by phase cross-correlation (the phase_cross_correlation function from scikit-image library). Then, these corrections were applied to all the channels, and a high SNR volume was generated for each channel by averaging across all the time points. Bleed-through of the GCaMP signal to the JF_585_ and JF_646_ channels was estimated on the basis of the voxels in the top 10% of GCaMP signals with a linear regression. The JF_585_ and JF_646_ channels were subtracted with the bleed-through estimated voxel by voxel, and then the subtracted volume was normalized between the median and 99.99-percentile voxel values as described above. The summed image of the two JF dye signals was then upsampled at two times higher resolution and fed to the nuclei detection algorithm on the basis of local contrast^75^. Then, the JF_585_ and JF_646_ signals from each of the detected nuclei were extracted to define the two neuronal age groups on the basis of the vector angle defined by the normalized JF_585_ and JF_646_ signals, $\arctan (\frac{{\left\langle {S}_{\mathrm{JF}646}\right\rangle }_{\mathrm{norm}}}{{\left\langle {S}_{\mathrm{JF}585}\right\rangle }_{\mathrm{norm}}})$. As a first step, the local peaks detected in the skin with high autofluorescence in the JF585 channel were removed by applying GMM on the vector angle using GaussianMixture from scikit-learn. This group was operationally defined as one of the three components detected by the GMM with the lowest vector angle because the skin autofluorescence was mostly absent in the JF_646_ channel. Subsequently, remaining local peaks from neuronal nuclei were divided into two neuronal age groups using sigma clipping. This procedure is motivated by our observation that the second age group corresponds to the long tail on the higher angle. Thus, the second age group was defined as the group with the angle higher than three s.d. away from the Gaussian distribution detected by sigma clipping. The ∆*F*/*F*_0_ of GCaMP signal from individual neurons was calculated by dividing the raw fluorescence signal from each identified nucleus with the baseline estimated as a 20-percentile value within a 60-s rolling window and used in the following regression and clustering analyses.

#### Analysis of locomotor activity

EMG traces were processed to detect stimulus-induced escapes and spontaneous swims as follows. First, large transient deflections caused by the electrical stimuli were identified by the find_peaks function from scipy library and the time windows dominated by the artifacts were blanked (typically −1.5 to +5 ms around the peak of the artifact). A slow-decay component of the artifact after large transients was modeled by the interp1d function from SciPy and subtracted from the trace. Then, the EMG traces from the left and the right sides of the axial muscles were processed as follows to generate a smoothed EMG amplitude estimate robust against noise. They were first *z*-score normalized, then their power was calculated in a Gaussian-kernel window (8 ms at full-width at half maximum), and its square root product was used as a robust EMG amplitude estimate. Then, the peaks corresponding to each burst of muscle activity were detected by the find_peaks function for the left and right EMG traces separately. To reject the peaks corresponding to the strong EMG bursts from the other side, the following procedure was taken. After merging the peaks from both sides, each pair of neighboring bursts was compared, and if the interpeak interval was shorter than 6 ms, the smaller peak was excluded from further analysis. Then, swim episodes were defined as a series of bursts with inter-burst intervals below 70 ms. Instances of burst groupings with fewer than four bursts (corresponding to two left–right alternations) were not classified as swim episodes.

#### Regression analysis of locomotor activity

To map the neurons recruited during distinct types of locomotor activity, regression analysis was performed on ∆*F*/*F*_0_ of the GCaMP signal extracted from individual nuclei using regressors representing distinct swim events. In both developmental stages, escape events were defined on the basis of the electrical shock that reliably induced escape behavior. In 48-h.p.f. embryos, another regressor was constructed to capture the variability in the length of shock-induced swim episodes by treating each detected burst from EMG traces from both sides. In 96-h.p.f. embryos, we constructed three regressors besides the escape regressor based on the expanded range of EMG burst amplitudes (Fig. 5c), similarly to ref. ^10^. Bursts from each EMG trace were classified as ‘weak’ or ‘strong’ using a GMM with two components. Then, the ‘strong’ bursts from left and right EMG traces were treated as separate events on the basis of our previous observation that distinct sets of neurons are recruited on the basis of the side of strong locomotor activity. The ‘weak’ bursts from left and right EMG traces were combined together as in ref. ^10^ given the slow kinetics of jGCaMP7s relative to left–right alternating bursting activity. Regressors were constructed by convolving each of these locomotor event types with the GCaMP6s impulse response function given its similar kinetics to jGCaMP7s^76^ and then *z*-score standardized for each session. Neuronal ∆*F*/*F*_0_ response matrix (*Y*) was fitted with these standardized regressors (*X*) using the following linear model, *Y* = *Xβ* + *ϵ*. The standardized coefficient (*β*) was estimated by ordinary least squares, and then the *T* value for each neuron was calculated on the basis of the standardized coefficient (*β*) and residual (*ϵ*). Correction for multiple comparisons was performed with the false discovery rate (FDR). The threshold for *T* maps was set at *P*_FDR_ < 0.05. The neurons that met these criteria for a given locomotor event were classified as active neurons. To statistically examine if neuronal age and subject age explained the variance in the proportion of active neurons in each group, we used a generalized linear mixed model with a logit link function for binomial distribution using the lme4 package in R (https://cran.r-project.org/web/packages/lme4/index.html) as follows:

$$\begin{array}{c}{\rm{P}}{\rm{r}}{\rm{e}}{\rm{s}}{\rm{e}}{\rm{n}}{\rm{c}}{\rm{e}}\,{\rm{o}}{\rm{f}}\,{\rm{a}}{\rm{c}}{\rm{t}}{\rm{i}}{\rm{v}}{\rm{e}}\,\mathrm{neuron}\,\sim\mathrm{neuron\_}{\rm{a}}{\rm{g}}{\rm{e}}\times {\rm{s}}{\rm{u}}{\rm{b}}{\rm{j}}{\rm{e}}{\rm{c}}{\rm{t}}{\rm{\_}}{\rm{a}}{\rm{g}}{\rm{e}}\\ +(1|{\rm{s}}{\rm{u}}{\rm{b}}{\rm{j}}{\rm{e}}{\rm{c}}{\rm{t}}{\rm{\_}}{\rm{i}}{\rm{d}})+(1|{\rm{l}}{\rm{o}}{\rm{c}}{\rm{o}}{\rm{m}}{\rm{o}}{\rm{t}}{\rm{o}}{\rm{r}}{\rm{\_}}{\rm{t}}{\rm{y}}{\rm{p}}{\rm{e}}).\end{array}$$

Besides subject identity, locomotor type was also treated as a random effect because 2-d.p.f. and 4-d.p.f. fish showed distinct locomotor behavior. After observing statistically significant effects of subject age and neuronal age and their interaction, post hoc Tukey tests were carried out using the emmeans package in R to correct for multiple comparisons.

#### Functional clustering of the hindbrain activity

To quantify the proportion of active neurons and number of functional clusters, the computed GCaMP ∆*F*/*F*_0_ from all the identified neurons were first *z*-score normalized over time and then subjected to the Rastermap algorithm^48^ to sort them on the basis of the similarity of their activity within each neuronal age group. Parameters were set as follows: n_PCs = 50, n_clusters = 100, locality = 0.1, time_lag_window = 0. Neuronal activity was averaged in bins of 50 neurons; the averages of these neurons are called ‘superneurons’. Those that showed the 99th-percentile *z*-score across the duration of the experiment above 0.4 were considered active populations. The threshold was chosen to capture active neurons without including those with no clear activity pattern in the 2-d.p.f. zebrafish data (Fig. 5f, 2 d.p.f.). Then, the proportion of active population within each age group was compared using a generalized linear mixed model with a logit link function for binomial distribution using the lme4 package as follows:

$${{\rm{Presence}}\;{\rm{of}}\;{\rm{active}}\;{\rm{neuron}}}\sim{{\rm{neuron}}\_{\rm{age}}\times{\rm{subject}}\_{\rm{age}}+(1|{\rm{subject}}\_{\rm{id}}}).$$

Then, within-factor comparisons were performed using the emmeans package in R with the multiple comparison correction with the Holm method.

To assess the diversity of neuronal activity within each neuronal age group, the active populations were clustered by applying hierarchical clustering to their activity. We used the Ward variance minimization algorithm (scipy.cluster.hierarchy.linkage) and set the distance threshold to 30, which reliably captured two distinct activity patterns present in the 2-d.p.f. data that show escape-related activity and long-swim-related activity. Then, the proportion of active population and the number of distinct functional clusters within each age group were compared using linear mixed model as follows:

$${{\rm{Number}}\;{\rm{of}}\;{\rm{clusters}}\;}\sim{{\rm{neuron}}\_{\rm{age}}\times{\rm{subject}}\_{\rm{age}}+(1|{\rm{subject}}\_{\rm{id}}})$$

Within-factor comparisons were carried out in the same way the proportion of active neurons was assessed.

For visualization of the sorted activity irrespective of neuronal age groups (Extended Data Figs. 7 and 8), the Rastermap algorithm was applied to the entire neuronal population using the same set of parameters. The average age of a ‘superneuron’ (50 neurons) was computed by averaging the age of the individual neurons in each superneuron. The sorted neurons were subdivided into 18 functional groups on the basis of their position in the Rastermap sorting. Neurons in each functional group were divided into two neuronal age groups and visualized in a top-down view.

#### Analysis of all-optical experiment

For the all-optical experiments in Fig. 6g–i, fluorescence signals were integrated over 8-µm circular ROIs centered on individual somata selected for holographic stimulation. Photostimulation artifacts were semi-automatically detected and removed using a GUI-based MATLAB script. The percentage change in fluorescence was calculated as ∆*F*/*F* = (*F* − *F*_0_)/*F*_0_, where *F*_0_ is the baseline fluorescence level. *F*_0_ was defined within the MATLAB GUI as the average fluorescence over a ~3-s time window preceding holographic stimulation and manually adjusted when necessary, to exclude distortions from spontaneous activity. Photoinduced ∆*F*/*F* peaks were automatically identified, manually verified and averaged across stimulation events.

### Statistics and reproducibility

No statistical method was used to predetermine sample sizes, but our sample sizes are similar to those generally used in the field. For each experiment, fish were chosen at random from batches of healthy siblings that had developed normally, and, unless otherwise stated, all randomly sampled fish were included in the analyses; any exclusion criteria are specified in the corresponding sections (for example, image volumes that could not be properly registered to the reference atlas were excluded). Data collection and analysis were not performed blind to the conditions of the experiments. For readouts that were not normally distributed (for example, the proportion of labeled volume or of active neurons), we did not rely on statistical tests that assume normality and equal variance; instead, we used generalized linear and generalized linear mixed models with link functions appropriate to the data distribution (for example, a logit link for binomially distributed proportions), as well as Kruskal–Wallis ANOVA, Kolmogorov–Smirnov and Spearman tests. The assumptions of these models—approximate normality and equal variance of the residuals on the link-function scale—were examined rather than formally tested, and the data met these assumptions. Student’s *t*-tests and parametric ANOVAs were performed after normality, and homogeneity of variance were formally tested using Shapiro–Wilk and Levene tests, respectively. The Welch’s *t*-test was used for normally distributed data with unequal variances.

### Reporting summary

Further information on research design is available in the Nature Portfolio Reporting Summary linked to this article.

## Online content

Any methods, additional references, Nature Portfolio reporting summaries, source data, extended data, supplementary information, acknowledgements, peer review information; details of author contributions and competing interests; and statements of data and code availability are available at https://doi.org/10.1038/s41593-026-02423-9.

## Supplementary information

Supplementary Information Supplementary Figs. 1–14 and Table 1.

Reporting Summary

## Source data

Source Data Fig. 1 Statistical source data for Fig.1c,d.

Source Data Fig. 4 Statistical source data for Fig. 4b–g.

Source Data Fig. 5 Statistical source data for Fig. 5e,g,h.

Source Data Fig. 6 Statistical source data for Fig. 6b,c,e,f,h,i.

Source Data Extended Data Fig. 6 Statistical source data for Extended Data Fig.6b–e.

## Data Availability

Source data are provided with this paper. Additional data are available on request.

## Code availability

Analysis codes and exemplary accompanying data are available via GitHub at https://github.com/koyama-lab/faini-et-al.
