# Supplementary material for: CLOK: a chemigenetic multicolor labeling system to visualize neuronal birthdate and circuit integration
Source: Nat Neurosci. 2026 Sep 30;29(11):2861–73. doi: 10.1038/s41593-026-02423-9 (PMC13724276; doi:10.1038/s41593-026-02423-9)
Supplement: Supplementary file 1 [file 41593_2026_2423_MOESM1_ESM.pdf]

nature neuroscience

Supplementary information

https://doi.org/10.1038/s41593-026-02423-9

CLOK: a chemigenetic multicolor labeling system to visualize neuronal birthdate and circuit integration

In the format provided by the authors and unedited

Supplementary Figures:

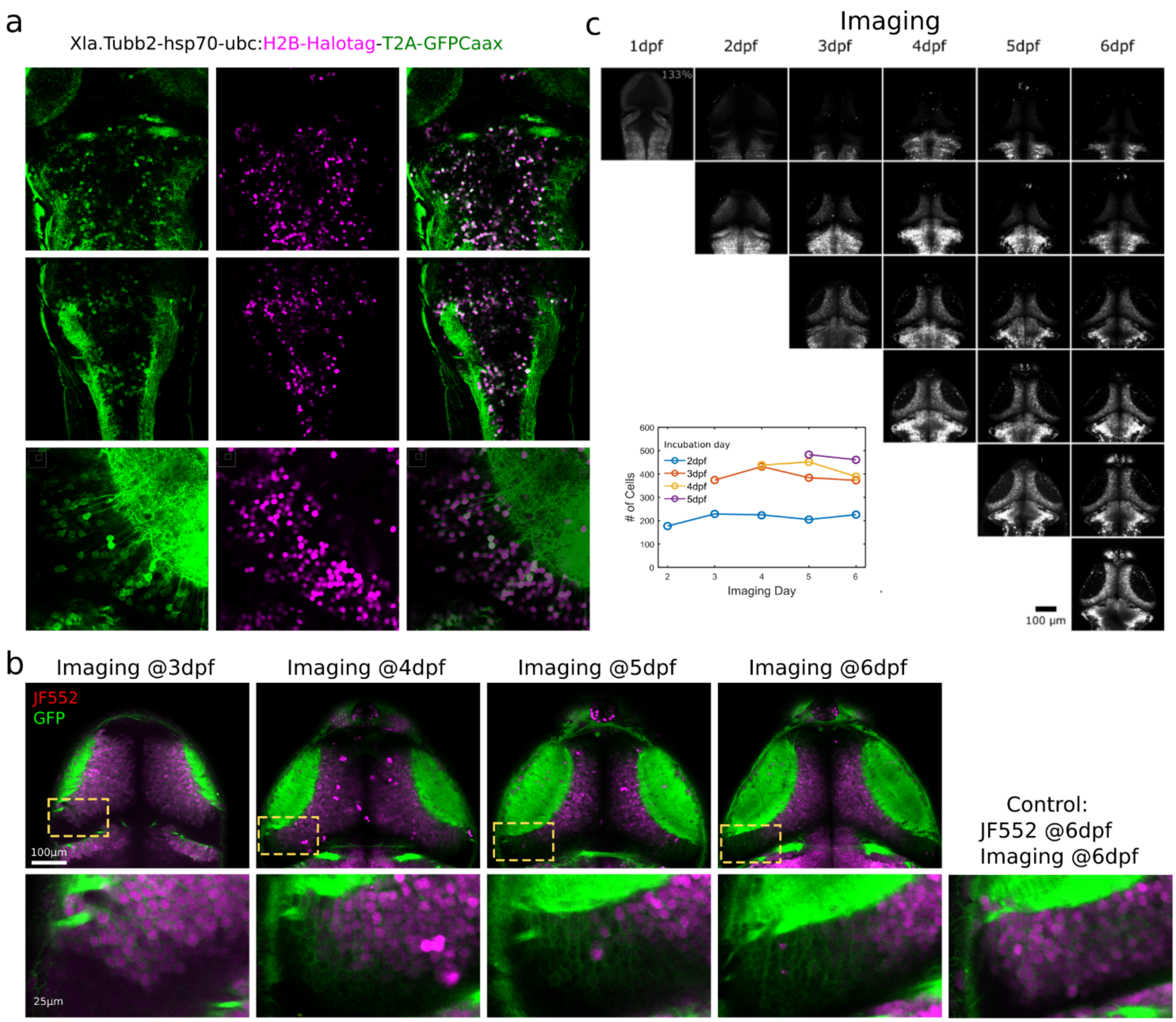

Supplementary Figure 1. Efficiency, temporal specificity and developmental stability of JF dye loading. a, Images from larvae co-expressing GFP (in the membrane) and Halotag (in the nucleus) - Transient expression of *Xla.Tubb2-hsp70-ubc:H2B-HaloTag-T2A-GFPcaax*. After incubation with JF_{552}, colocalization of the dye (red) and GFP (green) was evaluated to estimate the efficiency of the Halotag labelling (N = 3 larvae; 97.43 ± 1.8 %). **b**, Images from the Tg line (*Xla.Tubb2-hsp70-ubc:H2B-HaloTag-T2A-GFPcaax*). Larvae were incubated at 3dpf (with JF_{552}) and imaged at different time points ranging from 3 to 6 dpf. The longer the delay between incubation and imaging, the more JF_{552}-negative, GFP-positive cells were observed. This reflects neurons having started to express the transgenic reporters after the dye incubation and thus expressing Halotag that had not been labeled by the dye (N = 3 larvae per condition). **c,** Set of confocal images from larvae (*Tg(Xla.Tubb2-hsp70-ubc:h2b-Halotag*)) labeled with a single dye at specific time points and imaged at different dpf. In the inset, we report the number of labeled cells in the optic tectum for the same labeling timepoints across days (N = 14 larvae).

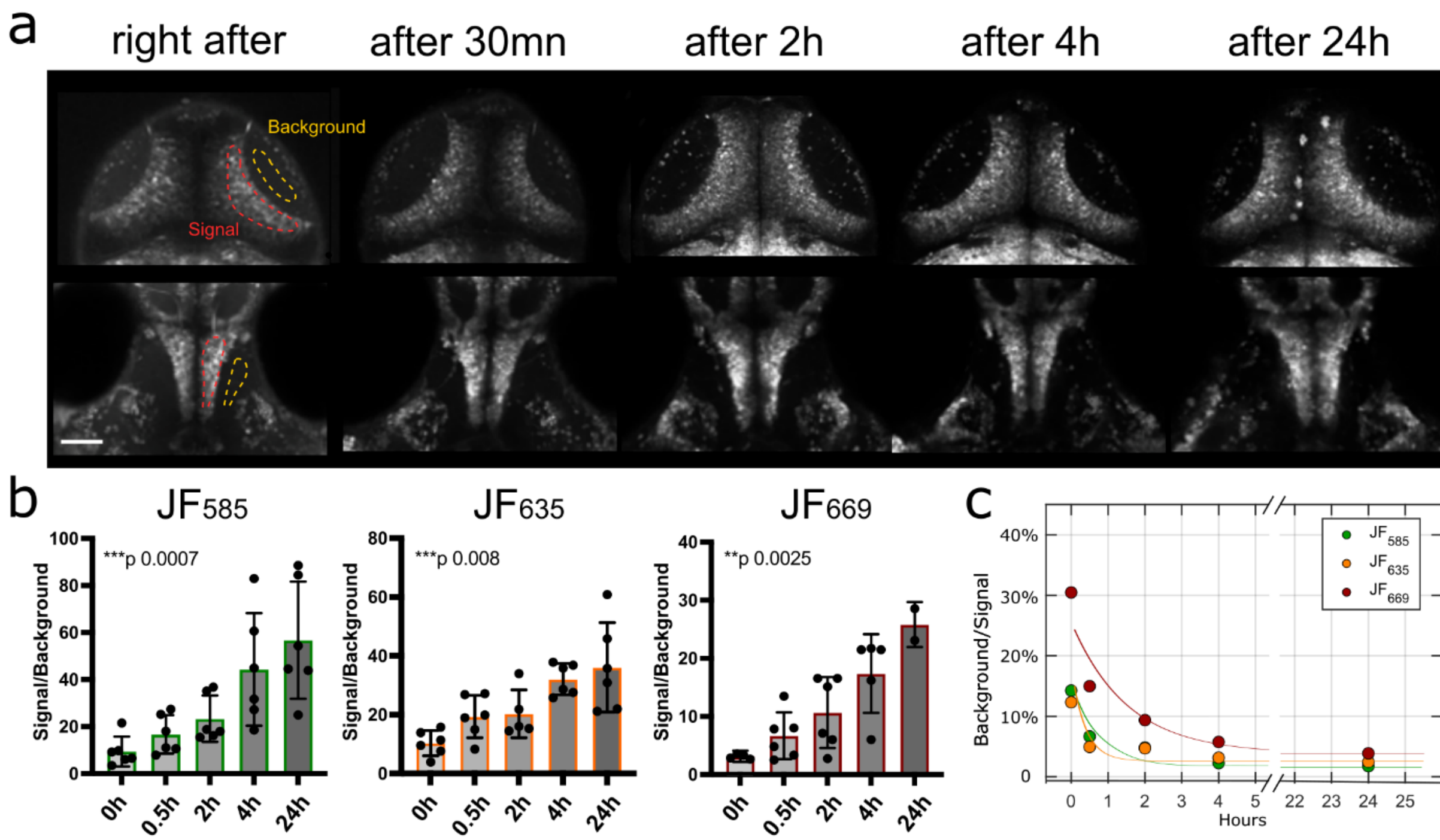

Supplementary Figure 2: Dye washout and image contrast over time. a, Representative images of larval brains incubated with JF_{699} at 4dpf and imaged at different time points after incubation, ranging from 0 to 24h. Dotted closed paths indicate ROIs used to extract fluorescence from labelled cells (signal, red) or nonspecific labelling (background, orange). N = 3 larvae**. b,** Histograms of contrast values (signal/background) at different time points after incubation (JF_{585}, JF_{635} and JF_{669} at 4dpf). N = 3 larvae; 2 ROIs at different depths per larva. 1-Way ANOVA. **c,** Normalized background intensity over time after incubation for three different JF dyes. The lines show exponential fits of the data points of each dye. Scale bars: 100µm. Tg line used: *Xla.Tubb2-hsp70-ubc:h2b-Halotag*. N = 3 larvae.

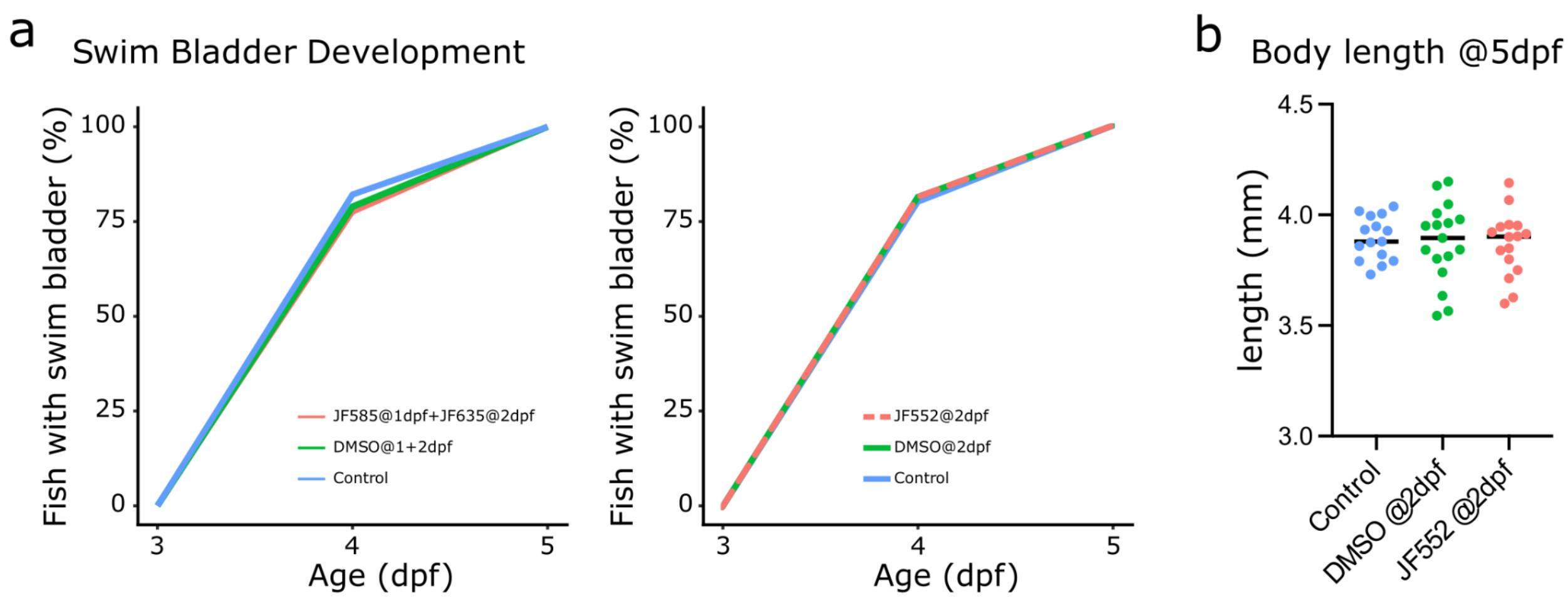

Supplementary Figure 3. Effect of dye loading on larval development. a, Swim bladder development of larvae incubated with two consecutive (left panel) or one single (right panel) dye, compared to DMSO-treated and control siblings. Left: N = 30 fish per group; Right: N = 15 fish (Control), N = 17 fish (DMSO), 16 fish (JF_{552}). **b,** Body length of JF_{552}-treated larvae (at 2dpf) and measured at 5dpf, compared to DMSO-treated and control siblings. $p = 0.97$, One-way ANOVA. N = 15 fish (Control), N = 17 fish (DMSO), 16 fish (JF_{552}).

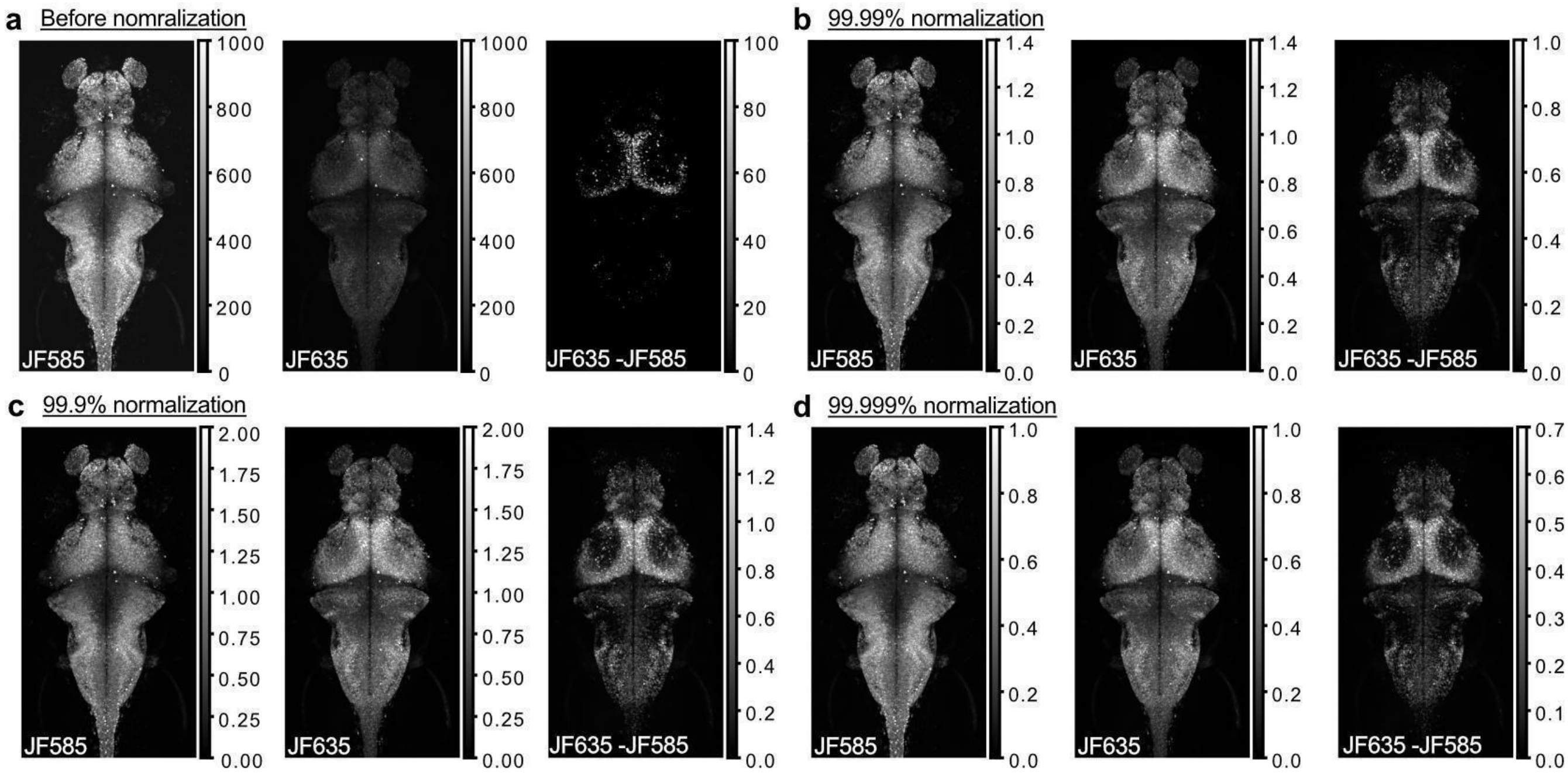

Supplementary Figure 4. Effects of normalization to the subtraction-based visualization. a, Z-projected volumes of the first and the second dye signals and the subtracted signals without normalization. **b,** Z-projected volumes after normalization using 99.99 percentile value. Each panel is organized as in a. **c,** Z-projected volumes after normalisation using 99.9 percentile value. **d,** Z-projected volumes after normalisation using 99.999 percentile value. Despite the difference in the range of voxel values, the subtracted volumes look similar across the normalization parameters except the one without normalization (N = 5 or 6 fish per age group).

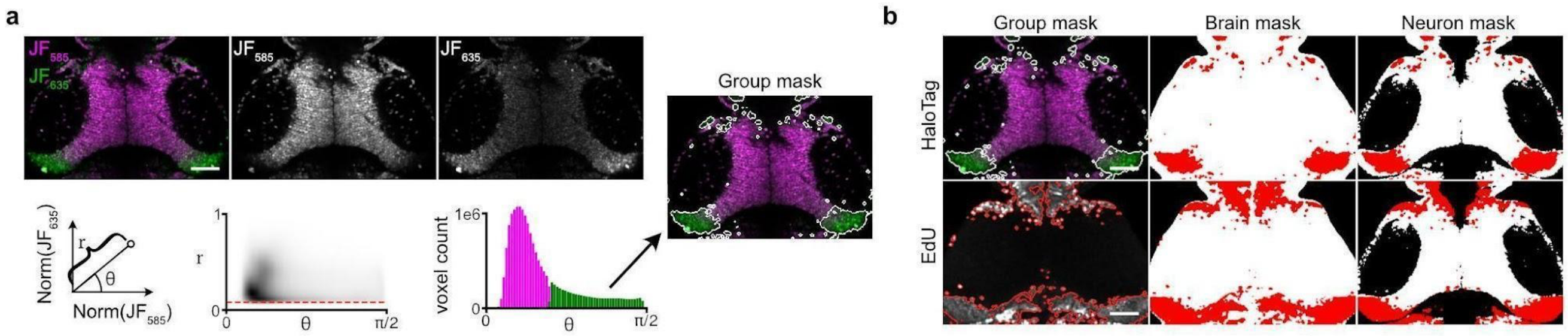

Supplementary Figure 5. Quantification of the area occupied by a neuronal age group. a, Image processing steps to determine the area labeled by a single-pulse CLOK labeling. Normalized dye signals are shown in a single slice in the tectum of a single subject from neuronal age group 4. The ratio of two dyes is represented as the angle of a 2d vector composed of normalized dye signals and classified into two clusters using Gaussian Mixture Model (see Methods). The voxels classified as a part of the age group are demarked with white closed paths. **b,** Masks representing CLOK labeling and EdU labeling and their spatial position relative to the brain and the area occupied by neuronal nuclei. These masks are shown in a single slice in the tectum of a single subject in neuronal age group 4. The area labeled by single-pulse CLOK is indicated as in (a). The area labeled by EdU is indicated with red closed paths on top of the normalized EdU signal. Then, the percentage volume of each mask is calculated within the entire brain volume (Brain mask) and in the volume that excludes the ventricular and neuropil zone (Neuron mask). The labeled area within each mask is shown in red. Scale bars: 50μm.

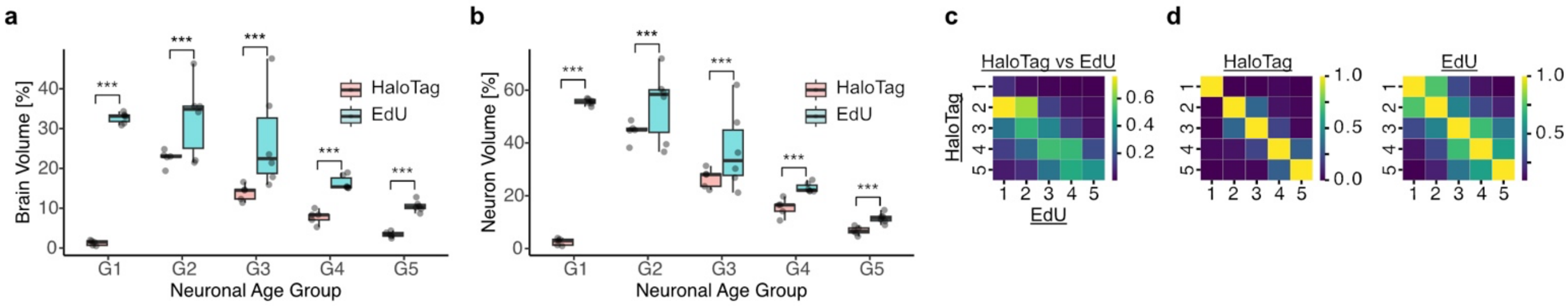

Supplementary Figure 6. Quantification of the area occupied by a neuronal age group in the MapZebrain atlas. a, Percentage brain volume occupied by each age group (5 or 6 fish per target age group). Boxplots show the median (center line), the 25th and 75th percentiles (lower and upper hinges of the box), and whiskers extending to the most extreme data point within 1.5 × the interquartile range of the hinge. *** $p < 0.001$, Tukey post hoc test after per-voxel binomial regression (G1, $z = 5502.7$; G2, $z = 2227.5$; G3, 3224.6; G4, $z = 2586.6$; G5, $z = 2656.0$). **b,** Percentage neuron volume occupied by each age group (5 or 6 fish per target age group). Ventricular and neuropil zones are excluded from quantification. Boxplots show the median (center line), the 25th and 75th percentiles (lower and upper hinges of the box), and whiskers extending to the most extreme data point within 1.5 × the interquartile range of the hinge. *** $p < 0.001$, Tukey post hoc test after per-voxel binomial regression (G1, $z = 5650.9$; G2, $z = 1401.0$; G3, $z = 1629.5$; G4, $z = 1344.4$; G5, $z = 1162.0$). **c,** Cross correlation of the neuron volume occupied by each age group across CLOK and EdU data**. d,** Cross correlation of the neuron volume occupied by each age group within each dataset.

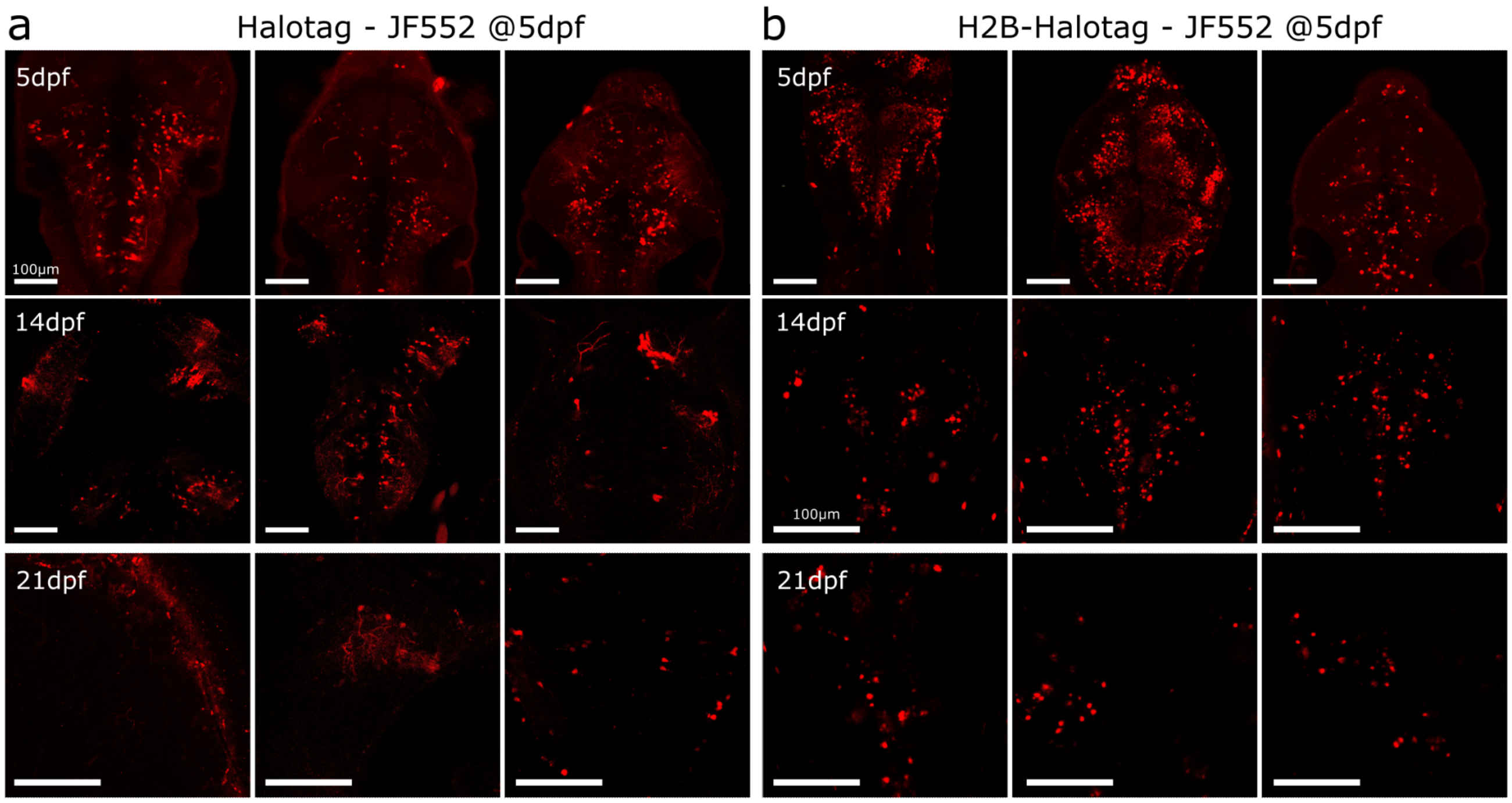

Supplementary Figure 7. Comparison of JF signal in larvae transiently expressing Halotag protein alone and Halotag protein fused with H2B tag. a, Confocal images of larvae transiently expressing the plasmid Xla.Tubb2-hsp70-ubc:Halotag and imaged at 5, 14 and 21 dpf. Larvae were incubated with JF_{552} at 5dpf. **b,** Confocal images of larvae transiently expressing the plasmid Xla.Tubb2-hsp70-ubc:H2B-Halotag and imaged at 5, 14 and 21 dpf. Scale bars: 100µm. In both cases, clear JF_{552} signal is detectable up to 21 days after the labelling. N = 3 larvae per experiment.

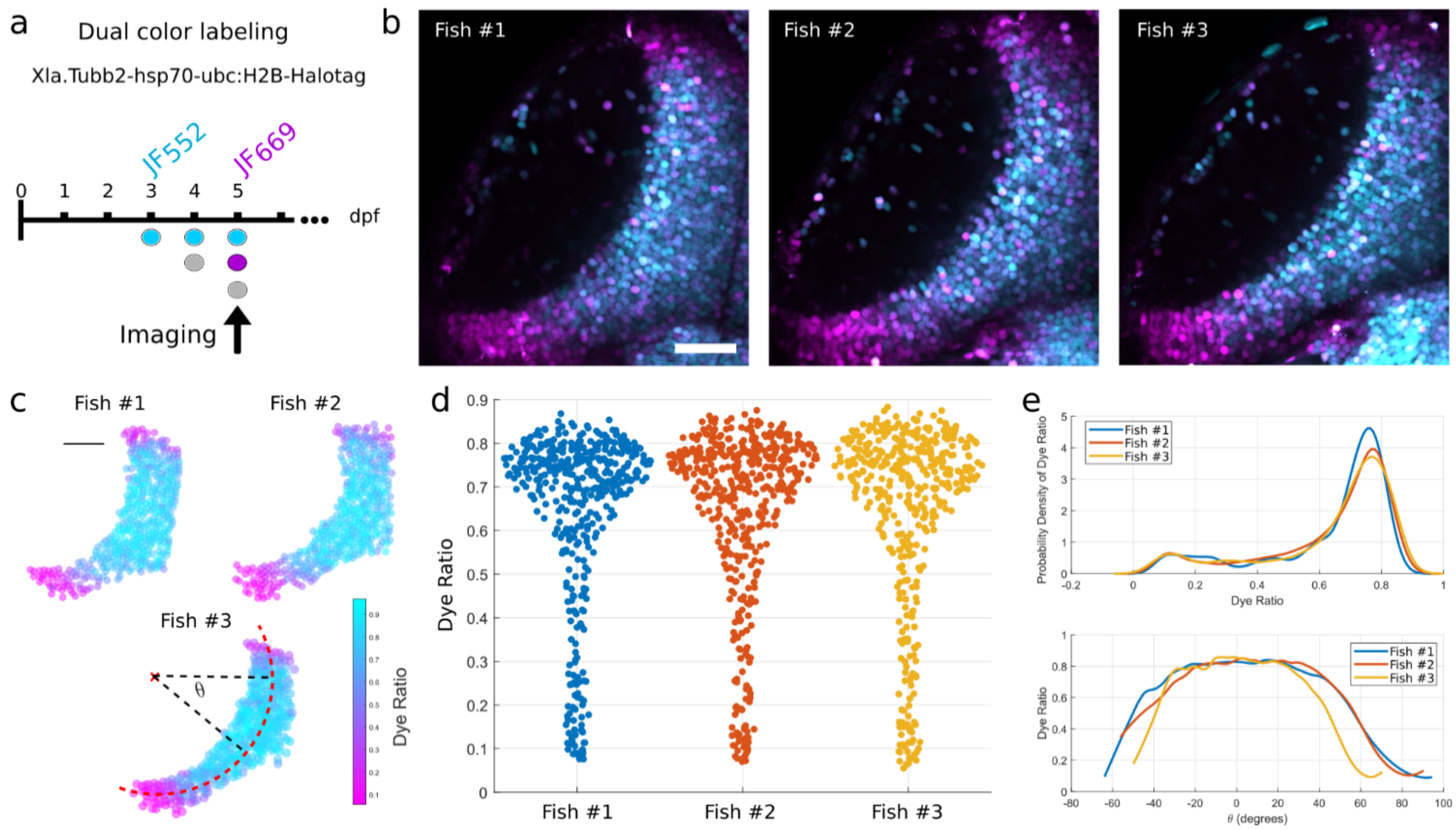

Supplementary Figure 8. Replicability and dye intensities distribution in dual color CLOK. a, Dual-color labeling in *Tg(Xla.Tubb2-hsp70-ubc:H2B-Halotag)* at 2 different time points during development (JF_{552} at 3dpf, JF_{669} at 5dpf, confocal imaging at 5dpf). **b,** Fluorescence dual color images of the OT (N = 3 larvae). Cyan and magenta correspond to signals from JF_{552} and JF_{669}, respectively. Scale bar: 50µm. **c,** Scatter dots representing the position of neurons in the zebrafish optic tectum extracted from larvae in panel (b), color-coded according to their individual dye intensity ratio $JF_{552}/(JF_{552}+JF_{669})$. Scale bar: 50µm. In the bottom panel, an example of an arc of circumference fit of the spatial distribution of the population (red dashed line; center indicated with red cross), used to identify the radial position of each neuron reported in panel (e). **d,** Swarm charts of the dye ratio distribution from the 3 zebrafish larvae. Dots represent individual neurons. **e,** Density probability function (top) and angular spatial distribution (bottom) of the dye ratio values extracted from (c).

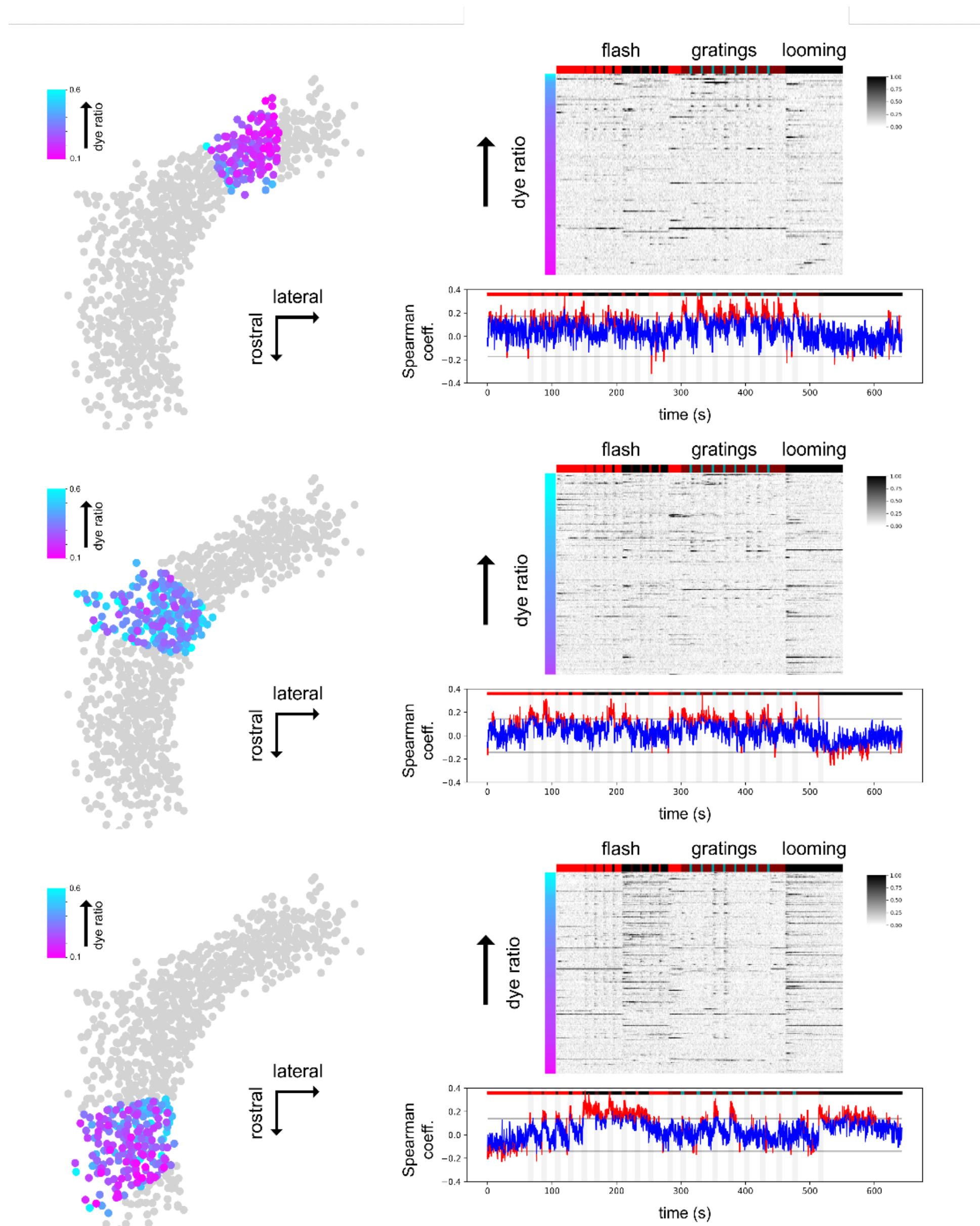

Supplementary Figure 9. Visual activity of early- and late-born PVN neurons. Left panels: Spatial distribution of PVNs color-coded according to the ratio of fluorescence intensity of the two dyes $JF_{552}/(JF_{552}+JF_{669})$, in smaller regions of the tectum. **Top right panels**: Raster plot of ΔF/F traces from individuals' cells (N = 5 larvae). Larvae were presented with visual stimuli, schematically represented by the upper color bar (red meaning high visual intensity, black indicating dark, and blue indicating moving stimuli). **Bottom right panels:** Spearman coefficient between dye ratio and ΔF/F, plotted in function of time. In red, sections in which the activity of the two populations show statistically significant differences. Thresholds for significance are indicated by the grey line (*Student's t* distribution: 0.1723 for caudal region, 0.18 for central region) and 0.14 for rostral region (N = 5 larvae).

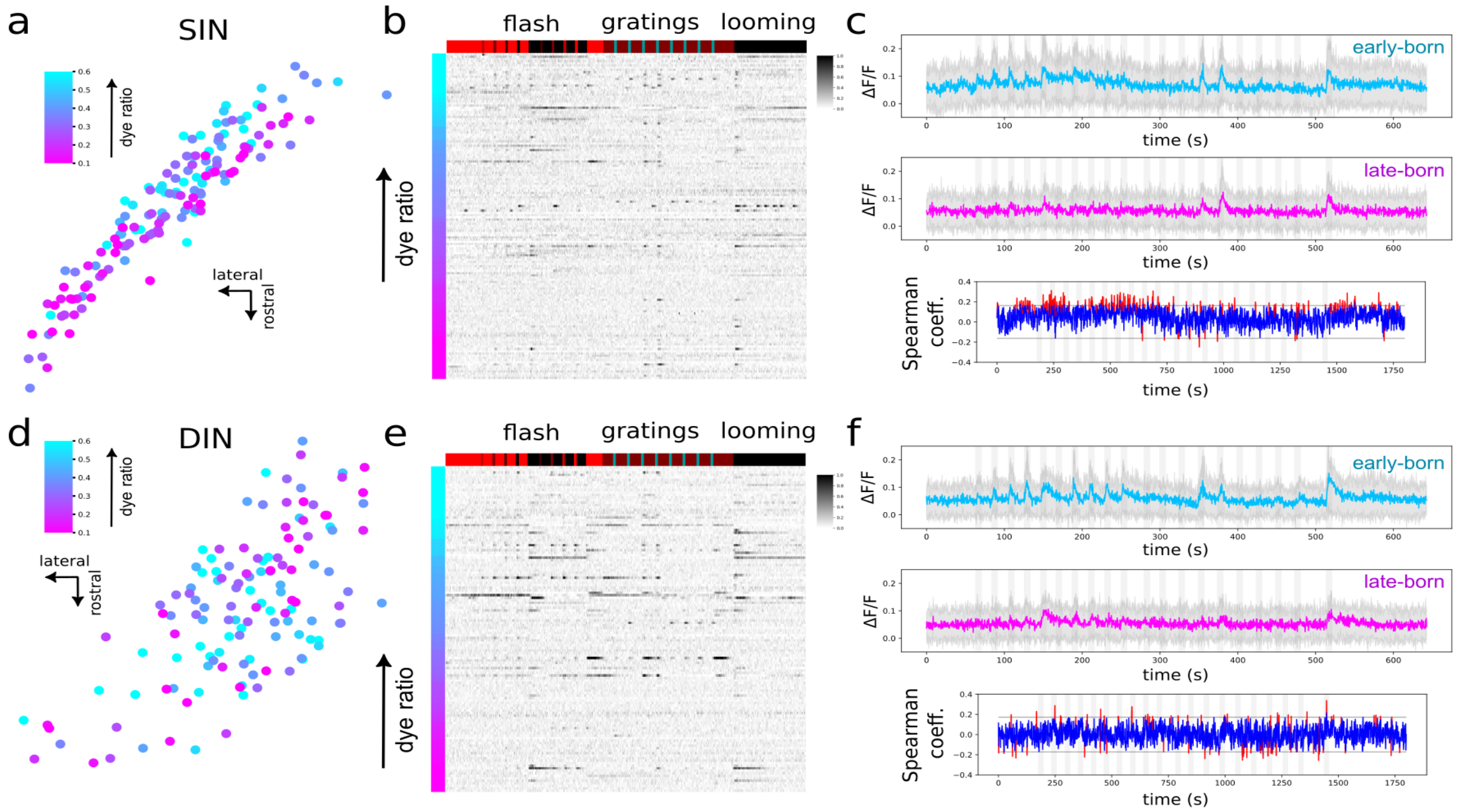

Supplementary Figure 10. Visual activity of early- and late-born neuropil neurons. a, Spatial distribution of SINs color-coded according to the ratio of fluorescence intensity of the two dyes $JF_{552}/(JF_{552}+JF_{669})$. Florescence ratio is used to cluster the cell population into early- and late-born neurons (as in Fig. 4). SIN and DIN are defined according to their spatial location (see Methods). **b,** Raster plot of ΔF/F traces from individuals' cells. Larvae were presented with visual stimuli, schematically represented by the upper color bar (red meaning high visual intensity, black indicating dark, and blue indicating moving stimuli). **c,**Top and middle: ΔF/F average traces from the early-born (cyan) and late-born (magenta) subpopulation of cells from (b). Semi-transparent rectangles indicate timing of visual stimuli. Traces are presented as the mean (colored lines) and the range between the 16th and 84th percentiles (gray areas). Bottom: Spearman coefficient between dye ratio and ΔF/F, plotted as a function of time. In red, sections in which the activity of the two populations show statistically significant differences (threshold for significance: 0.163, grey line). **d,** Spatial distribution of DINs color-coded according to the ratio of fluorescence intensity of the two dyes $JF_{552}/(JF_{552}+JF_{669})$. **e,** Raster plot of ΔF/F traces from individuals' cells. Larvae were presented with visual stimuli, schematically represented by the upper color bar (red meaning high visual intensity, black indicating dark, and blue indicating moving stimuli). **f,** Top and middle: ΔF/F average traces from the early-born (cyan) and late-born (magenta) subpopulation of cells from (b). Semi-transparent rectangles indicate timing of visual stimuli. Traces are presented as the mean (colored lines) and the range between the 16th and 84th percentiles (gray areas). Bottom: Spearman coefficient between dye ratio and ΔF/F, plotted as a function of time. In red, sections in which the activity of the two populations show statistically significant differences (threshold for significance: 0.172, grey line). SIN: superficial interneurons. DIN: deep interneurons. N = 5 larvae.

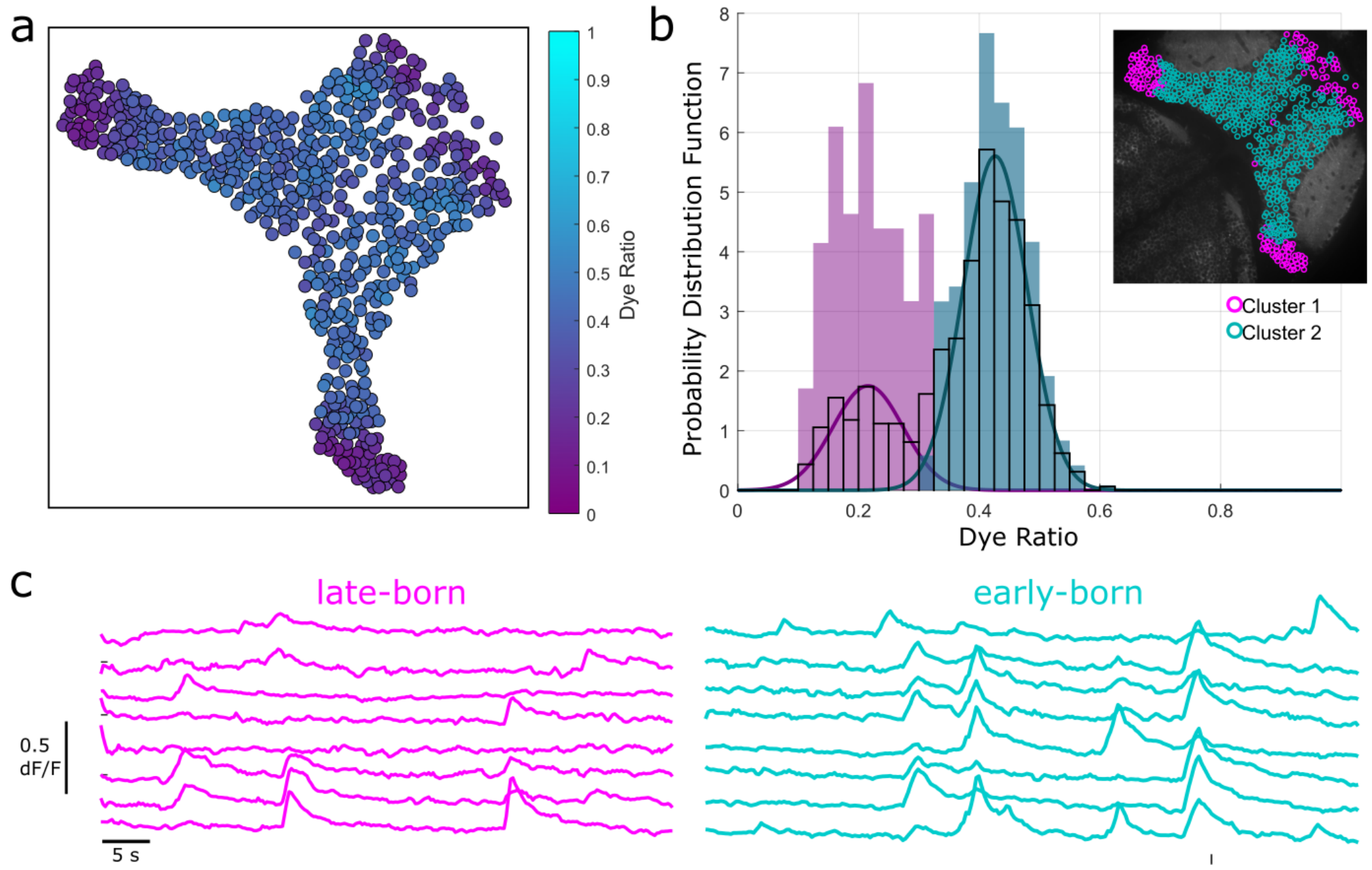

Supplementary Figure 11. Early and late-born clustering and calcium traces from WhaloCaMP expressing cells. a, Representation of dye ratio of tectal neurons expressing WhaloCaMP after dual-dye staining (corresponding to the main Fig. 6a). **b,** Probability-density histogram of the overall dye-ratio distribution (black outline), overlaid with the semi-transparent histograms and Gaussian components (solid lines) of the two k-means–based clusters, corresponding to early- (cyan) and late- (magenta) born neurons. Inset: spatial scatter plot showing the anatomical distribution of the two clusters. **c,** Representative calcium traces (extracted from the raster plot in the main figure) recorded from active neurons belonging to the early- and late-born populations. N = 1 fish, n = 164 cells from late-born (cluster 1), n = 480 cells from early-born (cluster 2).

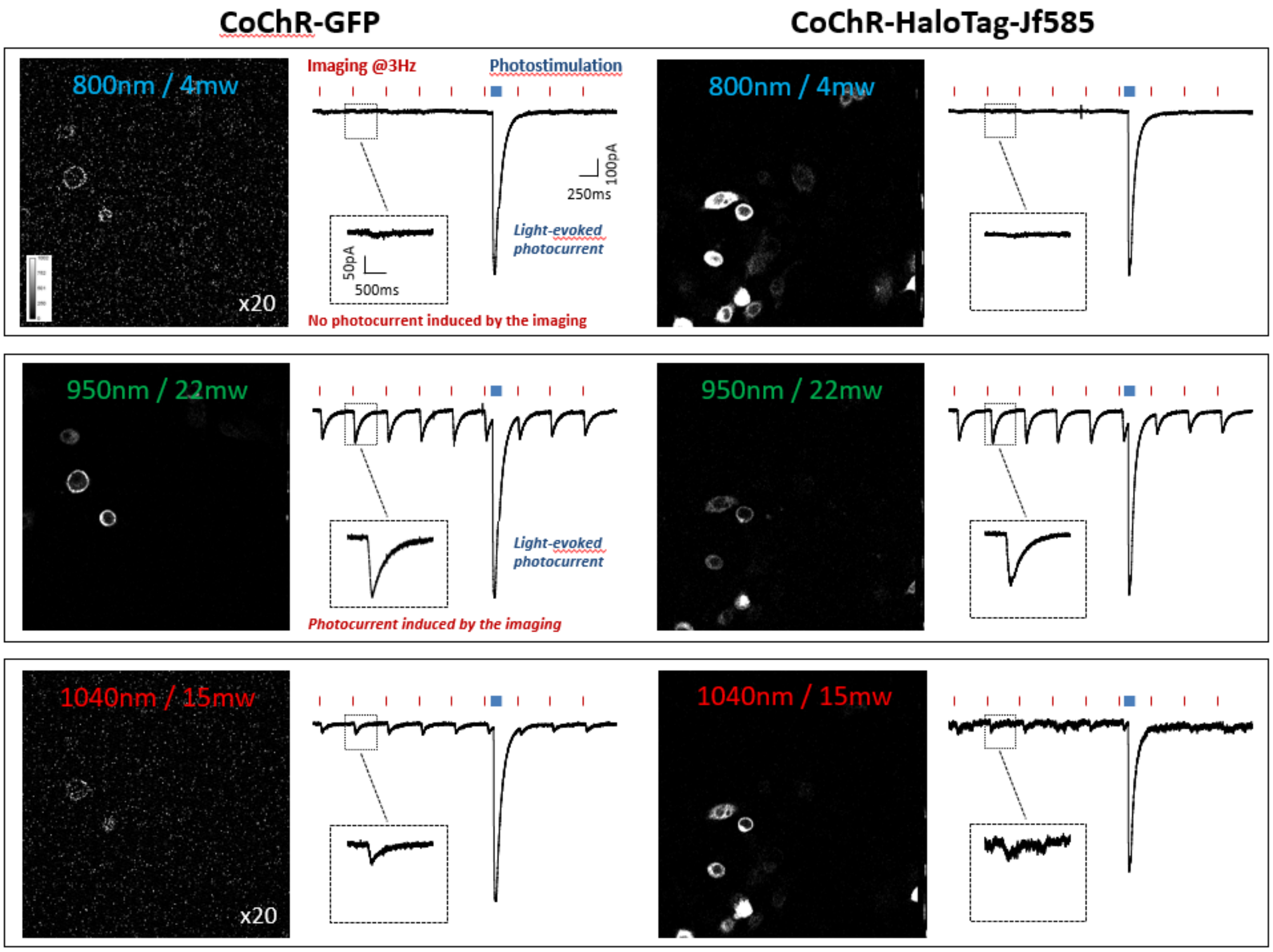

Supplementary Figure 12. Imaging-induced opsin stimulation. Images and measured photocurrents (patch clamp recording) from CHO cells expressing CoChR tagged with either GFP (left) or HaloTag-JF_{585} (right). Recordings are performed during 2P scanning imaging at different wavelengths and concurrent with holographic stimulation (blue tick). We observe how high contrast images of JF were achieved with low imaging power and using wavelengths outside of the CoChR absorption peak (800 and 1040 nm), therefore reducing the artefactual photocurrent induced by the imaging laser. The same wavelengths are much less efficient in imaging GFP, that requires 950 nm and high imaging power to achieve comparable contrast images, therefore inducing high artefactual photocurrent from the imaging laser. N = 3 cells for CoChR-GFP, N = 4 cells for CoChR-HaloTag.

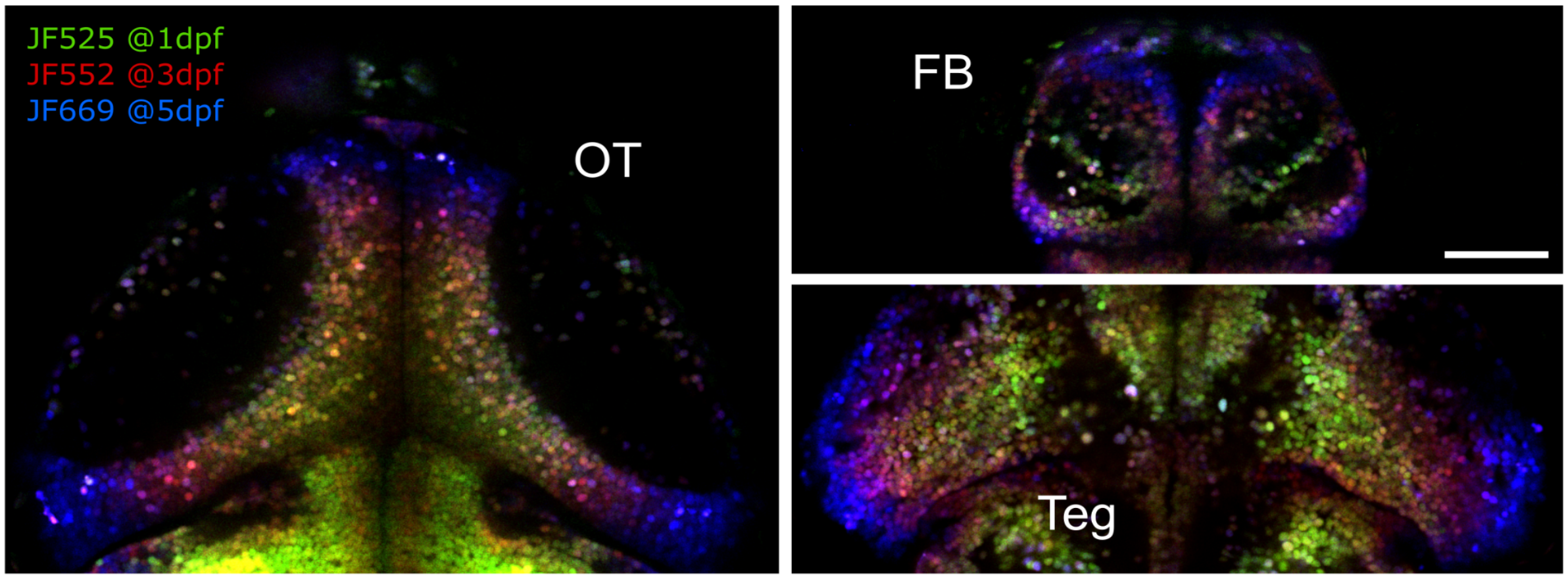

Supplementary Figure 13: Simultaneous visualization of multiple age cohorts, using standard lasers. Larvae were sequentially incubated with 3 dyes: JF_{525} (at 1dpf), JF_{552} (at 3 dpf), and JF_{669} (at 5 dpf), and imaged at 5dpf with 514 nm, 561 nm, and 640 nm lasers, respectively. The signals from the three dyes are clearly separable and result in similar topographical patterns observed with the white laser configuration. OT: Optic tectum. FB: Forebrain. Teg: Tegmentum. Scale bar: 50μm. N = 5 fish.

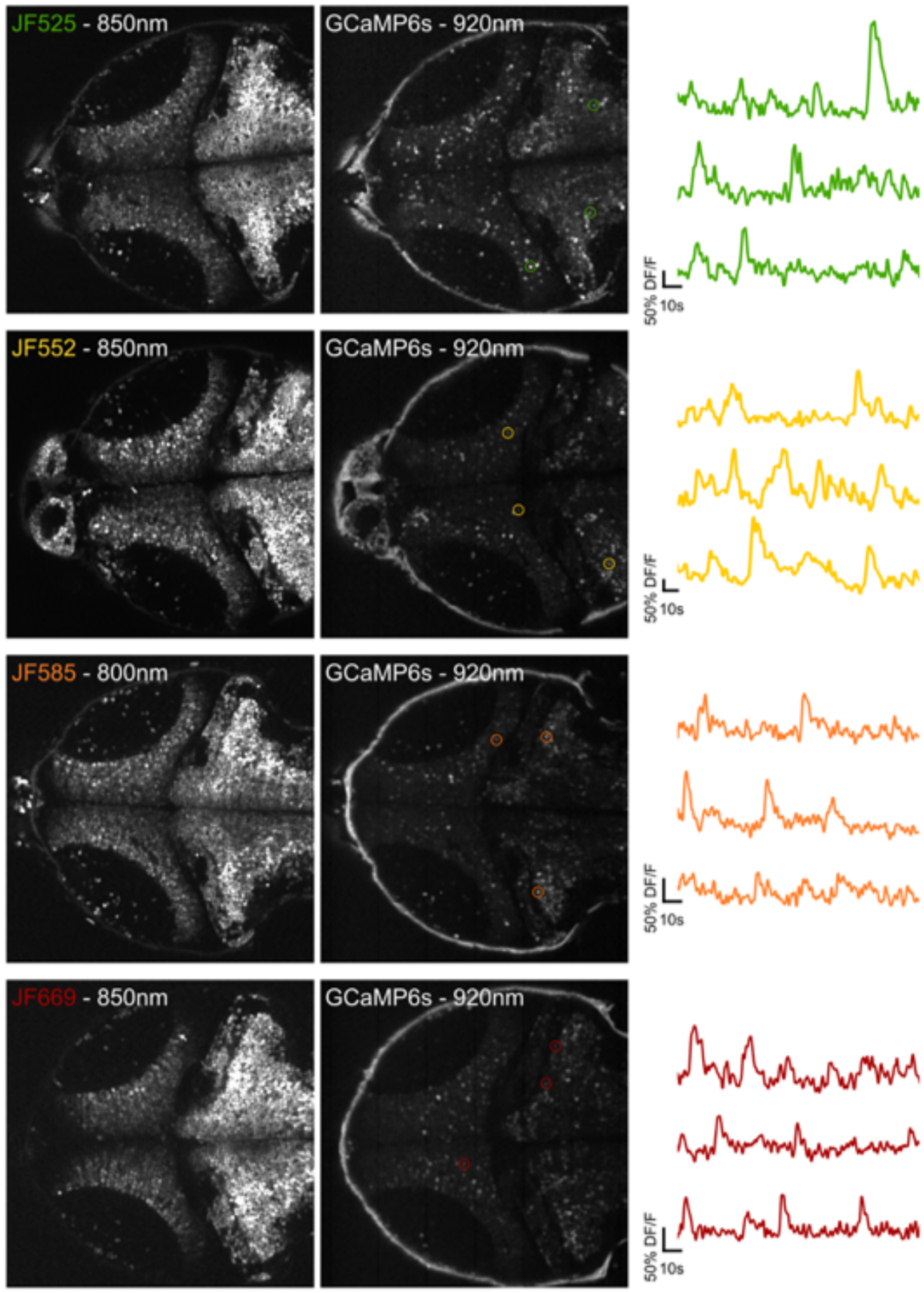

Supplementary Figure 14. Spectral crosstalk of GCaMP and JF dyes. Two photon images of the OT of larvae co-expressing HaloTag (incubated with different JF dyes; *Tg(Xla.Tubb2-hsp70-ubc:h2b-HaloTag)*) and GCaMP6s *(Tg(HuC:H2b-GCaMP6s)*. Imaging at 920nm excitation wavelength allowed detection of spontaneous calcium transients (circles indicate the cells of interest; right: exemplary calcium traces). N = 3 fish.

Xla.Tubb2-hsp70-ubc:H2B-HaloTag; he1.1:CFP	stable line + plasmid
elavl3:H2B-HaloTag	stable line + plasmid
Xla.Tubb2-hsp70-ubc:H2B-HaloTag-T2A-GFPcaax; he1.1:CFP	stable line + plasmid
elavl3:sypb-HaloTag	stable line + plasmid
elavl3:voltron1-Kv2.1	stable line + plasmid
elavl3:WhaloCaMP-eGFP	stable line + plasmid
14UAS:CoChR-HaloTag; myl7:GFP	stable line + plasmid
Xla.Tubb2-hsp70-ubc:CoChR-HaloTag; he1.1:CFP	plasmid
Xla.Tubb2-hsp70-ubc:HaloTag; he1.1:CFP	plasmid
14UAS:H2B-HaloTag; he1.1:CFP	plasmid
14UAS:HaloTag; he1.1:CFP	plasmid
Xla.Tubb2-hsp70-ubc:H2B-HaloTag-linker-GFP	plasmid
14UAS:CoChR-T2A-H2B-HaloTag ; he1.1:CFP	plasmid
Xla.Tubb2-hsp70-ub:CoChR-T2A-H2B-HaloTag; he1.1:CFP	plasmid
QUAS:voltron2; he1.1:CFP	plasmid
pAAV-SynCoChR-HaloTag	plasmid

Supplementary Table 1: Plasmids and transgenic lines generated.
