# Supplementary material for: CLOK: a chemigenetic multicolor labeling system to visualize neuronal birthdate and circuit integration
Source: Nat Neurosci. 2026 Sep 30;29(11):2861–73. doi: 10.1038/s41593-026-02423-9 (PMC13724276; doi:10.1038/s41593-026-02423-9)
Supplement: Supplementary file 2 [file 41593_2026_2423_MOESM2_ESM.pdf]

nature portfolio

Corresponding author(s): Filippo Del Bene
Minoru Koyama
Giulia Faini

Last updated by author(s): Jun 9, 2026

Reporting Summary

Nature Portfolio wishes to improve the reproducibility of the work that we publish. This form provides structure for consistency and transparency in reporting. For further information on Nature Portfolio policies, see our Editorial Policies and the Editorial Policy Checklist.

Statistics

For all statistical analyses, confirm that the following items are present in the figure legend, table legend, main text, or Methods section.

n/a	Confirmed	
☐	☒	The exact sample size (*n*) for each experimental group/condition, given as a discrete number and unit of measurement
☐	☒	A statement on whether measurements were taken from distinct samples or whether the same sample was measured repeatedly
☐	☒	The statistical test(s) used AND whether they are one- or two-sided<br>*Only common tests should be described solely by name; describe more complex techniques in the Methods section.*
☒	☐	A description of all covariates tested
☐	☒	A description of any assumptions or corrections, such as tests of normality and adjustment for multiple comparisons
☐	☒	A full description of the statistical parameters including central tendency (e.g. means) or other basic estimates (e.g. regression coefficient) AND variation (e.g. standard deviation) or associated estimates of uncertainty (e.g. confidence intervals)
☐	☒	For null hypothesis testing, the test statistic (e.g. *F*, *t*, *r*) with confidence intervals, effect sizes, degrees of freedom and *P* value noted<br>*Give P values as exact values whenever suitable.*
☒	☐	For Bayesian analysis, information on the choice of priors and Markov chain Monte Carlo settings
☒	☐	For hierarchical and complex designs, identification of the appropriate level for tests and full reporting of outcomes
☒	☐	Estimates of effect sizes (e.g. Cohen's *d*, Pearson's *r*), indicating how they were calculated

Our web collection on statistics for biologists contains articles on many of the points above.

Software and code

Policy information about availability of computer code

Data collection	Confocal images were acquired with Zeiss Zen Blue 2.6, Leica Application Suite X 4.8.0.28989 or Olympus FV31S-SW (version 2.61.243). Two-photon images were collected with ScanImage 2016, Imspector softwares and Slidebook 6 (3i, proprietary software). Electrophysiology data were recorded with NI DAQmx 19.0.0 on .NET Framework 4.6.2 using C# and with pClamp software (molecular device). Light-sheet data were collected with Micro-manager (version 2.0.3 20250327)
Data analysis	Fiji/ImageJ version: 1.54p, Pairwise Stitching: 1.20, MATLAB R2024b<br>Python 3.10.16, Rastermap 1.0, numpy 2.2.5, scipy1.15.3, matplotlib 3.10.3, scikit-image 0.25.2, scikit-learn 1.6.1, numba 0.6.1.2<br>Greedy 1.2.0 (commit 642a15c3e5735dd8413711af7d6d52dlcd8a25db)<br>R 4.4.3, Ime4 1.1-37, emmeans 1.11.1, ggplot2 3.5.2

For manuscripts utilizing custom algorithms or software that are central to the research but not yet described in published literature, software must be made available to editors and reviewers. We strongly encourage code deposition in a community repository (e.g. GitHub). See the Nature Portfolio guidelines for submitting code & software for further information.

Data

Policy information about availability of data

All manuscripts must include a data availability statement. This statement should provide the following information, where applicable:

- Accession codes, unique identifiers, or web links for publicly available datasets
- A description of any restrictions on data availability
- For clinical datasets or third party data, please ensure that the statement adheres to our policy

All date related to reported figures (including Supplementary Information figures) are available as Source Data files. Additional data are available upon request. Source data are provided with this paper.

Research involving human participants, their data, or biological material

Policy information about studies with human participants or human data. See also policy information about sex, gender (identity/presentation), and sexual orientation and race, ethnicity and racism.

Reporting on sex and gender	n/a
Reporting on race, ethnicity, or other socially relevant groupings	n/a
Population characteristics	n/a
Recruitment	n/a
Ethics oversight	n/a

Note that full information on the approval of the study protocol must also be provided in the manuscript.

Field-specific reporting

Please select the one below that is the best fit for your research. If you are not sure, read the appropriate sections before making your selection.

☒ Life sciences ☐ Behavioural & social sciences ☐ Ecological, evolutionary & environmental sciences

For a reference copy of the document with all sections, see nature.com/documents/nr-reporting-summary-flat.pdf

Life sciences study design

All studies must disclose on these points even when the disclosure is negative.

Sample size	No statistical method was used to predetermine sample sizes, but our sample sizes are similar to those generally used in the field.
Data exclusions	Subjects that did not develop at a normal rate were excluded.<br>The 3D volumes that remained misregistered after the optimization procedures described in the methods were excluded.<br>Functional imaging sessions with unstable EMG recordings or movements drifts were excluded.
Replication	All data were collected from more than two separate rounds of experiments. All attempts at replication were successful.
Randomization	For each experiment, fish were chosen at random from batches of healthy siblings that had developed normally, and, unless otherwise stated, all randomly sampled fish were included in the analyses; any exclusion criteria are specified in the corresponding sections.
Blinding	No treatment group exists in the current study. Thus, no blinding procedure was employed.

Reporting for specific materials, systems and methods

We require information from authors about some types of materials, experimental systems and methods used in many studies. Here, indicate whether each material, system or method listed is relevant to your study. If you are not sure if a list item applies to your research, read the appropriate section before selecting a response.

Materials & experimental systems

n/a	Involved in the study	
☒	☐	Antibodies
☐	☒	Eukaryotic cell lines
☒	☐	Palaeontology and archaeology
☐	☒	Animals and other organisms
☒	☐	Clinical data
☒	☐	Dual use research of concern
☒	☐	Plants

Methods

n/a	Involved in the study	
☒	☐	ChIP-seq
☒	☐	Flow cytometry
☒	☐	MRI-based neuroimaging

Eukaryotic cell lines

Policy information about cell lines and Sex and Gender in Research

Cell line source(s)	CHO cells (ECACC 85050302, purchased from Sigma-Aldrich, distributor in Europe for ECACC)
Authentication	CHO cells were authenticated by ECACC before purchase and only passed until P20 to avoid genetic drift
Mycoplasma contamination	Cell lines were not tested for mycoplasma contamination
Commonly misidentified lines (See ICLAC register)	No commonly misidentified cell lines were used in this study.

Animals and other research organisms

Policy information about studies involving animals; ARRIVE guidelines recommended for reporting animal research, and Sex and Gender in Research

Laboratory animals	Transgenic adults zebrafish (casper or cancb1 ts25/+ backgrounds) of age between 3 and 18 months were used to produce larvae. In each experiment larvae between 4 and 6 days post fertilisation were used as describe.<br>Juveniles zebrafish were used until 2 months old as described.
Wild animals	This study did not involve wild animals.
Reporting on sex	Larval zebrafish were studied before the onset of sexual differentiation. The sex of juvenile fish was not relevant for this study and not considered.
Field-collected samples	This study did not involve samples collected from the field.
Ethics oversight	All procedures were conducted in accordance with national and European (2010/63/EU) guidelines, the animal research guidelines from the Canadian Council on Animal Care and the University of Toronto Local Animal Care Committee. They were approved by the authors' institutional review boards and national authorities (French Ministry of Research, protocol ID: APAFIS #21323 and #49266; University of Toronto, protocol ID: 20012777).

Note that full information on the approval of the study protocol must also be provided in the manuscript.
